# Genome-wide assessment of genetic diversity and transcript variations in 17 accessions of the model diatom *Phaeodactylum tricornutum*

**DOI:** 10.1093/ismeco/ycad008

**Published:** 2024-01-10

**Authors:** Timothée Chaumier, Feng Yang, Eric Manirakiza, Ouardia Ait-Mohamed, Yue Wu, Udita Chandola, Bruno Jesus, Gwenael Piganeau, Agnès Groisillier, Leila Tirichine

**Affiliations:** Nantes Université, CNRS, US2B, UMR 6286, Nantes F-44000, France; Nantes Université, CNRS, US2B, UMR 6286, Nantes F-44000, France; Nantes Université, CNRS, US2B, UMR 6286, Nantes F-44000, France; Immunity and Cancer Department, Institut Curie, PSL Research University, INSERM U932, Paris 75005, France; Nantes Université, CNRS, US2B, UMR 6286, Nantes F-44000, France; Nantes Université, CNRS, US2B, UMR 6286, Nantes F-44000, France; Institut des Substances et Organismes de la Mer, ISOMer, Nantes Université, UR 2160, Nantes F-44000, France; Sorbonne Université, CNRS, Laboratoire de Biodiversité et Biotechnologies Microbiennes, LBBM, F-66650 Banyuls-sur-Mer, France; Nantes Université, CNRS, US2B, UMR 6286, Nantes F-44000, France; Nantes Université, CNRS, US2B, UMR 6286, Nantes F-44000, France

**Keywords:** genetic, transcriptomic, diversity, diatoms, Phaeodactylum tricornutum, heterozygosity, selection, ecology, evolution

## Abstract

Diatoms, a prominent group of phytoplankton, have a significant impact on both the oceanic food chain and carbon sequestration, thereby playing a crucial role in regulating the climate. These highly diverse organisms show a wide geographic distribution across various latitudes. In addition to their ecological significance, diatoms represent a vital source of bioactive compounds that are widely used in biotechnology applications. In the present study, we investigated the genetic and transcriptomic diversity of 17 accessions of the model diatom *Phaeodactylum tricornutum* including those sampled a century ago as well as more recently collected accessions. The analysis of the data reveals a higher genetic diversity and the emergence of novel clades, indicating an increasing diversity within the *P. tricornutum* population structure, compared to the previous study and a persistent long-term balancing selection of genes in old and newly sampled accessions. However, the study did not establish a clear link between the year of sampling and genetic diversity, thereby, rejecting the hypothesis of loss of heterozygoty in cultured strains. Transcript analysis identified novel transcript including noncoding RNA and other categories of small RNA such as PiwiRNAs. Additionally, transcripts analysis using differential expression as well as Weighted Gene Correlation Network Analysis has provided evidence that the suppression or downregulation of genes cannot be solely attributed to loss-of-function mutations. This implies that other contributing factors, such as epigenetic modifications, may play a crucial role in regulating gene expression. Our study provides novel genetic resources, which are now accessible through the platform PhaeoEpiview (https://PhaeoEpiView.univ-nantes.fr), that offer both ease of use and advanced tools to further investigate microalgae biology and ecology, consequently enriching our current understanding of these organisms.

## Introduction

Photosynthetic microalgae are important components of life in the oceans providing organic biomass and fueling a range of key biogeochemical processes. Diatoms in particular are widely recognized as one of the most significant phylum of phytoplankton, owing to their substantial contribution to primary productivity, carbon fixation, and biogeochemical cycling of essential nutrients such as nitrogen and silicon [1, 2]. In addition to their ecological importance, diatoms are a rich source of bioactive compounds with diverse applications in various industries, including nutraceuticals, nanotechnology, pharmaceuticals, and food and feed industries [3, 4]. In recent years, using model species in diatoms has dramatically increased our knowledge about the biology and ecology of these important organisms [5]. Particularly, one species, the diatom *Phaeodactylum tricornutum* has proven to be a robust model for research, yielding a wealth of knowledge and advancing our understanding in this field of research.

*P. tricornutum*, a marine pennate diatom is commonly found in coastal waters, including tidal areas, estuaries, rock pools, and shallow waters exposing the species to important fluctuations in light intensity and salinity. The diatom is a well-established model with a genome that has been fully assembled and well annotated, along with an expanding molecular toolbox using the reference strain Pt1 8.6 [6-10]. Genome-wide sequencing of 10 accessions of *P. tricornutum* (Pt1–Pt10) using Illumina identified diverse variations throughout the genome, including single nucleotide (SNPs),insertion deletion polymorphisms (INDEls), and copy number variations (CNVs) [11]. This study provided important insights into the genetic diversity of the isolates clustering them into four distinct clades with a conserved genetic and functional makeup. Previous studies have revealed distinguishing features among different accessions. Pt4 displayed a low nonphotochemical quenching (NPQ), Pt5 demonstrated higher adhesion, Pt6 exhibited substantial lipid accumulation, Pt8, Pt3, and Pt9 have different cell morphologies, and Pt3 demonstrated increased tolerance to variations in salinity, among other traits [12-14].

**Figure 1:**
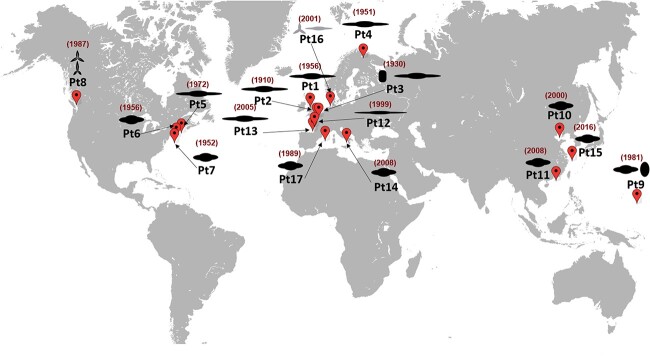
World map illustrating the sampling sites for the 17 *P. tricornutum* accessions analysed in the study with the year of sampling indicated in red.

The 10 sequenced accessions of *P. tricornutum* were mostly collected more than a century ago and have been preserved as either lab cultures or frozen stocks in culture collections for extended periods of time. Therefore, their genetic composition may have been affected, potentially favoring genes that are adapted to lab conditions [15-20]. Seven more recent isolates were collected from the environment and sequenced, Pt11 (Hong Kong, China), Pt12, Pt13 (both from Bourneuf Bay, West Atlantic, France), Pt14 (Gulf of Salerno, Italy), Pt15 (East China Sea), Pt16 (Helgoland, Atlantic Ocean, North Sea, Germany), and Pt17 (Banyuls Bay, Gulf of Lion, France) (Fig. 1, Supplementary Table S1). Assessment of genetic diversity within natural accessions of a model diatom, such as *P. tricornutum* is critical to our understanding of fundamental questions relevant to diatom’s biology and ecology. A high genetic diversity within the *P. tricornutum* species presents substantial implications, including valuable insights into their adaptive strategies across diverse ecological niches, alongside the identification of pivotal genetic determinants governing responses to environmental factors.

DNA sequence polymorphism can lead to phenotypic variations but it remains only the first step in understanding how these polymorphisms can affect the phenotype. Variations in transcript levels are another proxy to better understand the contribution of genes to phenotypic variations. Diatoms have developed sophisticated sensory and gene regulatory mechanisms to detect and respond to environmental cues. They employ transcription factors and regulatory elements to control the initiation and rate of transcription. Furthermore, posttranscriptional mechanisms, such as RNA splicing, RNA transport, stability, and translation, play essential roles in determining the abundance and activity of specific gene products. These integrated processes collectively enable diatoms to fine-tune gene expression in response to changing environmental conditions [7]. An illustrative example is the diel and circadian rhythms in gene expression, which are synchronized with light and dark cycles. These rhythmic gene regulation mechanisms enable diatoms to optimize their metabolic processes and growth in a time-dependent manner, playing a significant role in their overall physiology and ecology [21].

Differences in gene expression can be attributed to different DNA sequence polymorphisms including SNPs and INDELs that can nullify gene function or induce variations in splicing. It is important to ask whether the genes that show differences in expression are under selective pressure and whether there is a link between transcript level variations and DNA sequence polymorphisms. DNA sequence may not explain differences in gene expression; rather, such cases are known to be the consequences of epigenetic factors, including DNA methylation, posttranslational modifications of histones and small and long noncoding RNAs [22, 23].

In the present study, we analysed the genetic diversity of 17 accessions of the model diatom *P. tricornutum* by examining both DNA sequences and transcript levels. These accessions were collected from various coastal regions of world seas and oceans including recently sampled accessions that were not included in the previous study that had sequenced accessions collected over a span of 100 years. Our findings indicate a higher genetic diversity that defined more distinct clades and long-term balancing selection (BS) of genes in old and newly sampled accessions. However, our analysis failed to establish a clear link between the temporal factor of sample collection year and the extent of genetic diversity. Consequently, the hypothesis proposing a decline in genetic diversity, specifically the loss of heterozygosity in cultured strains over time, could not be supported [24]. Furthermore, our study identified novel transcripts among which various noncoding RNA species and provides insights into the regulation of genes mediated by genetic and transcript diversity. Our study offers easy and valuable access to these novel genetic resources, particularly focusing on a model species, through the PhaeoEpiView platform (https://PhaeoEpiView.univ-nantes.fr) [10]. This unprecedented accessibility provides a multitude of opportunities for exploring diverse ecological functions by leveraging the genetic diversity of this model organism, thereby expanding our understanding of the biology and ecology of microalgae.

## Material and methods

### Material used and growth conditions

Eighteen different accessions of *P. tricornutum* were acquired from the Provasoli-Guillard National Center for Culture of Marine Phytoplankton, Roscoff, and Nantes culture collections (Supplementary Table S1). All of the accessions were grown axenically using enhanced artificial sea water (EASW) [25] in batch cultures at 19°C, under 12/12 light–dark period with a light intensity of 70 μmol photons m^−2^ s^−1^.

### Growth curves

The cultures were grown in 30 ml of EASW with an initial concentration of 10^5^ cells/ml. Cell counts were measured using flow cytometry (CytoFLEX, Becman, USA), every 2 days for 20 days, with 1 ml of sample taken from each accession culture each time. After 7 days of culture, 1 ml from each of Pt11 to Pt17 were used for light microscopy to describe variant shapes and cell size using Axio inverted microscope (ZEISS, Germany). Photos were further analyzed using Zeiss software (ZEN 2.6).

### Pulse amplitude modulated variable chlorophyll a measurements

Variable fluorescence measurements were carried out using an Imaging PAM fluorometer (Walz) with a blue measuring light (450 nm), controlled by the software ImagingWin v2.46i (Heinz Walz GmbH, Effeltrich, Germany). The actinic and saturating light were also blue and provided by a fluorometer LED panel. The saturation pulse intensity was 6000 μmol photons m^−2^ s^−1^ for 0.8 s. Samples were dark-adapted for 1 h before carrying out any measurements. For the construction of rapid light-response curves (RLCs) [26], the samples were exposed to nine incremental intensities of actinic light with an irradiance step duration of 30 s. The photosynthetically active radiation 400–700 nm) steps used were: 0, 5, 19, 31, 37, 42, 47, 56, 75, 143, 280, and 519 μmol photons m^−2^ s^−1^. The first point of the RLC corresponds to the dark-adapted state, yielding the minimum fluorescence yield (*F_o_*) and the maximum fluorescence yield (*F_m_*), allowing the calculation of the maximum PSII quantum efficiency (*F_v_*/*F_m_*) as *F_v_*/*F_m_* = (*F_m_*-*F_o_*)/*F_m_*. The remaining light steps measured the fluorescence yield (*F′*), the maximum fluorescence yield (*F_m_′*) in the light-exposed state, and the effective PSII quantum yield at each experimental light level (E) as *F_q_′*/*F_m_′* = (*F_m_′*–*F′*)/*F_m_′*. Relative PSII electron transport rates were calculated as rETR(E) = *F_q_′*/*F_m_′*(E) × E and nonphotochemical quenching as NPQ = (*F_m_*–*F_m_′*)/*F_m_′*. Maximum relative electron transport rates (rETR_max_) were estimated by fitting the RLCs with the photosynthesis-light response model of Platt *et al*. [27], and maximum NPQ (NPQ_max_) was estimated by fitting the NPQ-light response model of Serodio and Lavaud [28].

### DNA extraction and PCR protocol

After 7 days of culture, cells were centrifuged at 4000 × *g* for 20 min and washed twice with one time phosphate buffered saline (1X PBS). DNA was isolated using a CTAB protocol as described previously [29, 30]. A volume of 1.5 ml of CTAB buffer (450 μl 10% CTAB, 420 μl of 5 M NaCl, 60 μl of 0.5 M ethylenediaminetetraacetic acid (EDTA), 150 μl of 1 M Tris HCL) preheated to 65°C was placed into a 2 ml plastic tube together with the diatom pellet and incubated for 1 h at 65°C. Subsequently, DNA was isolated using a chloroform isoamyl (24/1) after centrifugation for 10 min (12 000 × *g*). The upper phase was removed and incubated with 3.2 μg of RNase A for 1 h at 37°C. DNA was isolated again using chloroform isoamyl (24/1) after centrifugation (12 000 × *g*) to remove protein and RNA. The same volume of isopropanol and 8% of the total volume of ice-cold 7.5 M ammonium acetate were used for precipitation at −20°C overnight. Nucleic acids were recovered after centrifugation (12 000 × *g*) at 4°C and purified by absolute ethanol, then washed with 70% ethanol. DNA concentration was measured using NanoDrop ND-1000 spectrophotometer (Thermo Scientific, Wilmington, DE). PCR amplification was carried out on Mastercycler®nexus ×2 (Eppendorf, Germany) in 20 μl total volume including 9.6 μl Go *Taq*, 50 ng DNA, and 10 μM forward and reverse primers. The PCR program consisted of 95°C for 5 min, then 35 cycles of 95°C denaturation for 30 s, annealing at the appropriate temperature (56–62°C) for 30 s, 72°C extension for 30 s, and a final extension step at 72°C for 10 min. PCR products were electrophoresed on 1% agarose gel, and the gel images were acquired using EBOX CX5 System (VILBER BIO IMAGING, France). Primer sequences are listed in Supplementary Table S2.

### RNA extraction

A total of 300 ml exponentially growing cells were centrifuged at 4000 × *g*, 4°C for 20 min and immediately re-suspended in 500 μl TRIzol®Reagent (Invitrogen, Thermo Fisher Scientific, USA) and vortexed vigorously before being stored at −80°C. RNA was isolated using Trizol reagent as described previously [31]. Purity and quantity of RNA were assessed using NanoDrop ND-1000 spectrophotometer (Thermo Fisher Scientific, USA). To remove genomic DNA, RNA samples were treated with Ambion™DNaseI (Invitrogen, Thermo Fisher Scientific, USA), according to the manufacturer’s instructions. RNA was quantified using Qubit™ RNA BR Assay Kit, 500 assays (Invitrogen, Thermo Fisher Scientific, USA).

### DNA and RNA sequencing

Extracted DNA for Pt1 8.6 and Pt11–Pt17 was sequenced on Illumina Novaseq 6000 platform, using 250 bp paired-end reads. Yields for Pt1 8.6, Pt11, Pt12, Pt13, Pt14, Pt15, Pt16, and Pt17 were 5.9, 13.2, 6.3, 5.7, 8.0, 6.6, 5.9, and 6.6 million read pairs, respectively. Messenger RNAs for Pt1 8.6 and Pt1–Pt17 were sequenced in duplicates on Illumina Novaseq 6000 platform, using 150 bp paired-end reads. The RNA libraries were enriched for matured RNAs and sequenced in stranded mode, yielding between 15.1 and 25.7 million read pairs.

## Bioinformatics analysis

### Variant calling analysis

Paired-end Illumina libraries from each ecotype (Pt1–Pt17) were first trimmed using Trimmomatic [32] with “ILLUMINACLIP:adapters.fa:2:30:10:2:keepBothReads LEADING:20 TRAILING:20 SLIDINGWINDOW:4:20 MINLEN:40.” Reads were then mapped with BWA-mem2 2.2.8 [33] on *P. tricornutum* Phatr2 assembly (accession GCA_000150955.2). Mapping rates ranged from 94.70% for Pt8–99.36% for Pt13. Variant calling and filtering were performed with GATK package version 4.2.2.0, following GATK best practices [34]. In short, the HaplotypeCaller module was run with a call confidence of 30, a sample ploidy of 2, and double precision was activated for the pair-HMM algorithm. Variants that were called were functionally annotated by snpEff [35] with the *P. tricornutum* database v5.0 and transposable elements (TEs) annotation from Rastogi *et al*. [7], with an upstream/downstream region size set at 2 kb and gene putative loss-of-function (LOF) annotation activated. Only SNPs and INDELs were retained from annotated Variant Call Format (VCF). Finally, GATK’s VariantFiltration module was used on SNPs with the following filters: “QD<2.0; QUAL<30; SOR>3.0; FS>60.0; MQ<40” and on INDELs with: “QD<2.0; QUAL<30; FS>200.0.” INDELs of size above 50 bp were extracted with SelectVariant module and some of the longest were validated by Polymerase Chain Reaction (PCR).

### Fixation index computation

Fixation index (Fst) was computed with ANGSD 0.939 [36] between all 17 accessions possible pairs. First, to compare only regions where data were present for all samples, the callable genome size was defined where read coverage on reference was no <10X across all accessions. Allele frequencies were computed for all ecotypes using ANGSD “-doSaf 1 -GL 2 -minMapQ 1 -minQ 20.”Subsequently, the folded site frequency spectrum (2DSFS) was determined with “realSFS -maxIter 100 -fold 1” for all ecotypes combinations. Finally, we computed all pairwise Fst on the resulting indexes with “realSFS fst index -fold 1.” All Fst values were gathered in a matrix and displayed as a heatmap using the R [37] package Pheatmap 1.0.12 (https://scicrunch.org/resolver/RRID:SCR_016418).

### Population clustering

Callable SNPs and INDELs were analyzed with ADMIXTURE 1.3.0 [38] with a random seed of 12 345 679, activated cross-validation (CV), and a bootstrap value of 200. Numbers of ancestral populations (K-value) were tested from 1 to 17, and the CV error was plotted to select the K-value leading to the lowest CV error. A principal component analysis (PCA) was then performed on Q-estimates for K = 15, and estimated ancestral fractions were plotted with R.

### Copy number variation and gene loss analysis

For each ecotype (Pt1–Pt17), the raw number of mapped fragments from the Variant Calling Analysis BAM files was counted on each Phatr3 gene [7] using featureCounts [39] in unstranded paired-end mode, and reads were assigned to all their overlapping features (“-O” option). Genes with no counts were deemed as possibly lost. Raw counts were then normalized for each gene following the FPKM formula: FPKM_normalized_count = (gene_raw_count × 10^9^) / (gene_length × total_sample_counts). Similar to previous work [11], binary logarithm Fold Change (log2FC) was calculated as the log2 ratio of normalized count for each gene to the average (mean) normalized count of all the genes per accession. Genes with a log2FC ≥ 2 were considered as showing putative copy number variation (CNV) compared to the reference strain. Finally, lost genes and genes exhibiting CNV in only one accession were marked as ecotype-specific. Heatmap plots were made in R using Pheatmap 1.0.12 (https://scicrunch.org/resolver/RRID:SCR_016418) and UpsetR 1.4.0 [40] packages.

### Genes with loss-of-function

After variant annotation by snpEff, we used an in-house script to find the total and specific number of genes affected by LoF mutations for each ecotype (Pt1–Pt17). First, we selected genes with LoF variants alleles retained in the VCF annotation file whether they are homozygous or not. Then, we searched for the genes that are specific to each accession and considered the nonaccession specific genes common if shared by two or more accessions.

### Site frequency Spectrum analysis

A matrix of allele counts per ecotype was created from the variants called previously on the callable genome. Briefly, for each biallelic SNP, a value of 0, 1, or 2 was determined for each ecotype, depending on its ploidy (homozygous on reference allele, heterozygous reference/alternate alleles, or homozygous on alternate allele, respectively). Moreover, functional annotation as described previously and the affected gene (if applicable) were added for each SNP. Folded SFS was then calculated for each functional category of SNPs (nonsense, non-synonymous [NS], synonymous, intergenic), and the resulting data were plotted with R.

### Searching for signatures of balancing selection on nonsynonymous SNPs

One of the signature of BS is the excess of nonsynonymous polymorphisms segregating at intermediate frequencies [41]. Genes with <10 synonymous (S) + NS SNPs were filtered out from the allele counts matrix (see “*Site Frequency Spectrum (SFS) analysis*”). The ratio of NS versus synonymous diversity was estimated by Watterson’s theta θ assuming twice as many NS as synonymous sites (θ*w*NS/θ*w*S). This ratio, defined as “(Number of NS/2) / (Number of S),” was calculated for each of the remaining 9267 genes. The 91 genes with an excess of NS SNPs (showing a θ ratio over 3) were extracted and further investigated. Finally, the same process was performed ecotype-wise, with a number of genes with a θ ratio > 3 ranging from 34 in Pt14 to 62 in Pt4.

### Phylogeny of the 17 accessions

A matrix of genome-wide biallelic SNPs and INDELs allele counts per ecotype (0, 1, or 2 depending on the called ploidy of the variant, see “*Site Frequency Spectrum (SFS) analysis*”) was computed for 640 454 variants found in the population of Pt1–Pt17. The Canberra distance and average linkage functions were identified to produce the tree that best represented the matrix by the “find_dend()” method from the R library “dendextend” 1.16.0 [42]. Then, an unrooted neighbor-joining tree was built using “phangorn” R package 2.10.0 [43] on the Canberra distance matrix and colored according to the ecotypes clades.

### Expression analysis

After filtering raw data with the removal of adapters and low quality reads, clean reads were aligned against the reference genome using HISAT2 2.0.5 [44]. Reads were assigned to each transcript using the FPKM metric, which normalizes for differences in library size and gene length. To compare gene expression levels in different accessions, the graphical representation of the distribution of gene expression and FPKM levels in different samples has been performed using the ggplot2 R package (v3.4.0) [45]. To differentiate between coding and noncoding transcripts, the Coding Potential Assessment Tool (CPAT) (DOI: 10.1093/nar/gkt006), which is a convenient and rapid method to categorize transcripts based on their coding scores, was used. This algorithm relies on specific models to assign coding potential scores to individual transcripts. In our research, we employed models from human, mouse, and zebrafish. Consequently, a table was generated, presenting the coding potential outcomes for each input transcript.

### Principal component analysis

To elucidate the relationships between distinct accessions, we conducted PCA on the gene expression values using fragments per kilobase of exon per million mapped (FPKM) of all the samples. Specifically, we first computed the average FPKM value for two replicates of each sample, followed by a logarithmic transformation of this average value (log2(FPKM+1)). Ultimately, we represented the samples according to their expression levels. In our analysis, we evaluated how well the samples were represented in PCA using the cos2 (square cosine) metric as a measure of quality. A cos2 value closer to one indicates a stronger representation of the variable by the two displayed components.

### Repeats detection in novel transcripts

Reads from both replicates of Pt1–Pt17 RNAseq libraries were mapped with HISAT2 2.0.5 [44] using the default parameters and all the mapping information was combined. The reads were then assembled with StringTie 1.3.3b [46], and novel transcripts were kept. We screened these 656 novel transcripts for repeats with RepeatMasker (*RepeatMasker Open-4.0*. 2013–2015 <http://www.repeatmasker.org>) Galaxy Tool wrapper version 4.0.9 (slow settings with matrices for 43% GC content), using a manually curated database of 71 reference TEs of *P. tricornutum*. RepeatMasker was run on the public server at https://usegalaxy.org [47].

### Weighted gene correlation network analysis

The network was obtained using weighted gene correlation network analysis (WGCNA) R package (version 1.17) [48]. Before constructing the co-expression network, we filtered out genes having a row median <10 reads. The expression matrix was transformed with the variance stabilizing transformation (vst) function from DESeq2 R package (version 1.32.0) [49]. The sequencing steps for the network construction for *P. tricornutum* accessions have been described previously [50].

For Network construction, the WGCNA R package [48] was used to identify network modules from 36 RNA-Seq datasets representing expression data from 18 *P. tricornutum* accessions (two replicates per accession). First, the quality of the raw counting matrix was checked. A hierarchical clustering analysis based on the “average” method allowed us to identify the Pt1R2 as an outlier, so this sample was filtered out from further analysis.

## Results

### Phenotypic traits characterization

**Figure 2:**
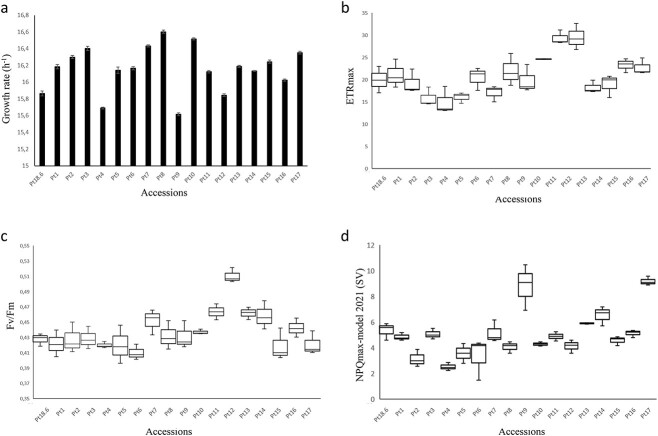
Growth and photosynthetic features for the 17 accessions; (A) growth rates in the 17 accessions, with error bars indicating standard deviations based on triplicate cultures; (B) mean maximum relative electron transport rate (rETRmax) for the 17 accessions; (C) the maximum PSII photochemical efficiency (F_v_/F_m_); (D) NPQ for the same 17 accessions.

To assess phenotypic differences among the 17 accessions, we monitored their growth, cell morphology, and photosynthetic features. Significant differences in growth rate were recorded at Day 4 of the exponential phase (Fig. 2A). In most of the cultures, cell growth rate dropped after Day 11 and entered the stationary phase. Pt8, Pt3, and Pt10 showed faster growth rate and higher final concentrations than other accessions, with 1165 × 10^4^, 1032 × 10^4^, and 960 × 10^4^ cells ml^−1^, respectively, in the end of exponential phase (*P <* .05). On the other hand, Pt4 and Pt9 showed slower growth rate than the others and the lowest final concentrations with 419 × 10^4^ and 559 × 10^4^ cells ml^−1^, respectively.

Cell dimensions were measured for only seven accessions (Pt11–Pt17) and compared to the previously published Pt1–Pt10 accessions [12]. Among all accessions, the previously measured Pt5 had the longest length (25–30 μm) and Pt14 cells were the shortest (10–15 μm). Pt12 had the thinnest cells with the lowest length/width ratio (9.9 ± 1.1), and Pt17 had the largest (4.6 ± 0.8) (Supplementary Table S1, Supplementary Fig. S1). Different morphotypes were observed in Pt16 (a mix of 75% fusiform and 25% of triradiate). As reported previously [11], we found few oval cells mixed with fusiform in Pt3 and Pt9 (7.3% and 7.6%, respectively). Triradiate cells were reported in Pt8 [12], but we did not observe triradiate cells in our conditions. Triradiate morphotype was reported to be instable in this accession [51].

### Measurements of photosynthetic parameters

To assess photosynthetic capacities of *P. tricornutum* accessions, we measured maximal PSII quantum yield (*F_v_*/*F_m_*) and maximal relative electron transport rates (rETRmax). Among all the accessions, Pt12 showed the highest *F_v_*/*F_m_* followed by Pt13 (*P* = .027) and Pt11 (*P* = .0047) (Fig. 2B). These three accessions also showed the best photosynthetic performances based on rETRmax (Fig. 2C), while Pt4, Pt5, and Pt6 demonstrated the lowest values in PSII quantum efficiency (Fig. 2C). The English Channel accessions, Pt1, Pt2, and Pt3 showed similar results.

To evaluate the response to excess light energy, we measured NPQ. Pt9 and Pt17 showed the highest NPQ capacity, ~8.2 and c. 9.2 respectively, while Pt4 showed a lower NPQ capacity of around 2.5 (Fig. 2D). These observations suggest that Pt9 and Pt17 can tolerate environments with higher light intensity compared to other accessions. This is consistent with their geographical distribution in latitudes that receive greater amounts of solar radiation. Similarly, Pt4 NPQ reflects an adaptation to its sampling location in higher latitudes, specifically the Norwegian Fjords.

### Variant calling analysis

**Figure 3:**
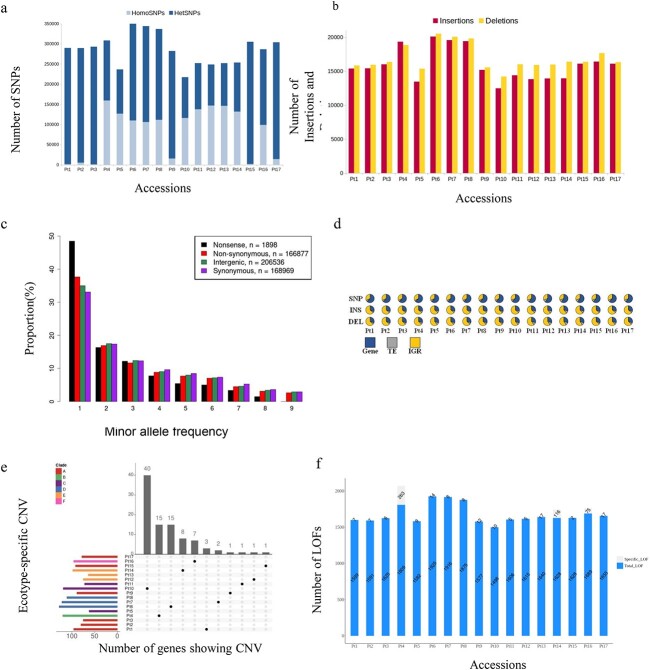
Genetic diversity across *P. tricornutum* accessions; (A) composition of heterozygous and homozygous SNPs, the stacked bar plot represents the number of SNPs discovered in Pt1–Pt17 accessions, showing a contrasted proportion of homozygous and heterozygous SNPs; homozygous SNPs are displayed in light blue, heterozygous SNPs are shown in dark blue; (B) the bar plot represents the number of insertions (red) and deletions (yellow) called in Pt1–Pt17 accessions; (C) folded SFS of 1898, 166 877, 206 536, and 168 969 nonsense, NS, intergenic, and synonymous SNPs, respectively; to obtain an unbiased SFS, only one accession was chosen to represent each group of genetically close accessions; these variants were thus called on Pt1-4-7-9-11-12-14-16-17; (D) pie charts represent different proportion of SNPs and INDELs over all functional features of the genome; GENEs (blue), TEs (gray), IGRs (intergenic regions, represented in yellow); (E) the bar plots represent the total number of genes considered to exhibit CNV per accession (left, colored by clade) and those that are accession-specific (top, in grey); only accessions having specific genes with CNV are shown in the matrix (center, in black); (F) the bar chart represents total and specific numbers of genes that are affected by loss-of-function (LoF) mutations for all ecotypes (Pt1—Pt17); the total number of genes (blue color) is the number of all nonduplicated genes on which a single variant (INDEL or SNP) was taken into account; the grey bars represent the number of genes unique to a specific accession and not present in the others.

We performed variant calling analysis on previously published sequences of Pt1–Pt10 [11] and the newly sequenced Pt11–Pt17 accessions using the reference strain Pt1 8.6 genome sequences. All the accessions had a good sequence coverage allowing a confident variant calling. We identified 731 357 single nucleotide polymorphisms (SNPs), 44 470 insertions (from 1 to 422 bp length), and 52 867 deletions (varying from 1 to 274 bp) (Fig. 3A and B). The SFS, which reflects the numbers of variants segregating at different frequencies, showed the expected excess of low frequency alleles (Fig. 3C). The highest increase in low frequency SNPs was observed in nonsense polymorphisms. NS polymorphisms showed the second highest increase, when compared to intergenic and synonymous polymorphisms. Most of the SNPs (59%–63%) were found in genes, while INDELS were mostly detected in intergenic regions (Fig. 3D). Our analysis identified 224 253 additional SNPs and 74 918 additional INDELs in Pt1–Pt10 compared to our previous study [11]*.*

Despite the higher number of discovered SNPs and INDELs, the overall trend of their distribution among the 10 previously analyzed accessions remains the same. Across all the accessions, most of the SNPs were heterozygous (HetSNPs) with > 95% in Pt1, Pt2, Pt3, Pt9, Pt15, and Pt17 and 65%–68% in Pt6, Pt7, Pt8, and Pt16, and the lowest proportion of HetSNPs was found in Pt4, Pt5, and Pt10–Pt14 (<49%). Across all the accessions, the numbers of INDELs were similar except for Pt4, Pt6, Pt7, and Pt8, which showed the highest number of INDELs (Fig. 3B, Supplementary Table S3). Most INDELs were shared among the accessions. A total of 14 INDELs were validated by PCR for randomly chosen loci (Supplementary Fig. S2). To further assess genetic diversity, we investigated CNVs and gene loss (GL). A total of 284 and 180 genes show CNV or GL, respectively. Most of the CNVs are shared and 11 accessions out of 17 show specific CNVs with 40 genes in Pt10 followed by Pt4, Pt6, Pt14, and Pt16 with 15, 15, 8, and 7 genes, respectively (Fig. 3E, Supplementary Tables S4 and S5). Randomly chosen loci were validated by PCR for GL (Supplementary Fig. S3).

To understand the functional impact of genetic variations among *P. tricornutum* accessions, we examined the LoF mutations such as premature stop codons, frameshifts, and start loss*.* A total of 31 536 LoF was found, among which 588 were shared across the accessions (Fig. 3F). Accession-specific LoFs were mostly found in Pt4 (61), Pt14 (20), and Pt16 (17) (Supplementary Table S6). LoF mutations were enriched in gene ontology (GO) categories of molecular function type (Supplementary Table S6) and in genes that belong to large gene families as previously shown [11]*.*

### Population structure and phylogeny of *P. tricornutum* accessions

**Figure 4:**
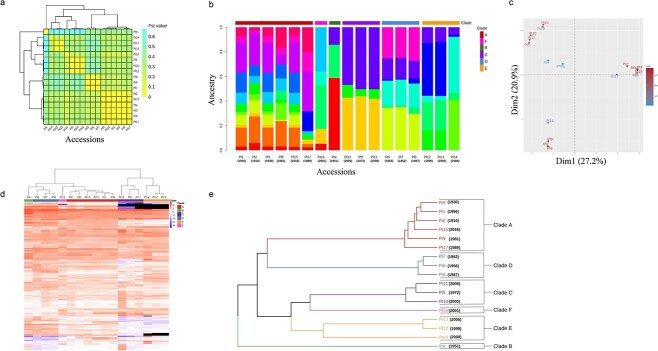
Clustering of *P. tricornutum* accessions into clades; (A) the heat-map shows the genetic differentiation or association between all possible pairs of accessions; the colors indicate Fst values, which range from 0.02 to 0.4, with a color gradient from yellow to green, respectively; values closer to 0 signify close genetic makeup and values closer to one indicate strong genetic structuring between the populations; (B) ADMIXTURE plot representing the ancestry genome fractions of Pt1–Pt17 (in color) for K = 15; (C) PCA showing the distance between the 16 accessions based on their shared genome structure; (D) the heatmap shows the log2 fold change (log2FC) of normalized reads counts between each reference gene and average of the read counts of all the reference genes per accession; FPKMs are used as normalized values, the log2 ratio of each gene FPKM over the mean FPKM of each accession being calculated; a blue to red color gradient in the heatmap represents low to high log2FC; the previously described clades A, B, C, D and the newly defined clades E and F are shown as colored annotations on the top; genes having a null FPKM in a given accession (considered lost) are displayed in black; only genes having a log2FC over 2 in at least one ecotype are plotted in this figure (222 genes) and are considered to exhibit CNV; (E) phylogenetic association of the 17 accessions based on genome-wide biallelic SNPs and Indels (640 454 variants), built from a hierarchical clustering (Canberra distance and average linkage functions).

To examine the global population structure of *P. tricornutum* accessions, we used pairwise fixation index (Fst), a measure of genetic differentiation revealing groups with low Fst index (<5%) (Fig. 4A). To further estimate genetic relatedness among *P. tricornutum* accessions, we used admixture proportion inference, which allows the assignation of individual genetic variations into clusters based on shared allele frequency patterns [38]. We ran ADMIXTURE with various plausible values of *K*, representing the number of source populations and found a stable admixture pattern with proportions at K = 15, reflecting the number of ancestral populations (Supplementary Fig. S4, Supplementary Table S7). Based on individual ancestry and the similarity of clusters between accessions, we distinguished 6 clades: Pt1, 2, 3, 9, 15, and 17 in Clade 1, with most of the clusters (up to 11) reflecting a clade with multiple ancestral populations, Pt16 in Clade 2, Pt4 in Clade 3, Pt5, 10, and 11 in Clade 4 with only three clusters, Pt6, 7 and 8 in Clade 5 and Pt12, Pt13, and Pt14 in Clade 6 (Fig. 4B).

To confirm admixture analysis clades, we performed a PCA, which revealed similar results reflecting common ancestry, except for Pt14, which is far from its admixture-defined Clade 6 (Fig. 4C). Of note, cluster composition proportions of Pt14 are different from Pt12 to Pt13. To further assess the segmentation among *P. tricornutum* accessions, we used CNVs to run a hierarchical clustering and found that the 17 accessions fall into 6 clusters supporting further both PCA and admixture analyses: Pt1, Pt2, Pt3, Pt9, Pt15, and Pt17 in Cluster 1, Pt16 in Cluster 2, Pt4 in Cluster 3, Pt6, Pt7, and Pt8 in Cluster 4, Pt5, Pt10, and Pt11 in Cluster 5 and Pt12, Pt13, and Pt14 in Cluster 6 (Fig. 4D). Phylogenetic analysis at whole genome scale using genetic variations (SNPs and INDELs) of the 17 accessions supported further the clustering into six clades observed with Fst and PCA analyses (Fig. 4E).

### Balancing and relaxed selection in Pt clades

We calculated the ratio of nonsynonymous to synonymous nucleotide site diversity using Watterson’s estimate of theta (θ*w*N/θ*w*S) [52] as a measure of the efficiency of natural selection. Ratios >3 suggest that selective pressure maintains NS polymorphisms, a signature for BS which refers to selective processes by which alleles are maintained in a population at frequencies larger than expected from genetic drift alone [53]. Conversely, θ*w*N/θ*w*S ratios <1 refer to NS polymorphisms are counter-selected, pointing to a purifying selection. We identified 91 common genes under BS and 2422 under purifying selection (Supplementary Table S8). Most of the genes under BS are of unknown function. However, those with known function were enriched in genes coding for functions such as cell proliferation and growth, perception, transmembrane transport activity, stress responses, and adaptation to the environment.

### Identification of transcript level variations and co-expression network modules in *P. tricornutum* accessions

Differences in gene expression are known to control inter- and intra-specific phenotypic variations, providing living organisms with abilities to colonize different ecological niches. To identify differences in transcriptomes, RNAs from *P. tricornutum* accessions (Pt1–Pt17) were sequenced and mapped to the reference genome. The mapping of mRNA-Seq reads was above 85% for all replicates except for one, Pt16R2, which had a mapping rate of 41.27%. Pearson correlation coefficients between each of the two replicates were mostly around 0.98 (Supplementary Fig. S5). Interestingly, a total of 656 genes including 25 from the chloroplast were novel. These genes are widely distributed over the genome among which some were specific to each of the ecotypes, and 385 genes were found to be common to all of them (Supplementary Table S9). They showed an average length of 709.42 bp, with334 genes categorized as sense and 322 genes as antisense. Except from few genes that were annotated, most of these novel genes were of unknown function (Supplementary Table S9). The majority of novel genes were downregulated compared to the average gene expression but their expression remains significant and cannot be considered as part of a spurious phenomenon of background low-level transcription.

The analysis of novel transcripts using RepeatMasker revealed that 12.26% of them contained repetitive elements, primarily Copia LTR_retrotransposon of Class I and rare MuDR2 Terminal Inverted Repeats of Class II (Supplementary Table S10). Additionally, ~ 0.48% of the transcripts contained simple repeats. Using a CPAT, we identified several noncoding RNA with sizes varying between 202 and 8379 bp (Supplementary Table S11). Furthermore, we detected several other RNA types, including sRNA, miRNA, tRNA, snoRNA, antisense, and piwiRNAs (Supplementary Table S11).

**Figure 5:**
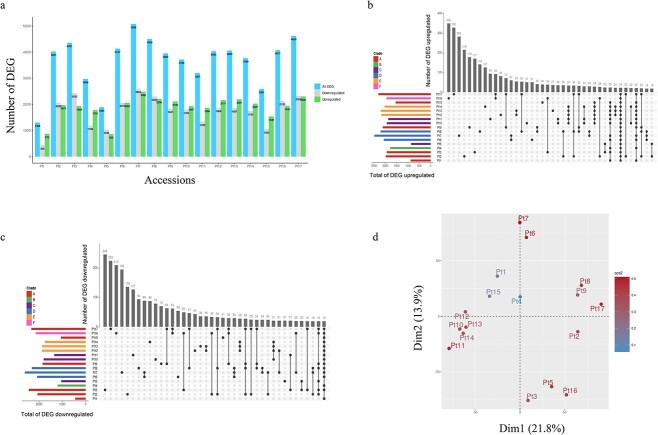
Transcription levels variations in the 17 accessions; (A) the bar plots represent the number of DEG in the 17 accessions denoted on the *y*-axis; all DEGs are displayed in blue; downregulated genes are shown in grey and upregulated in green; (B) the bar plots represent the total number of upregulated genes in each accession (left, colored by clade) and those that are specific to a given group of accessions (top, in grey); (C) the bar plots represent the total number of downregulated genes in each accession (left, colored by clade) and those that are specific to a given group of accessions (top, in grey); (D) PCA showing the distance between the 17 accessions based on gene expression values.

Then, we examined differentially expressed genes (DEGs) under our standard growth conditions by analyzing RNA-Seq data across the 17 accessions and comparing them with the reference strain Pt18.6. This strain was derived from Pt1, which displayed the lowest number of DEG (1308 genes) as expected (Fig. 5A). In contrast, Pt7 showed the highest number of DEG (5086 genes). The remaining ecotypes showed variable numbers of DEG, with Pt5 exhibiting the lowest number at 1890 genes. On average, most ecotypes had ~4000 DEG. With the exception of Pt1, which exhibited a bias toward upregulation (twice as many upregulated genes as downregulated genes), the other ecotypes displayed a balanced ratio of upregulated and downregulated genes. The majority of upregulated genes, their log2 (FoldChange) values vary between 1 and 3, while the majority of downregulated genes, the value of log2 (FoldChange) varies between −3 and −1. A substantial fraction of DEGs, regardless of their upregulation or downregulation status, is found to be specific to one or multiple ecotypes, suggesting an ecotype-dependent gene expression pattern that likely underlies ecotype-specific traits (Fig. 5B and C, Supplementary Tables S12 and S13). On the other hand, only a minor subset of DEGs displaying upregulation or downregulation were shared across all the ecotypes. We conducted a PCA using the average replicates expression to evaluate whether the ecotypes exhibited comparable expression profiles. Our analysis distinguished five clusters that partially aligned with the clades defined in this study, implying a correlation to some extent between genetic diversity and expression patterns (Fig. 5D).

To explore the relationship between phenotypic traits, specifically photosynthesis and transcripts, we closely examined the NPQ response and the genes related to its regulation in response to light. Indeed, the NPQ capacity depends on transthylakoidal proton gradient, but also on antenna proteins called light-harvesting complex protein X (LHCX) and on the Diatoxanthin Xanthophyll cycle [54], equivalent to the Zeaxanthin cycle in land plants [55]. Among the LHCX genes, only LCHX1 shows strong expression in all ecotypes, confirming its constitutive role in low light, while LHCX2 and 3 are involved in high light and LHCX4 expression increases in the dark [56] (Supplementary Fig. S6). Similarly, all genes involved in the Diatoxanthin Xanthophyll cycle show negligible expression in our low light culture conditions.

To understand the underlying nature of the conserved transcriptomic responses, we analyzed the enrichment of GO terms for both upregulated and downregulated DEGs (Supplementary Fig. S7). Additionally, we performed GO enrichment analysis on genes that were specifically upregulated or downregulated in a single accession as well as per clade, where applicable. Only a few GO terms emerged from the analysis of accession-specific DEGs, namely photosynthesis GO-related terms (light harvesting, protein chromophore linkage) in Pt8, lipid metabolic processes, and translation in Pt3 for downregulated genes, while upregulated genes were enriched in ribosome biogenesis and rRNA processing in Pt7, protein transport in Pt16, and glucose metabolism in Pt11 (Supplementary Fig. S7, Supplementary Tables S12 and S13).

**Figure 6:**
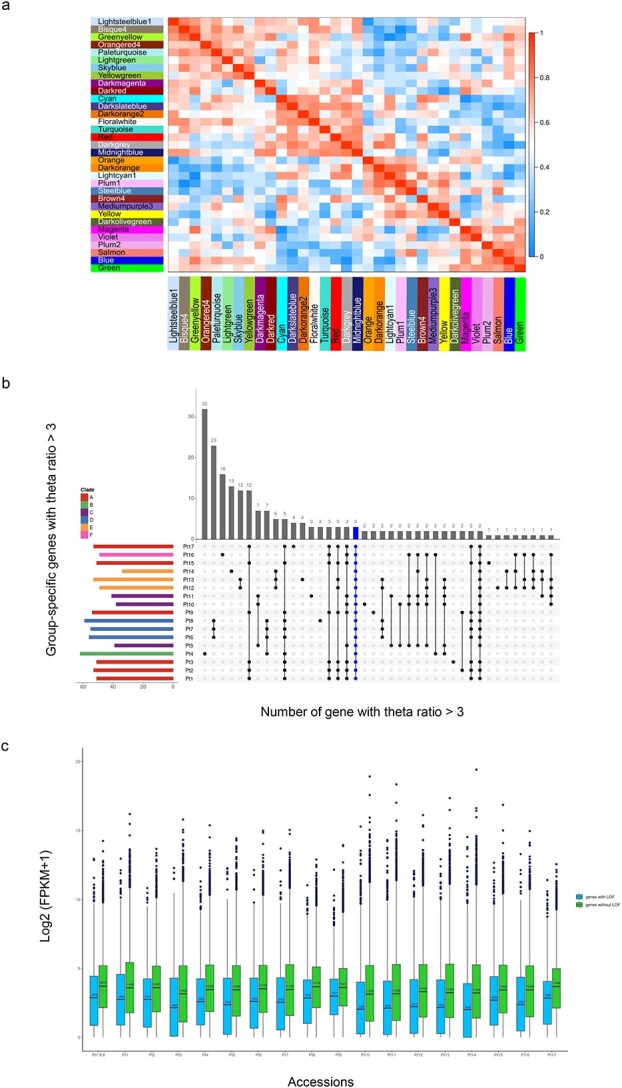
BS, LOF, and WGCNA analyses; (A) the plot represents the total number of genes showing a signature for BS per accessions (left, colored by clade) and those that are found only in a given group of accessions (top, in grey); genes under BS in all accessions are shown in blue; (B) distribution of gene expression levels in genes affected by loss-of-function mutations (blue) and unaffected genes (green) for each accession using FPKM values; (C) Eigen gene adjacency heatmap of the 33 merged modules of *P. tricornutum* accessions network; each row and column in the heatmap corresponds to one module (represented by their color); the scale bar on the right represents the correlation strength ranging from 0 (blue) to 1 (red).

The WGCNA package was used to construct gene co-expression network of transcripts from an expression matrix of ~432 000 transcripts derived from 36 RNA-seq samples, with 2 replicates collected from the 18 accessions including the reference strain Pt1 8.6. This approach yielded in 33 distinct co-expressed modules (labeled by different colors) with dark slate blue and plum2 modules containing each the smallest number of genes (106) and green yellow with the largest number of genes (1599) (Fig. 6C, Supplementary Table S14). These modules were constituted by genes demonstrating analogous expression profiles, which may or may not be consistent among different clades, suggesting the existence of additional factors besides genetic polymorphisms that could modulate transcript levels (Supplementary Fig. S8). The modules were further categorized into six distinct clusters, each characterized by a group of genes exhibiting comparable expression patterns thus implying their involvement in shared pathways (Fig. 6C, Supplementary Table S15). Based on the GO annotation analysis, we identified significant functional enrichments in different groups. Group I displayed a substantial increase in oxidoreductase activity, while Group II showed an enrichment in calcium ion binding activity. Group III was characterized by an enrichment in chlorophyll binding and light harvesting, whereas Group IV was associated with RNA processing and translation. Group V showed a significant enrichment in photosynthesis and cell redox homeostasis, while Group VI exhibited an enrichment in cell division and DNA binding activity (Supplementary Table S15).

## Discussion

The present study was designed to comprehensively characterize the phenotypic, genetic, and transcriptomic diversity among 17 distinct *P. tricornutum* accessions, which were collected from various locations across the world’s oceans and included more recently sampled accessions compared to those examined in prior studies [11, 12]. Growth dynamics, cell morphology, and photosynthetic traits were monitored, and significant inter-accession differences were recorded. Notably, the Pt4 and Pt9 strains exhibited a distinct growth pattern, implying a probable correlation to their respective sampling locations. More specifically, Pt4, sampled from the Norwegian fjords, seems to be adapted to a lower light intensity regime than that employed in our study, while Pt9, a tropical strain, showed a slower growth at 19°C compared to the presumed higher temperature in its geographical location. Moreover, the evaluation of photosynthetic abilities across various accessions corroborated the association with the sampling sites. Pt4 displayed the smallest rETR_max_ and PSII maximum quantum efficiency, while Pt12, Pt13, and Pt14 collected from the Mediterranean Sea and Atlantic side demonstrated higher photosynthetic performance, as evidenced by their important F_v_/F_m_ and rETR*_max_* values. Strains within the same clade show similar growth and photosynthetic performances, but there is no apparent correlation pattern with the year of sampling. Each clade includes accessions from both older and more recently collected samples. Measuring cell dimensions in the recently acquired accessions and their comparison with previously characterized ones revealed notable variations. Specifically, Pt12, Pt14, and Pt17 exhibited significant deviations, with Pt12 having the shortest cell length, Pt14 displaying the smallest length-to-width ratio, and Pt17 showing the largest cells when compared to the remaining accessions. Additionally, our investigation identified Pt16 as a new accession with a combination of triradiate and fusiform morphotypes. These disparities in cell sizes may potentially confer an advantageous trait in terms of enhanced gliding capabilities and improved photosynthetic efficiency [57]. The observed differences in cell dimensions and, at times, morphology are not surprising, given that *P. tricornutum* does not rely on silica for growth. This lack of dependence on silica may confer flexibility in morphogenesis, a trait not typically observed in silicified diatom species. The variations in cell sizes signify an adaptation to local environments, highlighting an environmental-induced control of morphogenesis rather than a genetic one, as demonstrated previously [51]. Drill-core records from Lake Titicaca in Peru revealed a strong correlation between size trends in the diatom *Cyclostephanos andinus* and environmental variables. This suggests that diatom size responds to regional environmental changes driven by global processes that affect lake level and thermal stratification [58]. This, in turn, implies that environmentally mediated epigenetic changes modulate phenotypic traits within the same species.

Variants calling analysis showed a larger number of SNPs and INDELs than previously reported in Pt1–Pt10. This is due to the gapped alignment mode used for SNPs and INDELs calling which performs better than un*gapped mapping* [59]*.* Improvements made to HaplotypeCaller’s algorithm since 2018 were also likely playing a role in the gain of sensitivity we noticed*.* Most of the SNPs were located in coding regions, while INDELs were mostly found in intergenic regions as a consequence of their highly deleterious effects within coding regions. Most of the SNPs were found to be heterozygous, indicating that *P. tricornutum* has a high level of heterozygosity. Our previous study has demonstrated a substantial level of heterozygosity in *P. tricornutum* populations. The recent sampling of genetically distinct accessions has reaffirmed the persistence of this trait, despite not being related to the previously identified highly heterozygous accessions Pt1, Pt2, and Pt3, thereby supporting the reliability of the heterozygosity measure. This high level of heterozygosity is intriguing in *P. triconrutum* considering that the species is not known to reproduce sexually, suggesting an advantage of heterozygous alleles or the detrimental homozygous alleles that get selected against, as reported in inbred population of clonal honey bees, *Apis mellifera capensis*, which retained after 20 years of inbreeding high heterozygosity throughout its genome due to selection against homozygotes [60]. Similar examples of heterozygosity advantage through its maintenance at high proportions of the genome were reported in other species, isolated wolf populations, and a hermaphrodite worm after several generations of selfing [61, 62]. An alternative explanation for the observed high heterozygosity could be due to the mutations that occurred in the ancestral lineage of Pt1, Pt2, Pt3, Pt9, Pt15, which was revealed through admixture analysis, indicating that these accessions share a common ancestry and are closely related to Pt17, which also exhibits high heterozygosity. In contrast, accessions with lower heterozygosity display a distinct ancestry pattern.

The SFS analysis provided compelling evidence consistent with the predictions of the nearly neutral theory of evolution, revealing an excess of low frequency alleles. This prevalence of lower frequency alleles in nonsense and NS polymorphisms can be attributed to the effects of purifying selection, which acts against deleterious mutations. Consequently, our investigation aimed to examine whether there was an elevated occurrence of NS mutations in the Pt1 8.6 reference strain, in contrast to both the original Pt1 accession and the closely related Pt2 strain within the same clade. This analysis sought to determine if the Pt1 8.6 strain had undergone a process of “domestication.” However, unexpectedly, we did not observe differences in NS mutations. Instead, we observed a remarkable predominance of LOF mutations in the reference strain Pt1 8.6, suggesting an adaptation to laboratory culture conditions facilitated through LOF-mediated mechanisms. The majority of these LOF mutations resulted in the repression or reduced expression of targeted genes. However, a substantial number of genes showed moderate to high expression levels, implying that these LOF mutations may function as an evolutionary mechanism for generating new functional genes, serving as an adaptive response to culture conditions [63-65]. An illustrative example is the domestication of maize where most of the mutations are LOF and the selection for a variety of traits has led to fixation of LOF alleles in today’s crops [66, 67]. Another example is the human LOF mutations in the promoter of a red blood cell chemoreceptor, DARC, that resulted in the protection of human against malaria caused by *Plasmodium vivax* [68]. It is important to note that not all LOF mutations lead to complete functional knockout. For instance, LOF mutations at the 5′ or 3′ regions of genes may not entirely abolish their functions, and truncated proteins resulting from such mutations could act as dominant-negative factors [69, 70]. Interestingly, about 10.75% (331 out of 3078 genes) LOF showed moderate to high expression. Some specific examples of these genes with known functions include: (i) a mitochondrial enzyme known as glutamate dehydrogenase (Phatr3 J13951), which has been reported to play a crucial role in carbon and nitrogen metabolism as well as energy supply under abiotic stresses in *Arabidopsis* and the red alga *Pyropia haitanensis* [71–74]; (ii) an LCH15 protein (Phatr3 J48882), which functions as a chlorophyll-binding protein and potentially contributes to the adaptation to different light conditions in laboratory cultures; and (iii) a heat shock transcription factor (Phatr3 J49594) which similarly may contribute to the adaptation to lab culture temperatures.

Admixture analysis, PCA, and hierarchical clustering all identified six clusters, which suggests that the samples had shared ancestry and similar geographical origins. However, not all accessions within the same cluster had shared geographical origins. The English Channel and East China Sea populations clustered together, indicating that *P. tricornutum* accessions may have been dispersed by various means or that similar ecological niches across the sampling sites led to convergent evolution. Genome-wide phylogeny analysis confirmed six clades, with some accessions falling into previously identified clades and others forming two new clades, which suggests genetic divergence.

Several loci that are believed to be under BS were found to have a high level of genetic diversity. Notably, genes coding for stress-inducible proteins (Phatr3_EG00471, Phatr3_J54019), outer membrane receptors (Phatr3_EG01193), and cell cycle genes (Phatr3_J34920, Phatr3_EG00817) displayed an excess of polymorphism, reflecting their role in protecting cells from stresses such as high temperatures, starvation, and infection, as well as in cell division and growth. Consistent with this, transmembrane proteins have been previously observed to evolve faster than proteins without a transmembrane domain in unicellular eukaryotes such as yeast [75] and *Ostreococcus* [76]. Interestingly, the genes identified under BS were found in multiple clades and were specific to one or more accessions, suggesting that spatially varying selection forces may be related to local niches. It is expected that these same selection forces will apply to accessions from similar ecological niches or with a shared origin and sampling locations.

Comparison of genes under BS between accessions sampled at divergent time points, namely 1910, 1930, 1956, 1989, and 2016 revealed several identical genes suggesting the persistence of long-term BS acting on these genomic regions (Fig. 6A, Supplementary Table S8). Notably, the majority of these genes were functionally associated with stress resistance and fundamental cellular processes, highlighting their potential significance in adaptation to various habitats. Long-term BS was found at genes involved in diverse processes such as disease resistance, self-incompatibility, and heat stress providing advantages and enhancing fitness in natural populations [77–80].

Profiling transcript levels in the 17 accessions identified novel genes in the assayed growth conditions, suggesting condition- and accession-specific genes that were not identified in the numerous growth conditions previously reported [7]. Interestingly, our analysis revealed that genes carrying LOF mutations displayed a significant decrease in expression levels when compared to their non-LOF counterparts (Fig. 6B), implying the role of DNA sequence variations in shaping gene expression patterns. Nonetheless, we also noticed a considerable number of LOF mutations that did not result in downregulation. The observed LOF mutations with no effect on gene expression are likely due to the robustness of the genome through gene duplication and compensatory mechanisms allowing for the tolerance of many LOF variants, resulting in the majority of these mutations being silent and having little to no impact. Multiple LOF mutations were observed across clades as well as within them, particularly among accessions that exhibited extreme phenotypes (short cell size versus long ones, low versus high photosynthetic performance). This suggests that there may be variations in genetic backgrounds and/or epigenetic factors among these accessions. For instance, Pt12 and Pt14, despite having vastly different cell morphologies (very long versus short cells), share 1273 LOF mutations. Similarly, Pt2 and Pt9, as well as Pt6 and Pt12, share 1479 and 1120 LOF mutations, respectively, but exhibit distinct photosynthetic performances. In general, no clear association can be established between genetic diversity including SNP, INDELs, and LOF mutations and the phenotypic traits investigated in this study.

Interestingly, we observed LOF mutations that led to the upregulation of genes instead, suggesting the potential existence of gain-of-function (GOF) mutations. These GOF mutations known to occur mostly in unstructured regions may be attributed to the emergence of transcription factor binding sites, miRNA binding sites, an RNA binding protein, or new functional domains [81, 82]. Since genes and their products do not operate in isolation but rather in biological networks, these newly acquired domains may acquire functionality through their interactions with other proteins. Additionally, compensatory mechanisms for LOF or GOF may involve epigenetic processes that serve as a platform for interacting with specific proteins or complexes. WGCNA analysis revealed several network modules that were further merged into six clusters with similar expression patterns indicating co-regulated genes and pathways. Both differential expression and WGCNA analysis corroborated the presence of differences in transcript levels, which cannot be solely attributed to genetic variations. This observation implies the involvement of other regulatory mechanisms, such as epigenetics, that are known to modulate gene transcription [6, 83, 84].

## Conclusions

Our study provides a comprehensive assessment of the genetic and transcriptional diversity among 17 natural accessions of the model diatom *P. tricornutum*. Our investigation has uncovered novel clades, which are likely indicative of previously unexplored ecological niches. Moreover, we have identified new genes that expand the existing transcriptome repertoire of this species. By incorporating recently sampled accessions, we have further confirmed a persistent long-term BS and the high level of heterozygosity in *P. tricornutum* through population genetic analyses, suggesting that this characteristic arises from a heterozygous advantage. Our findings establish a crucial groundwork for future research that utilizes sequencing data from various *P. tricornutum* accessions which we made available via PhaeoEpiView platform (https://PhaeoEpiView.univ-nantes.fr) [10] for easy and comprehensive use. This will enhance our understanding of diatom biology, foster advancements in biotechnology applications, and optimize trait selection.

## Supplementary Material

Figure_S1_ycad008

Figure_S2_ycad008

Figure_S3_ycad008

Figure_S4_ycad008

Figure_S5_ycad008

Figure_S6_final_ycad008

Figure_S7_ycad008

Figure_S8_33_modules_ycad008

Table_S_all_ycad008

## Data Availability

The data that support the findings of this study are openly available in BioProjects PRJNA430316 and PRJNA971163.
