# Supplementary material for: Genome-wide assessment of genetic diversity and transcript variations in 17 accessions of the model diatom Phaeodactylum tricornutum
Source: ISME Commun. 2024 Jan 10;4(1):ycad008. doi: 10.1093/ismeco/ycad008 (PMC10833087; doi:10.1093/ismeco/ycad008)

a
Pt3

Totally number: 248

Oval shaped: 18

Percentage of oval: 7.3%

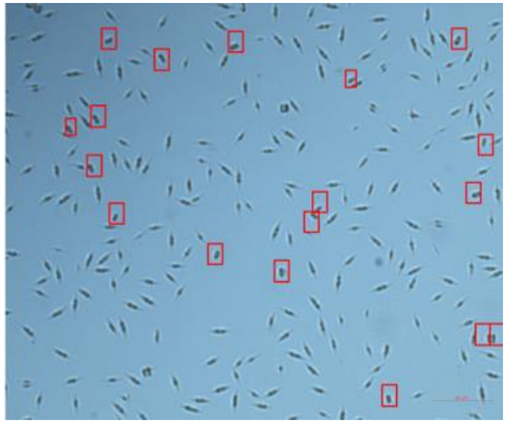

b
Pt9

Totally number: 105

Oval shaped: 8

Percentage of oval: 7.6%

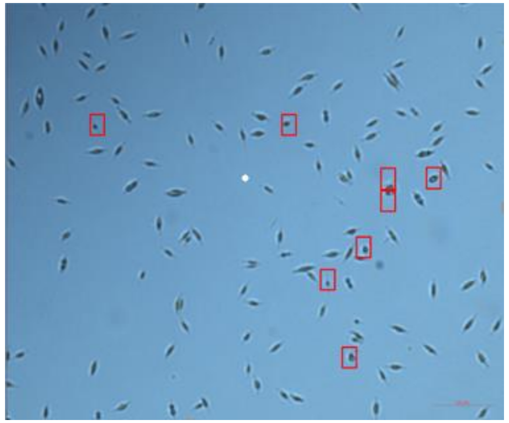

c
Pt11

Length: 12-16 µm
Average: 14.4 µm

Width: 2.2-3.2 µm
Average: 2.7 µm

Average values of
Length/width:
5.35 ± 0.7

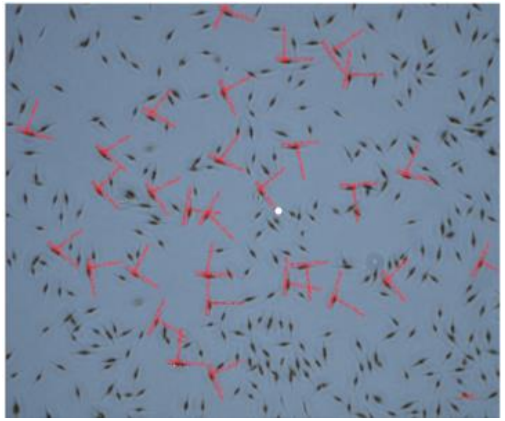

d
Pt12

Length: 19-22 µm
Average: 21 µm

Width: 1.8-2.4 µm
Average: 2.7 µm

Average values of
Length/width:
9.9 ± 1.0

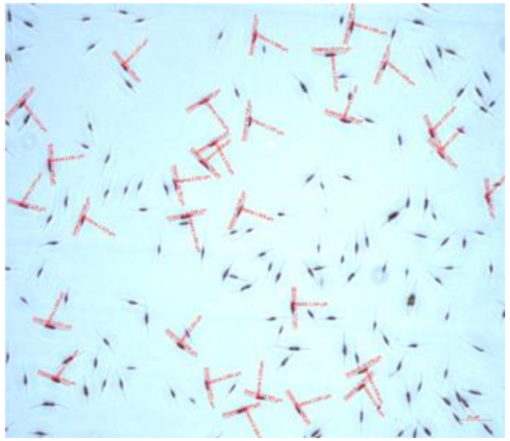

e
Pt13

Length: 16-21 µm
Average: 18.6 µm

Width: 1.8-3.0 µm
Average: 2.4 µm

Average values of
Length/width:
7.8 ± 1.1

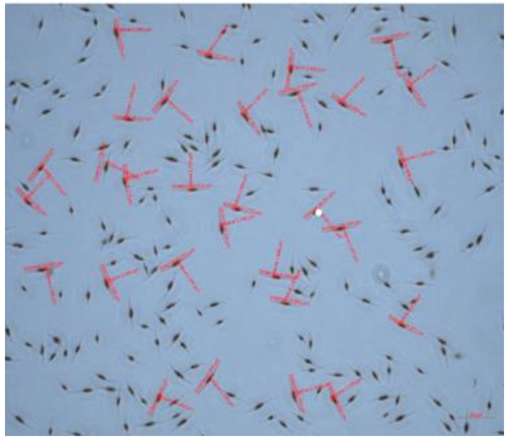

f
Pt14

Length: 10-15 µm
Average: 12.4 µm

Width: 2.3-3.2 µm
Average: 2.6 µm

Average values of
Length/width:
4.8 ± 0.5

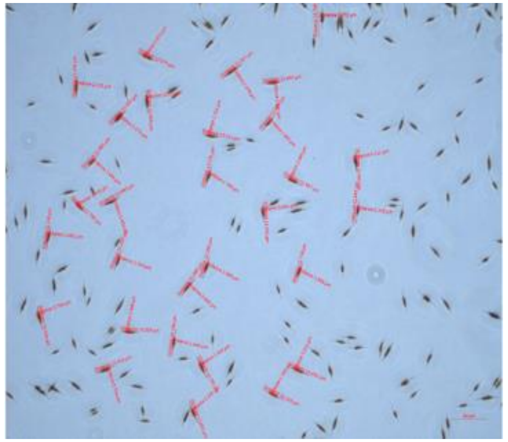

g
Pt15

Length: 11-17 µm
Average: 14.1 µm

Width: 2.2-3.1 µm
Average: 2.6 µm

Average values of
Length/width:
5.5 ± 0.6

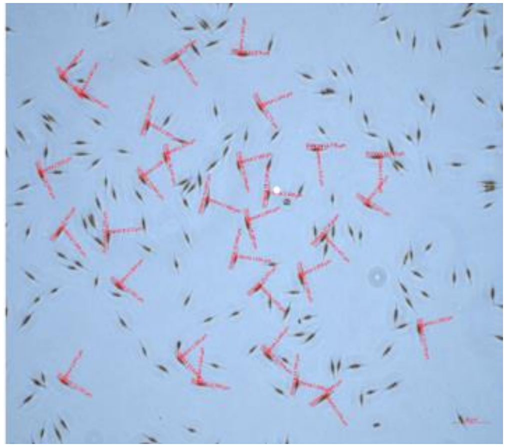

h
Pt16

Length: 12-18 µm
Average: 15.4 µm

Width: 1.9-3.2 µm
Average: 2.4 µm

Average values of
Length/width:
6.3 ± 0.8

Percentage of Triradiate
shape: 25.2%

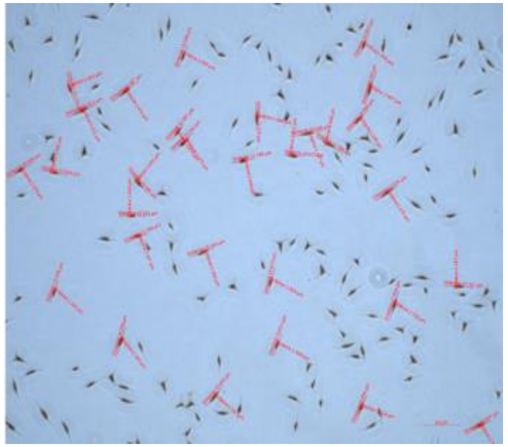

i
Pt17

Length: 10-16 µm
Average: 12.1 µm

Width: 2.4-3.0 µm
Average: 2.6 µm

Average values of
Length/width:
4.6 ± 0.8

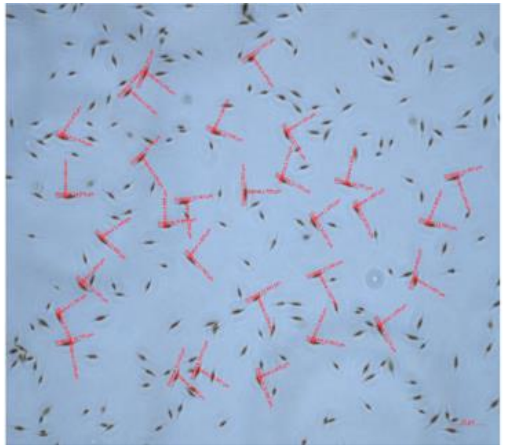

Supplement: Figure_S1_ycad008 [file figure_s1_ycad008.pdf]
