# Supplementary material for: Genome-wide assessment of genetic diversity and transcript variations in 17 accessions of the model diatom Phaeodactylum tricornutum
Source: ISME Commun. 2024 Jan 10;4(1):ycad008. doi: 10.1093/ismeco/ycad008 (PMC10833087; doi:10.1093/ismeco/ycad008)
Supplement: Figure_S2_ycad008 [file figure_s2_ycad008.pdf]

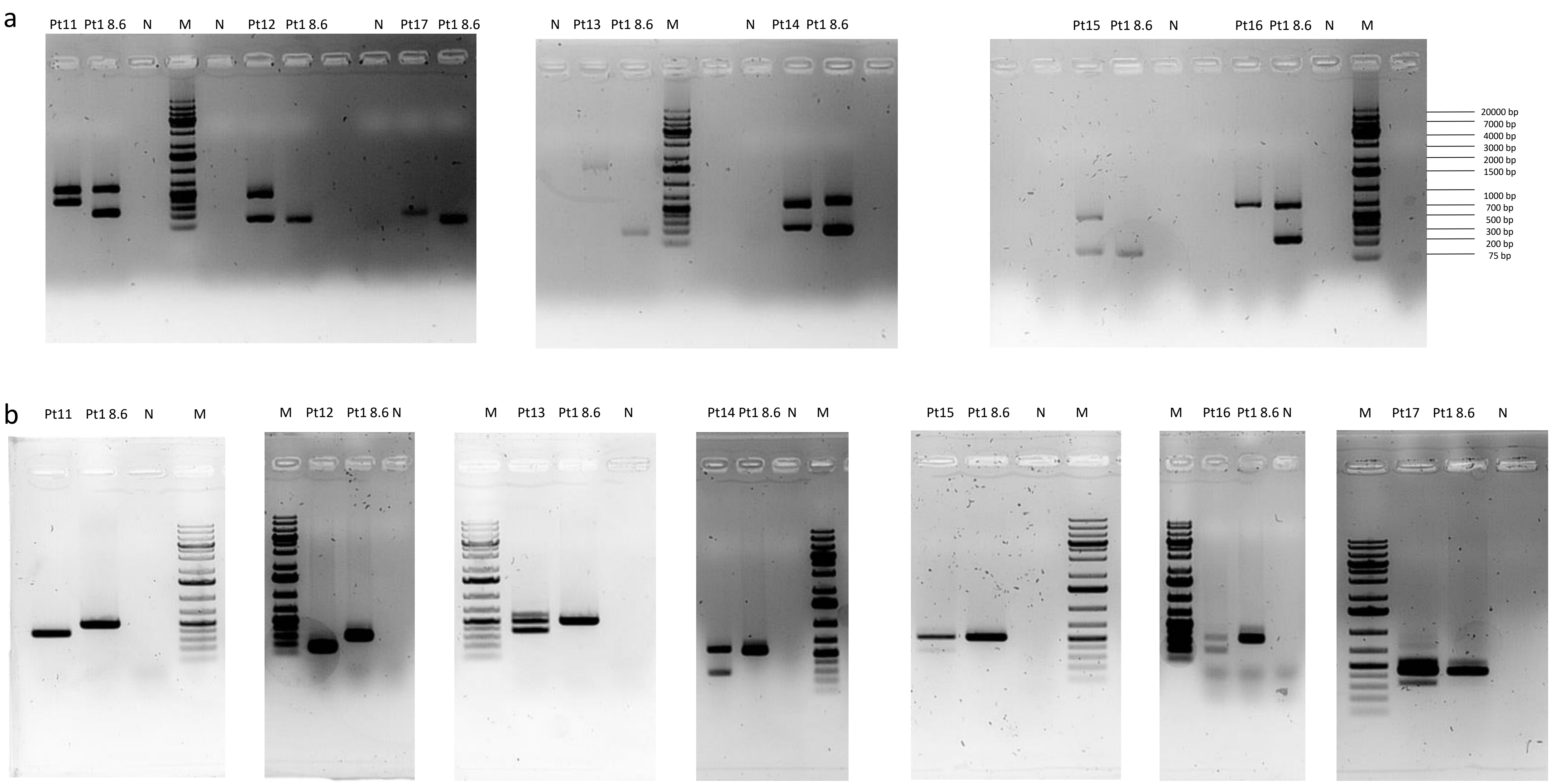
a
Pt11 Pt1 8.6 N M N Pt12 Pt1 8.6 N Pt17 Pt1 8.6
N Pt13 Pt1 8.6 M N Pt14 Pt1 8.6
Pt15 Pt1 8.6 N Pt16 Pt1 8.6 N M
20000 bp
7000 bp
4000 bp
3000 bp
2000 bp
1500 bp
1000 bp
700 bp
500 bp
300 bp
200 bp
75 bp
b
Pt11 Pt1 8.6 N M
M Pt12 Pt1 8.6 N
M Pt13 Pt1 8.6 N
Pt14 Pt1 8.6 N M
Pt15 Pt1 8.6 N M
M Pt16 Pt1 8.6 N
M Pt17 Pt1 8.6 N
