# Supplementary material for: Genome-wide assessment of genetic diversity and transcript variations in 17 accessions of the model diatom Phaeodactylum tricornutum
Source: ISME Commun. 2024 Jan 10;4(1):ycad008. doi: 10.1093/ismeco/ycad008 (PMC10833087; doi:10.1093/ismeco/ycad008)
Supplement: Figure_S3_ycad008 [file figure_s3_ycad008.pdf]

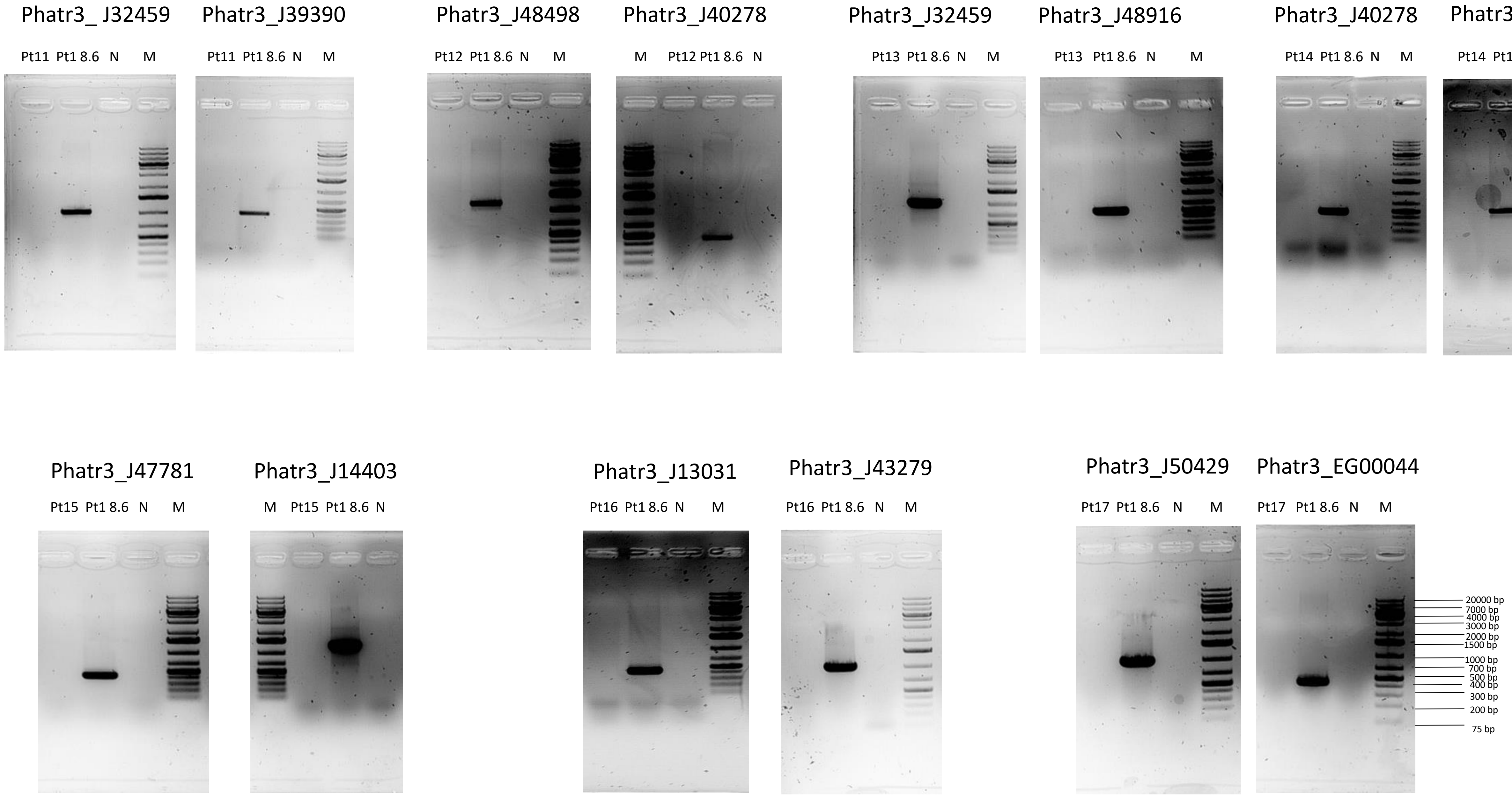
Phatr3_ J32459
Pt11 Pt1 8.6 N M
Phatr3_J39390
Pt11 Pt1 8.6 N M
Phatr3_J48498
Pt12 Pt1 8.6 N M
Phatr3_J40278
M Pt12 Pt1 8.6 N
Phatr3_J32459
Pt13 Pt1 8.6 N M
Phatr3_J48916
Pt13 Pt1 8.6 N M
Phatr3_J40278
Pt14 Pt1 8.6 N M
Phatr3_J44991
Pt14 Pt1 8.6 N M
Phatr3_J47781
Pt15 Pt1 8.6 N M
Phatr3_J14403
M Pt15 Pt1 8.6 N
Phatr3_J13031
Pt16 Pt1 8.6 N M
Phatr3_J43279
Pt16 Pt1 8.6 N M
Phatr3_J50429
Pt17 Pt1 8.6 N M
Phatr3_EG00044
Pt17 Pt1 8.6 N M
20000 bp
7000 bp
4000 bp
3000 bp
2000 bp
1500 bp
1000 bp
700 bp
500 bp
400 bp
300 bp
200 bp
75 bp
