# Supplementary figures and images for: Genome-wide assessment of genetic diversity and transcript variations in 17 accessions of the model diatom Phaeodactylum tricornutum
Source: ISME Commun. 2024 Jan 10;4(1):ycad008. doi: 10.1093/ismeco/ycad008 (PMC10833087; doi:10.1093/ismeco/ycad008)

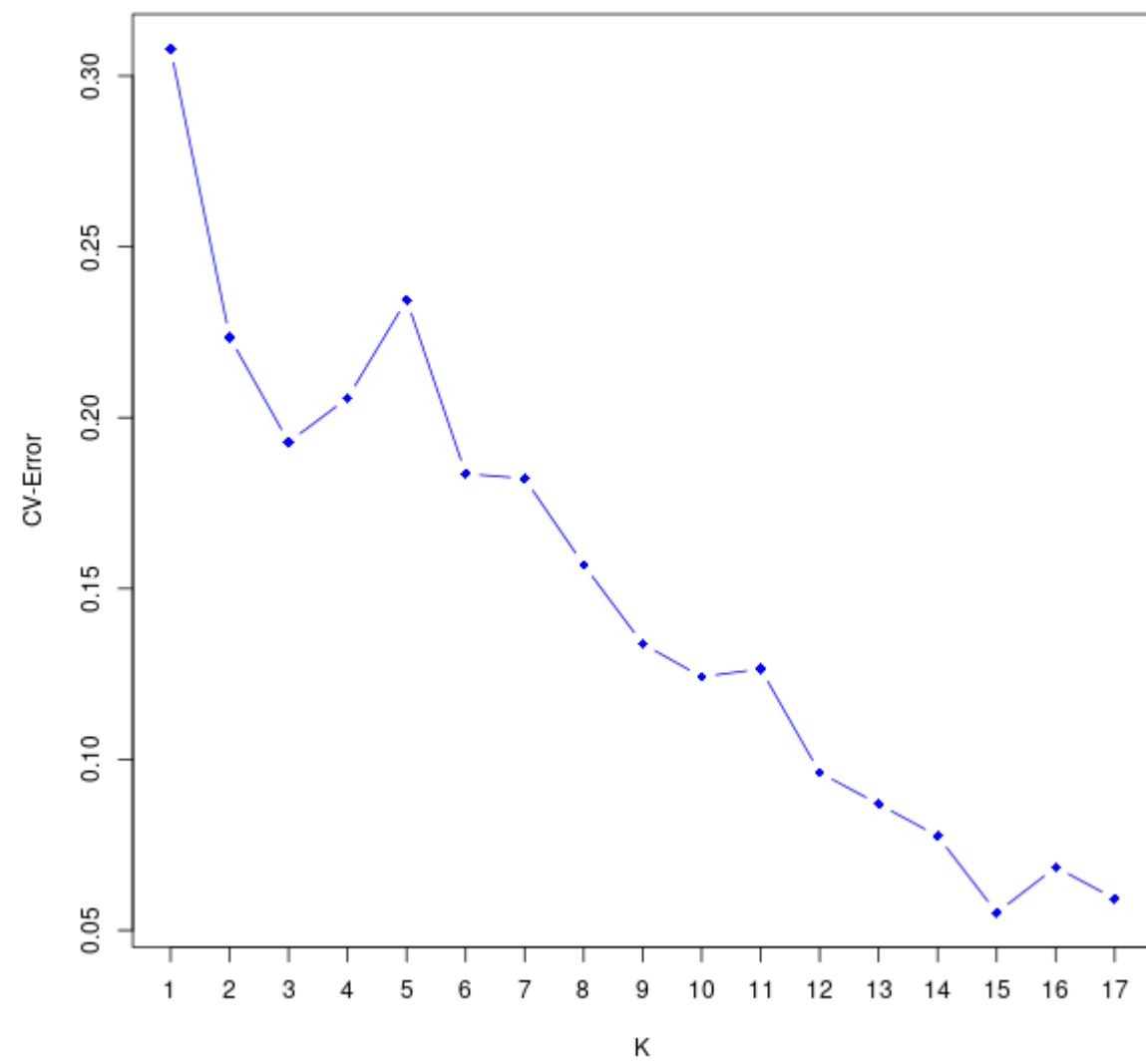

Supplement: Figure_S4_ycad008 [file figure_s4_ycad008.pdf]

## Pearson correlation between samples

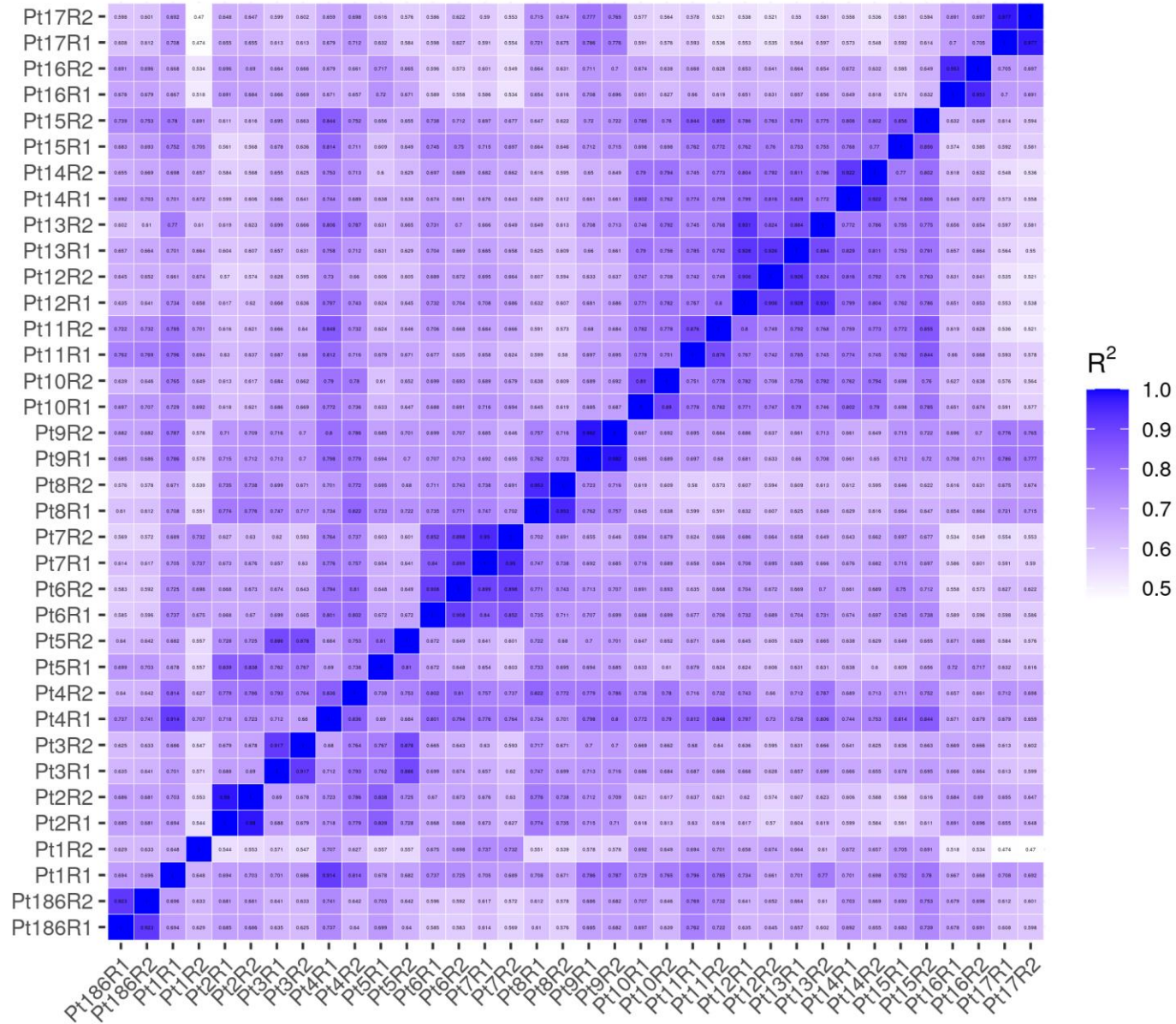

Supplement: Figure_S5_ycad008 [file figure_s5_ycad008.pdf]

a

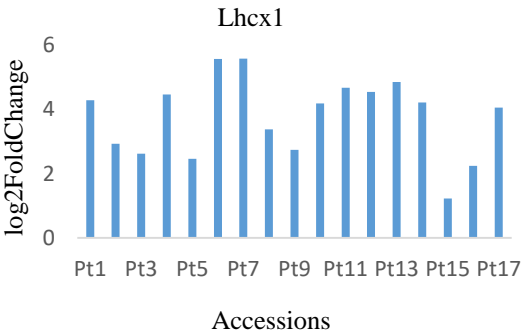

e

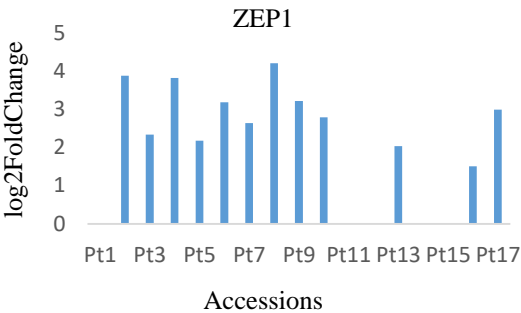

b

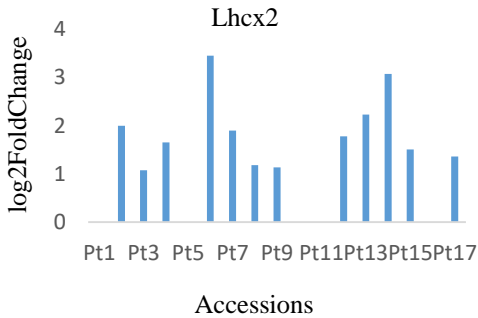

f

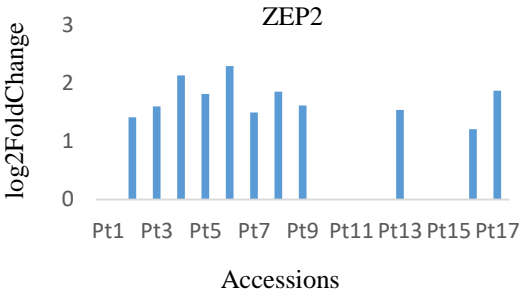

c

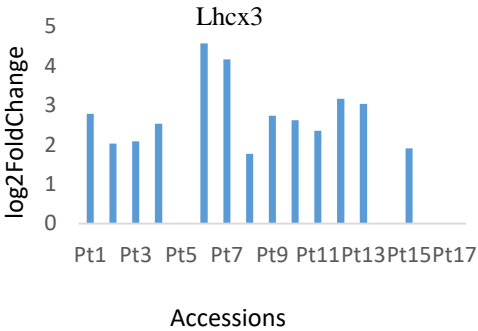

g

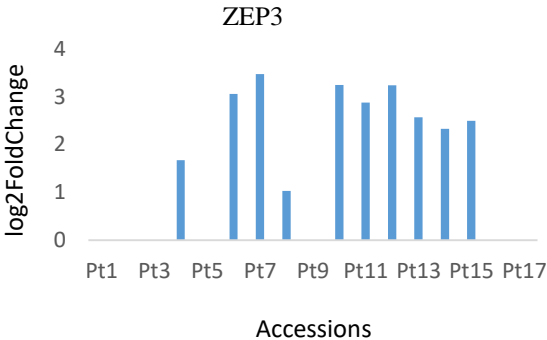

d

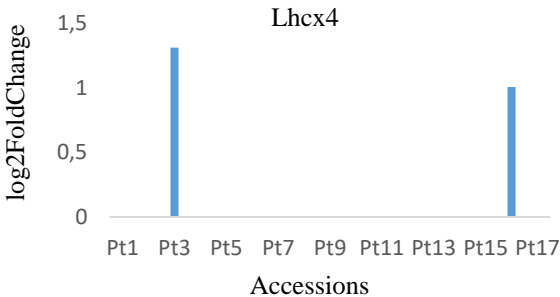

Supplement: Figure_S6_final_ycad008 [file figure_s6_final_ycad008.pdf]

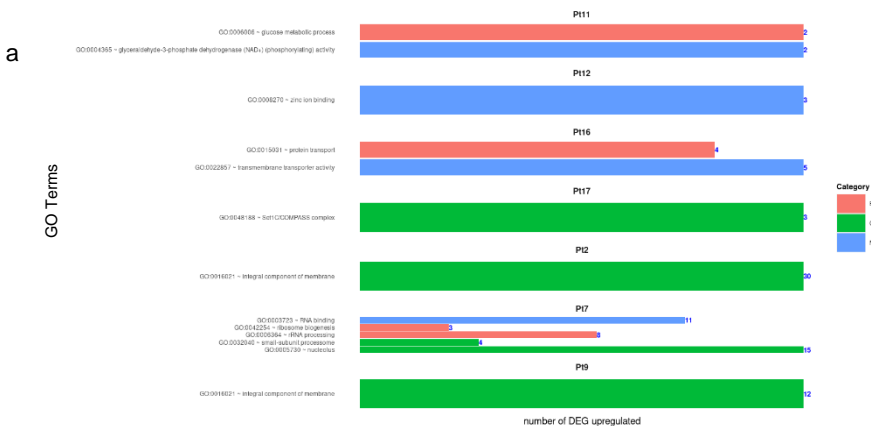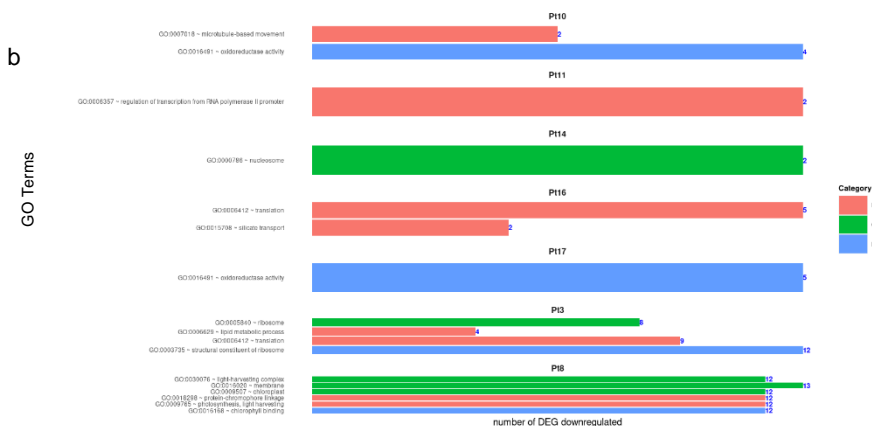

Supplement: Figure_S7_ycad008 [file figure_s7_ycad008.pdf]
