# Supplementary material for: Genome-wide assessment of genetic diversity and transcript variations in 17 accessions of the model diatom Phaeodactylum tricornutum
Source: ISME Commun. 2024 Jan 10;4(1):ycad008. doi: 10.1093/ismeco/ycad008 (PMC10833087; doi:10.1093/ismeco/ycad008)
Supplement: Figure_S8_33_modules_ycad008 [file figure_s8_33_modules_ycad008.pdf]

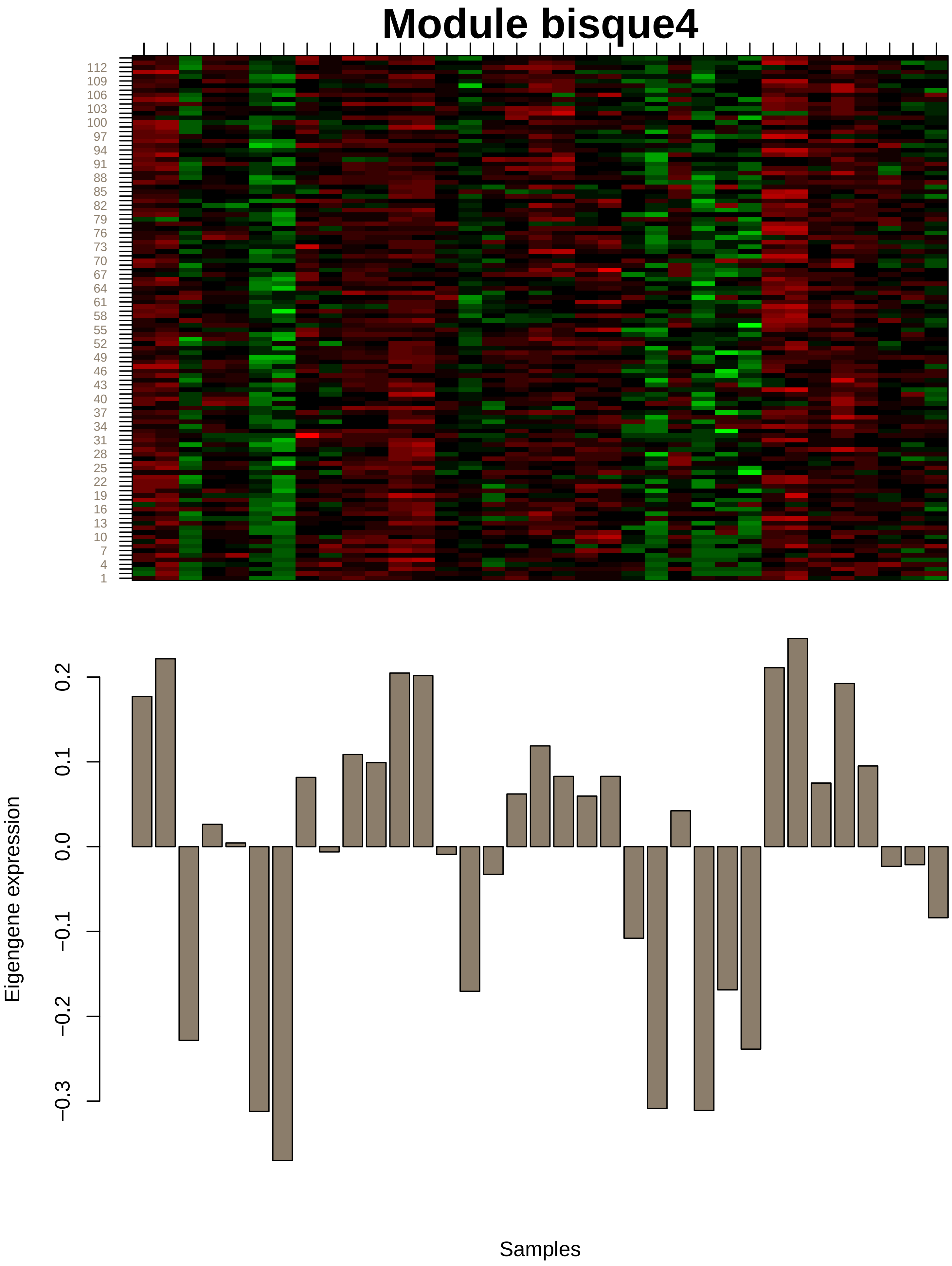
Module bisque4
112
109
106
103
100
97
94
91
88
85
82
79
76
73
70
67
64
61
58
55
52
49
46
43
40
37
34
31
28
25
22
19
16
13
10
7
4
1
0.2
0.1
0.0
−0.1
−0.2
−0.3
Eigengene expression
Samples

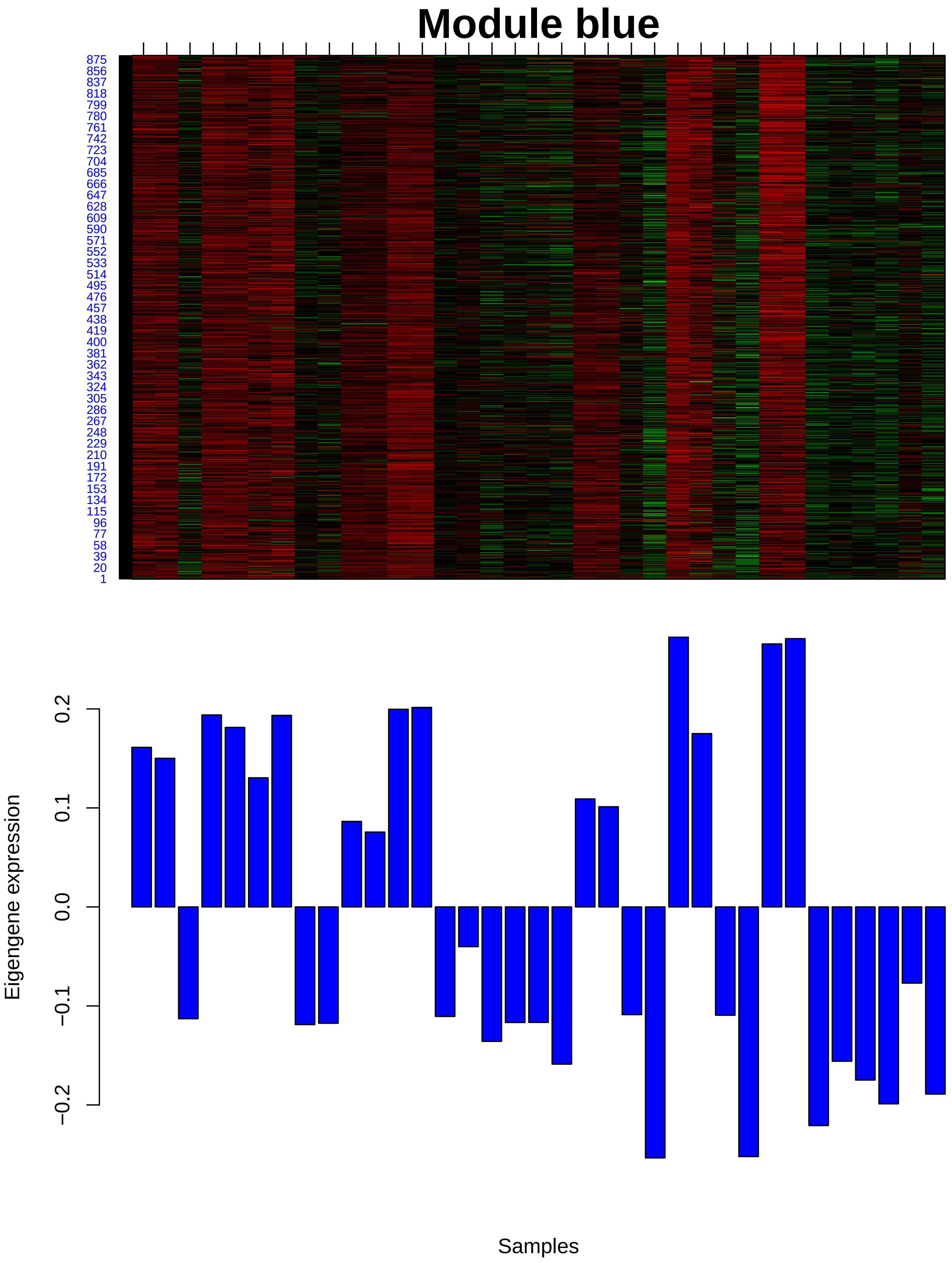
Module blue
875
856
837
818
799
780
761
742
723
704
685
666
647
628
609
590
571
552
533
514
495
476
457
438
419
400
381
362
343
324
305
286
267
248
229
210
191
172
153
134
115
96
77
58
39
20
1
0.2
0.1
0.0
−0.1
−0.2
Eigengene expression
Samples

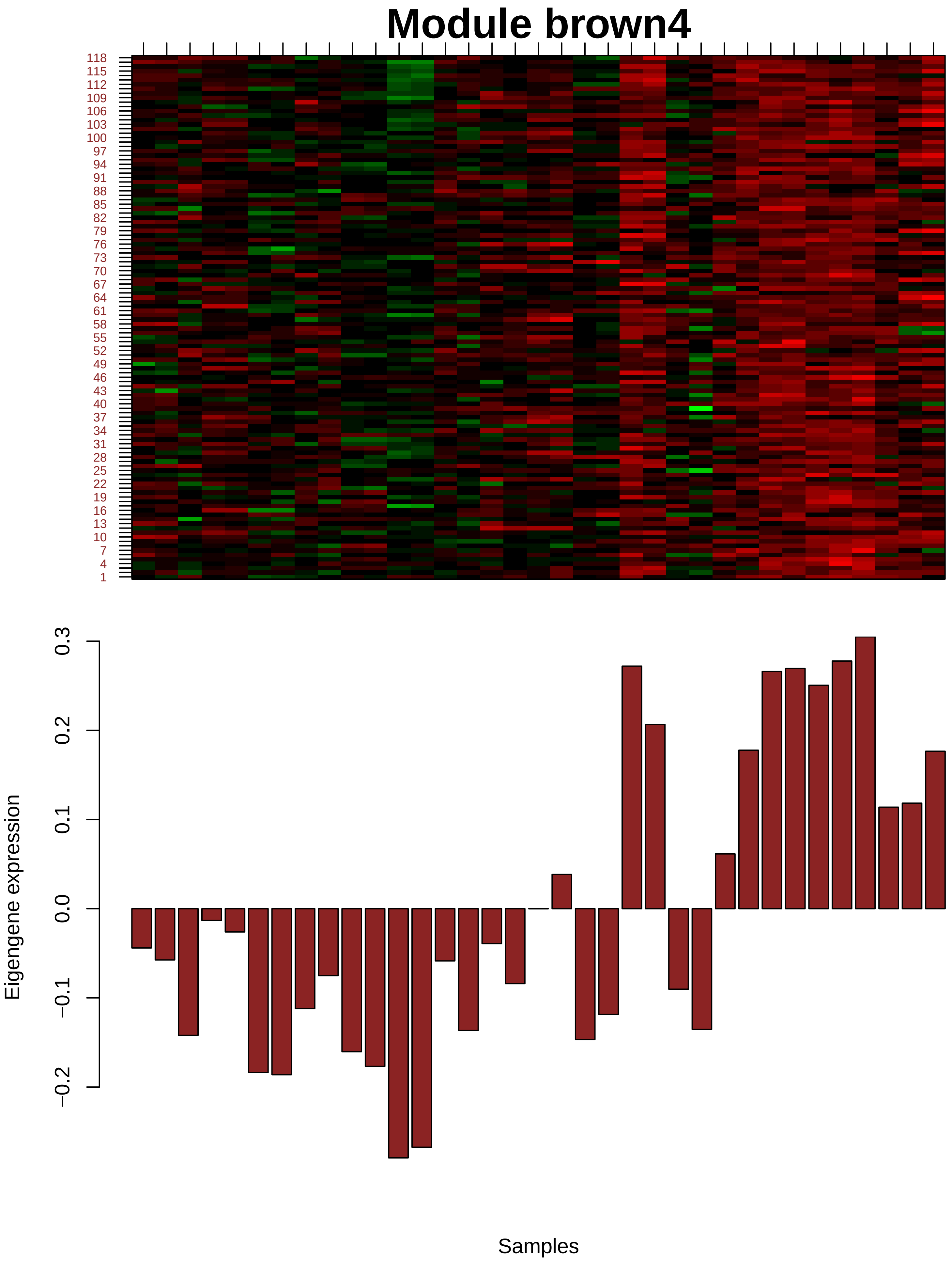
Module brown4
118
115
112
109
106
103
100
97
94
91
88
85
82
79
76
73
70
67
64
61
58
55
52
49
46
43
40
37
34
31
28
25
22
19
16
13
10
7
4
1
Eigengene expression
0.3
0.2
0.1
0.0
−0.1
−0.2
Samples

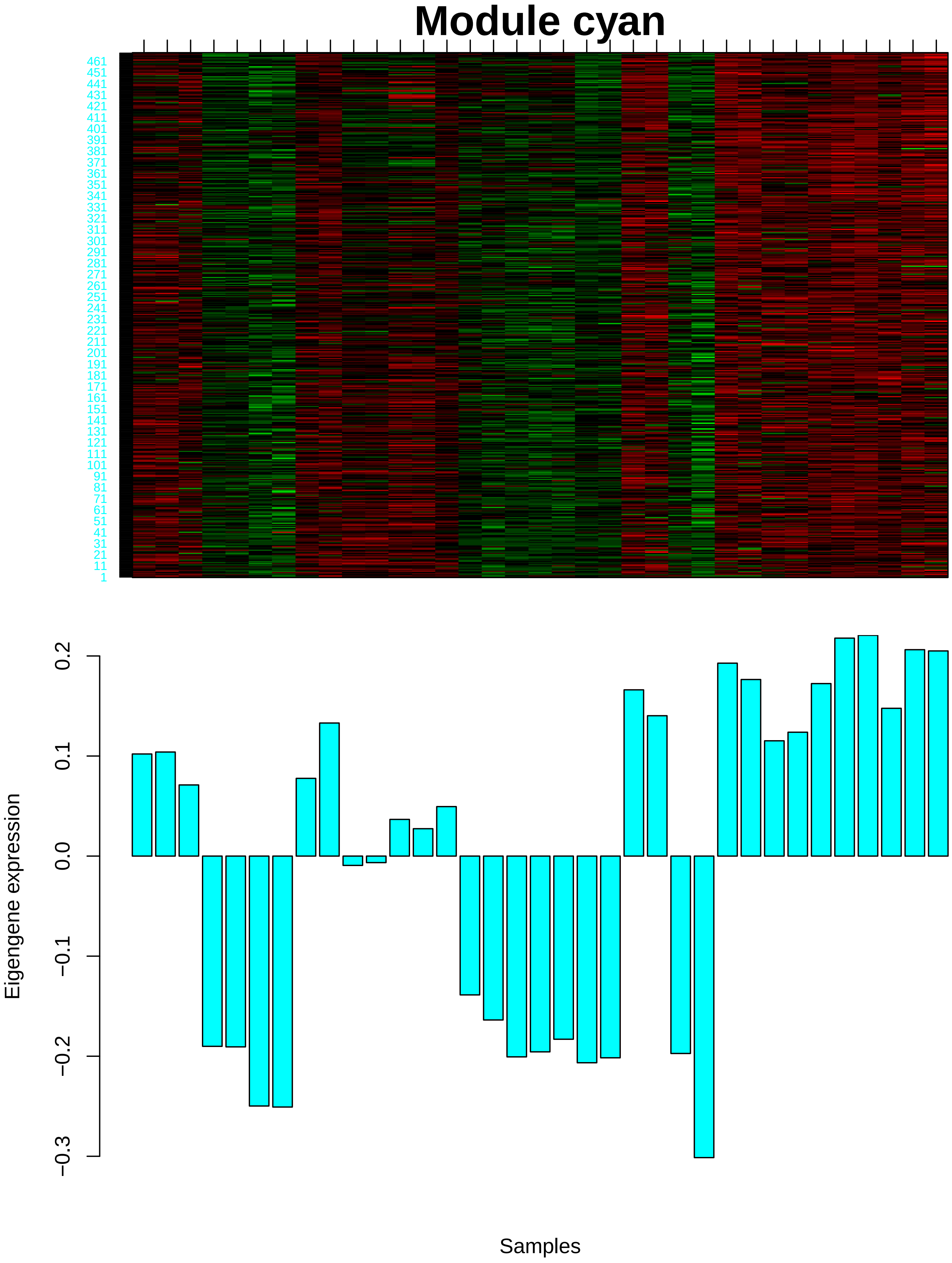

Module cyan
461
451
441
431
421
411
401
391
381
371
361
351
341
331
321
311
301
291
281
271
261
251
241
231
221
211
201
191
181
171
161
151
141
131
121
111
101
91
81
71
61
51
41
31
21
11
1
Eigengene expression
0.2
0.1
0.0
−0.1
−0.2
−0.3
Samples

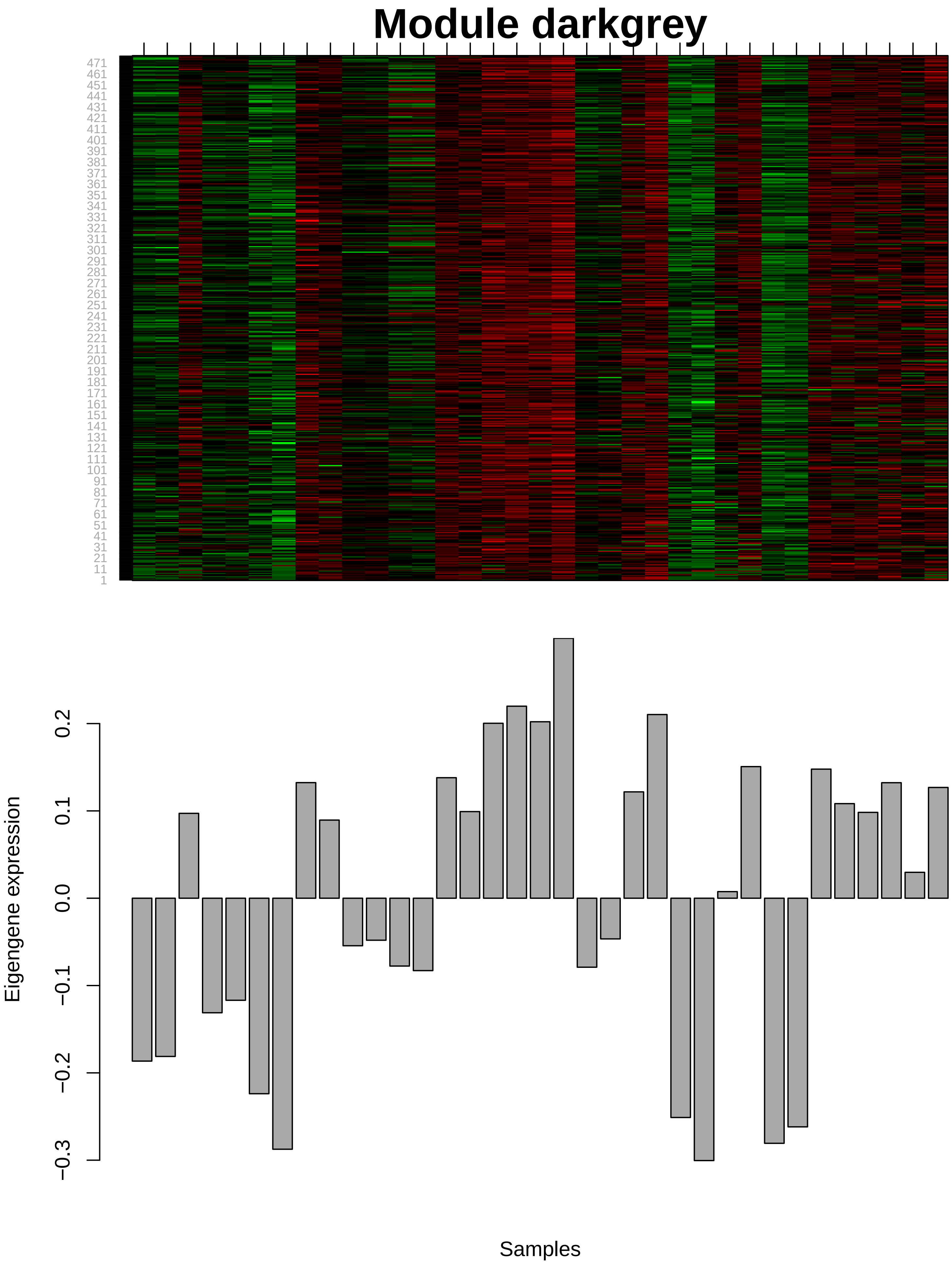
Module darkgrey
471
461
451
441
431
421
411
401
391
381
371
361
351
341
331
321
311
301
291
281
271
261
251
241
231
221
211
201
191
181
171
161
151
141
131
121
111
101
91
81
71
61
51
41
31
21
11
1
Eigengene expression
0.2
0.1
0.0
−0.1
−0.2
−0.3
Samples

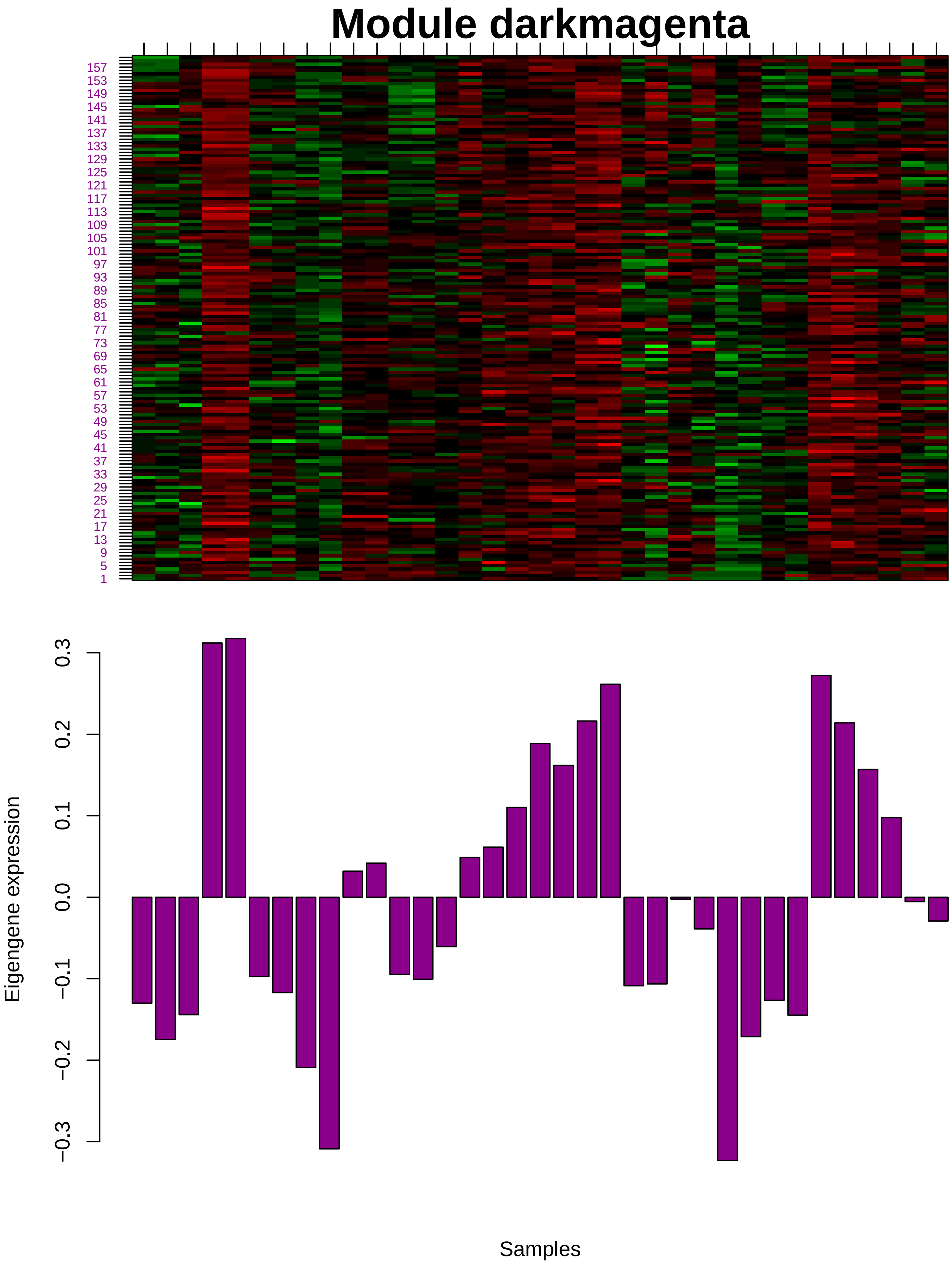
Module darkmagenta
157
153
149
145
141
137
133
129
125
121
117
113
109
105
101
97
93
89
85
81
77
73
69
65
61
57
53
49
45
41
37
33
29
25
21
17
13
9
5
1
0.3
0.2
0.1
0.0
−0.1
−0.2
−0.3
Eigengene expression
Samples

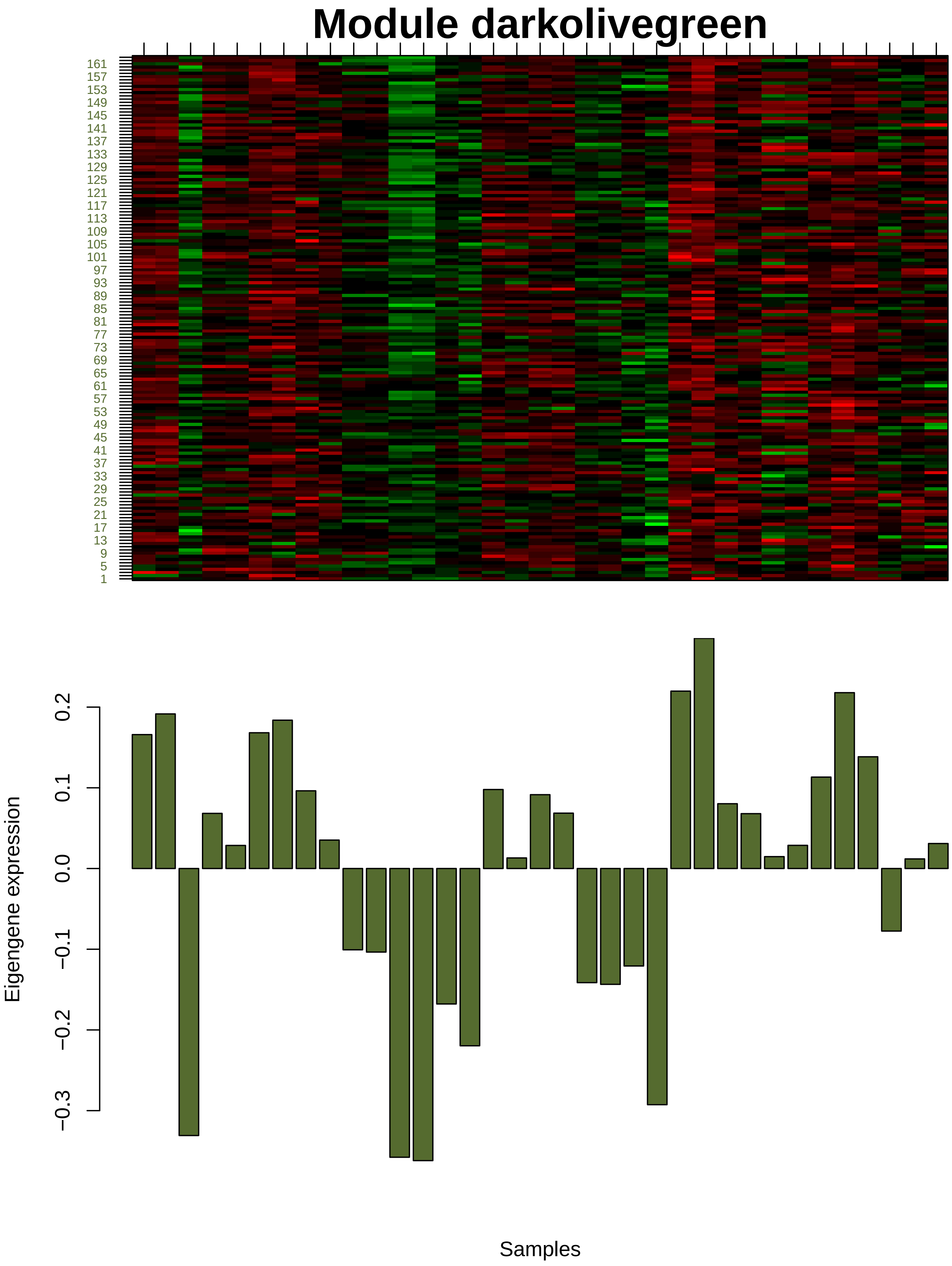

Module darkolivegreen
161
157
153
149
145
141
137
133
129
125
121
117
113
109
105
101
97
93
89
85
81
77
73
69
65
61
57
53
49
45
41
37
33
29
25
21
17
13
9
5
1
Eigengene expression
0.2
0.1
0.0
−0.1
−0.2
−0.3
Samples

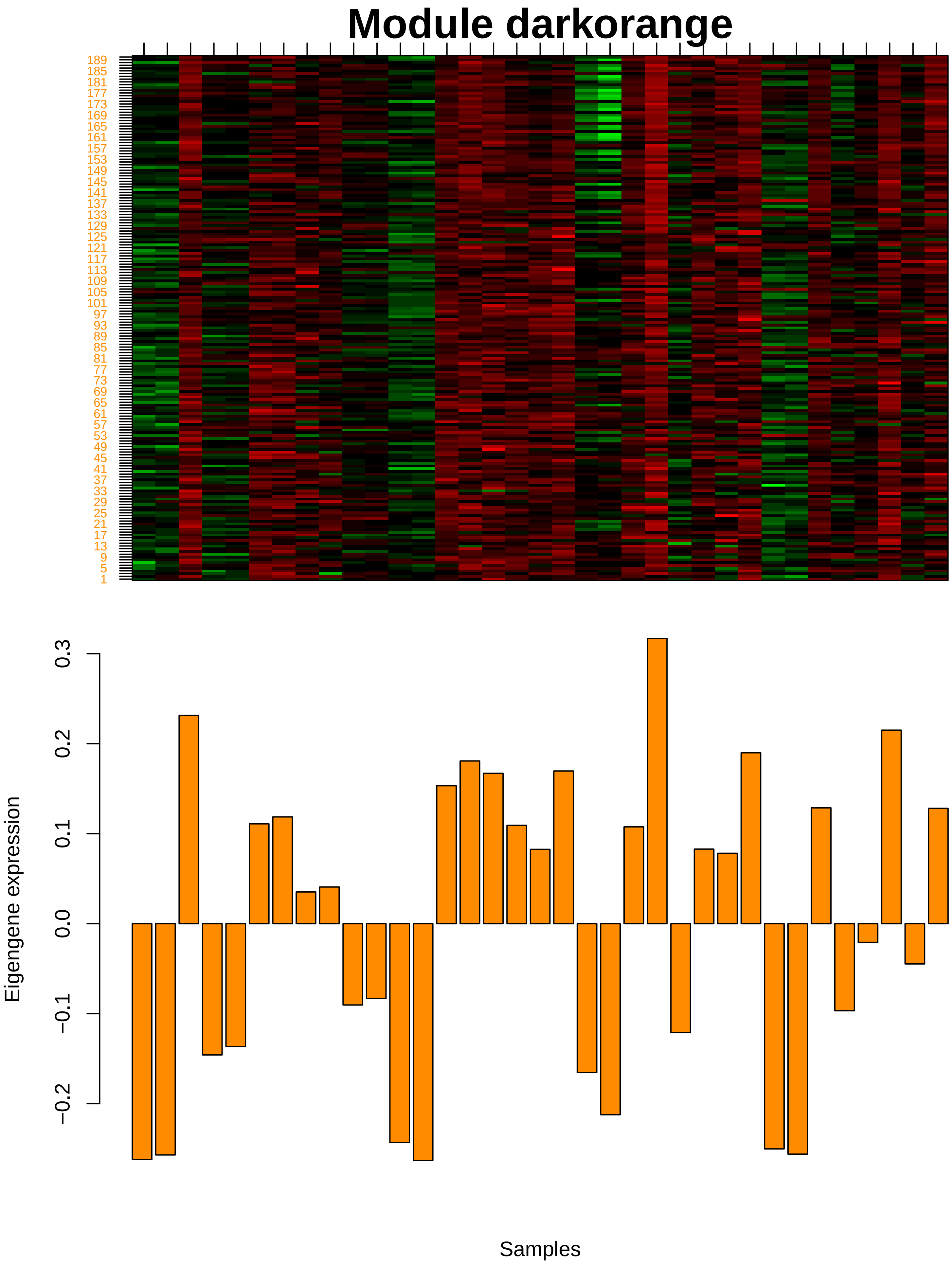

Module darkorange
Eigengene expression
Samples

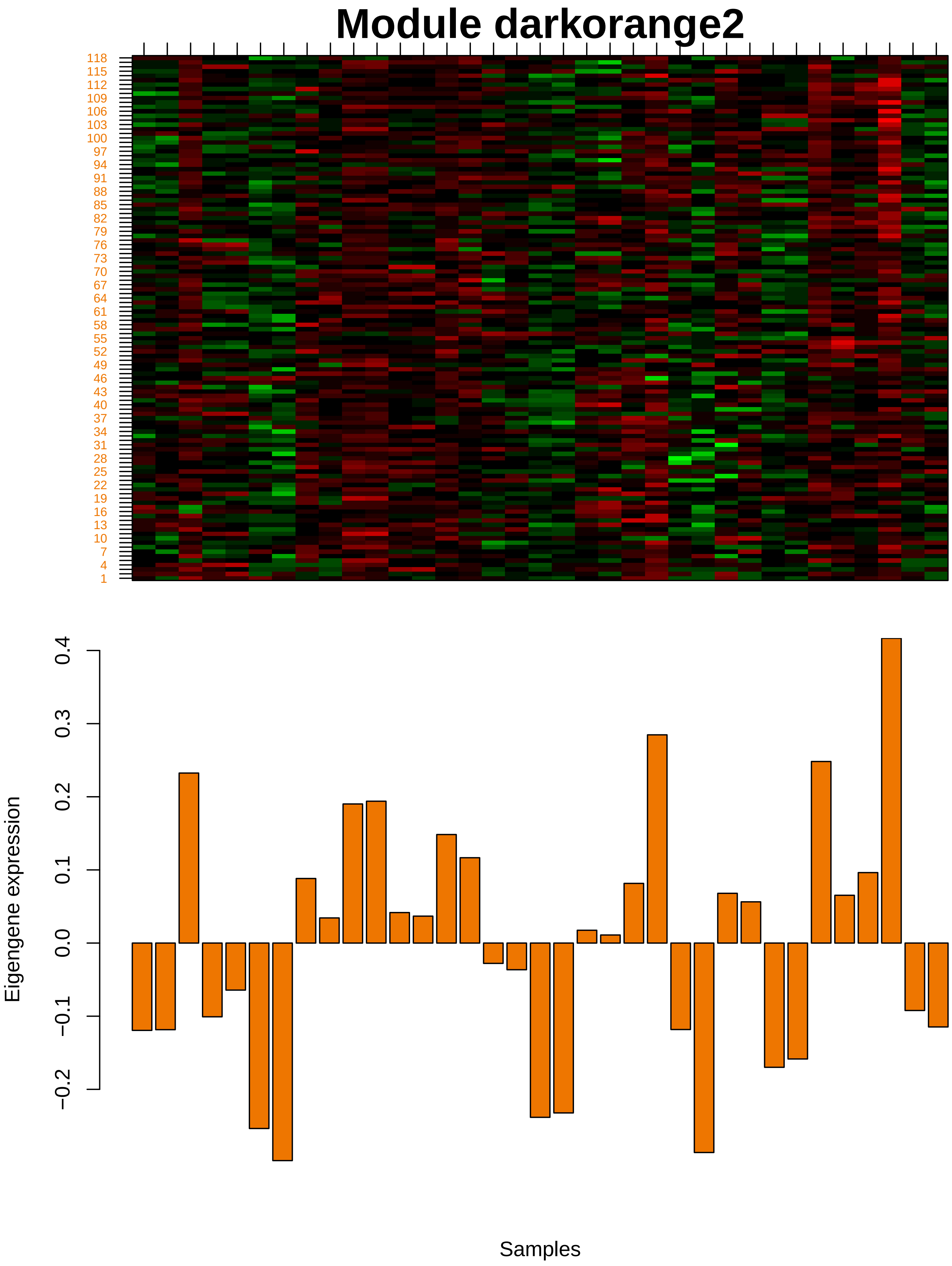

Module darkorange2
118
115
112
109
106
103
100
97
94
91
88
85
82
79
76
73
70
67
64
61
58
55
52
49
46
43
40
37
34
31
28
25
22
19
16
13
10
7
4
1
Eigengene expression
0.4
0.3
0.2
0.1
0.0
−0.1
−0.2
Samples

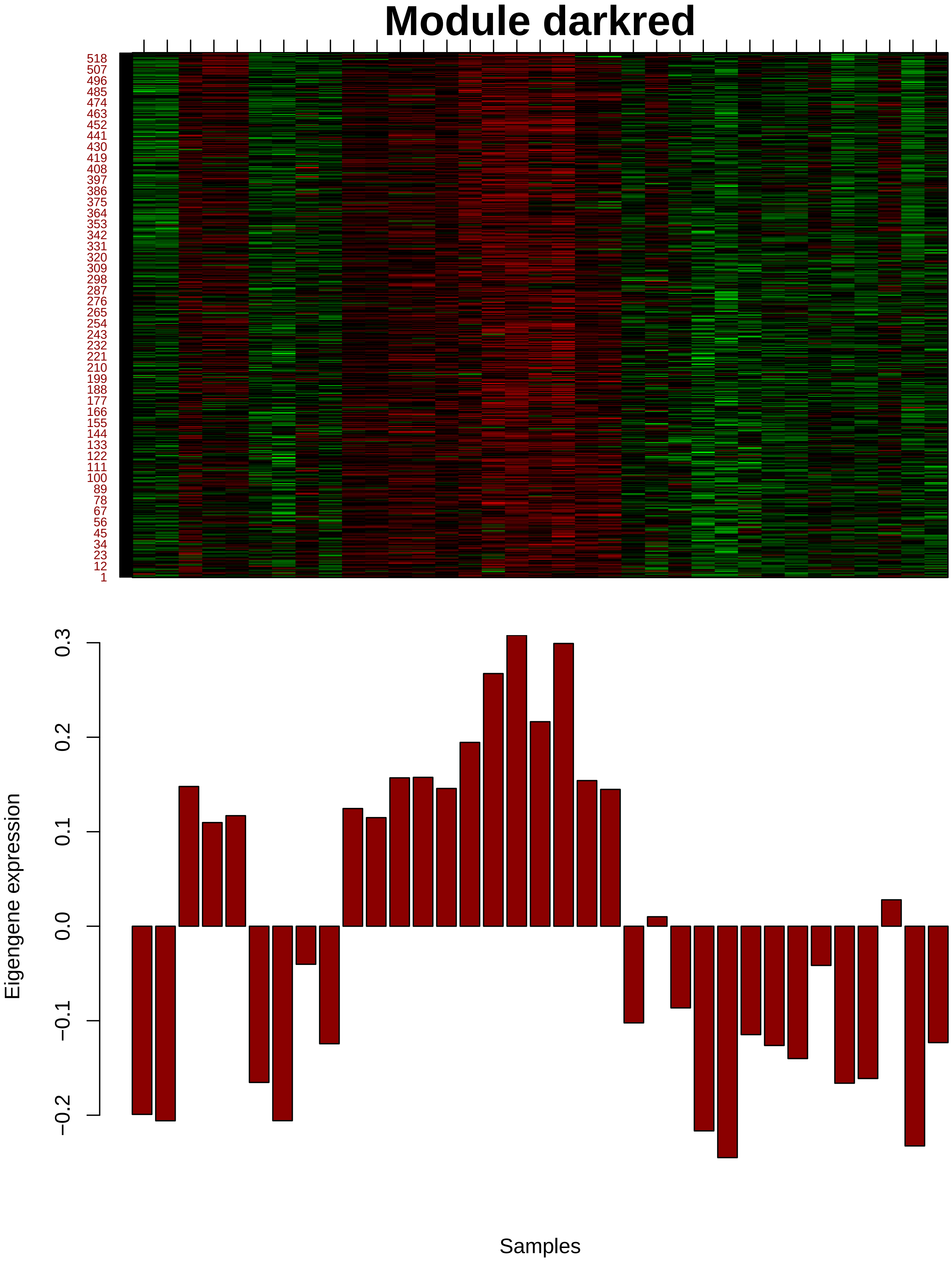

Module darkred
518
507
496
485
474
463
452
441
430
419
408
397
386
375
364
353
342
331
320
309
298
287
276
265
254
243
232
221
210
199
188
177
166
155
144
133
122
111
100
89
78
67
56
45
34
23
12
1
Eigengene expression
0.3
0.2
0.1
0.0
−0.1
−0.2
Samples

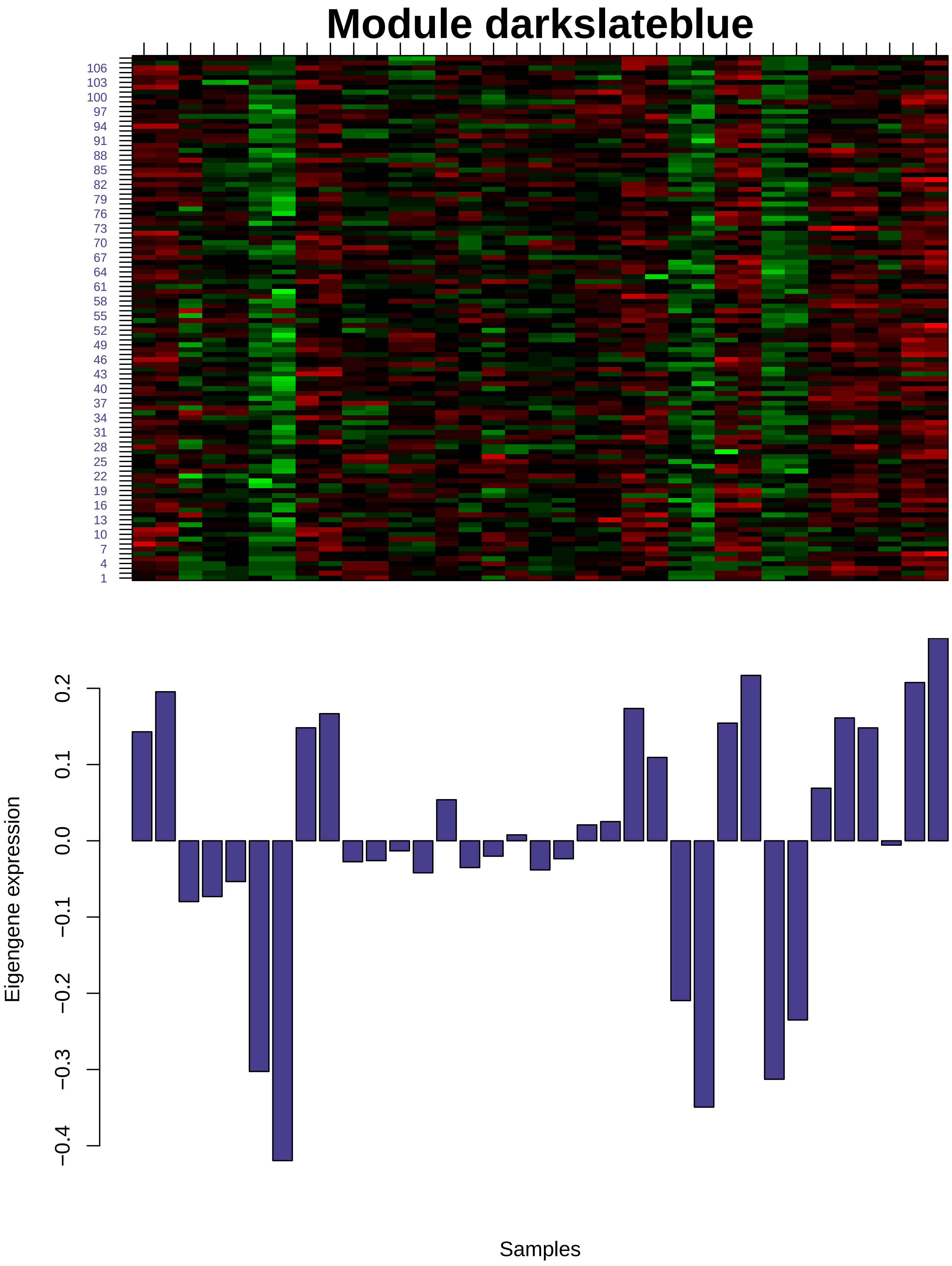

Module darkslateblue
106
103
100
97
94
91
88
85
82
79
76
73
70
67
64
61
58
55
52
49
46
43
40
37
34
31
28
25
22
19
16
13
10
7
4
1
Eigengene expression
0.2
0.1
0.0
−0.1
−0.2
−0.3
−0.4
Samples

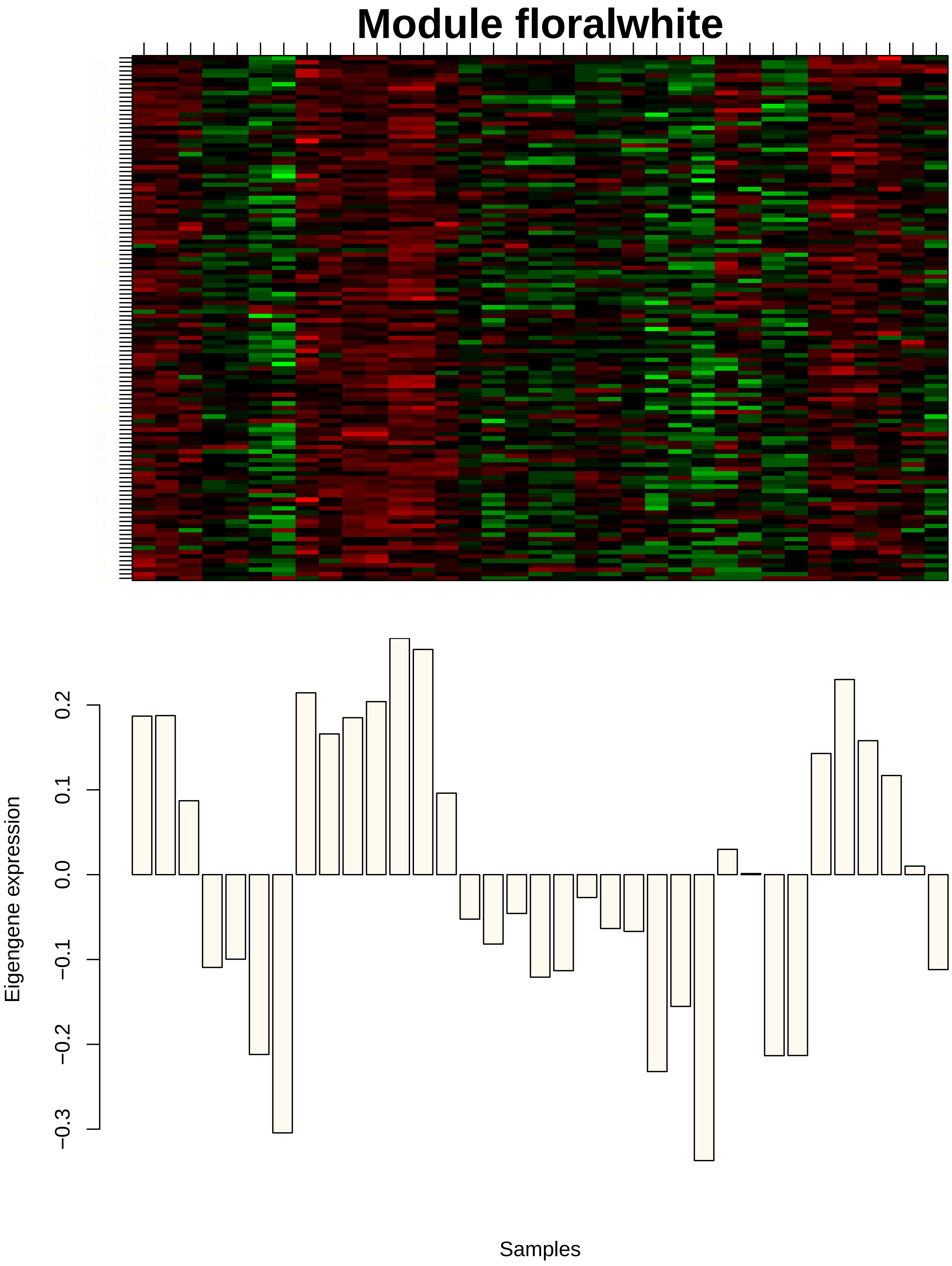

Module floralwhite
Eigengene expression
0.2
0.1
0.0
−0.1
−0.2
−0.3
Samples

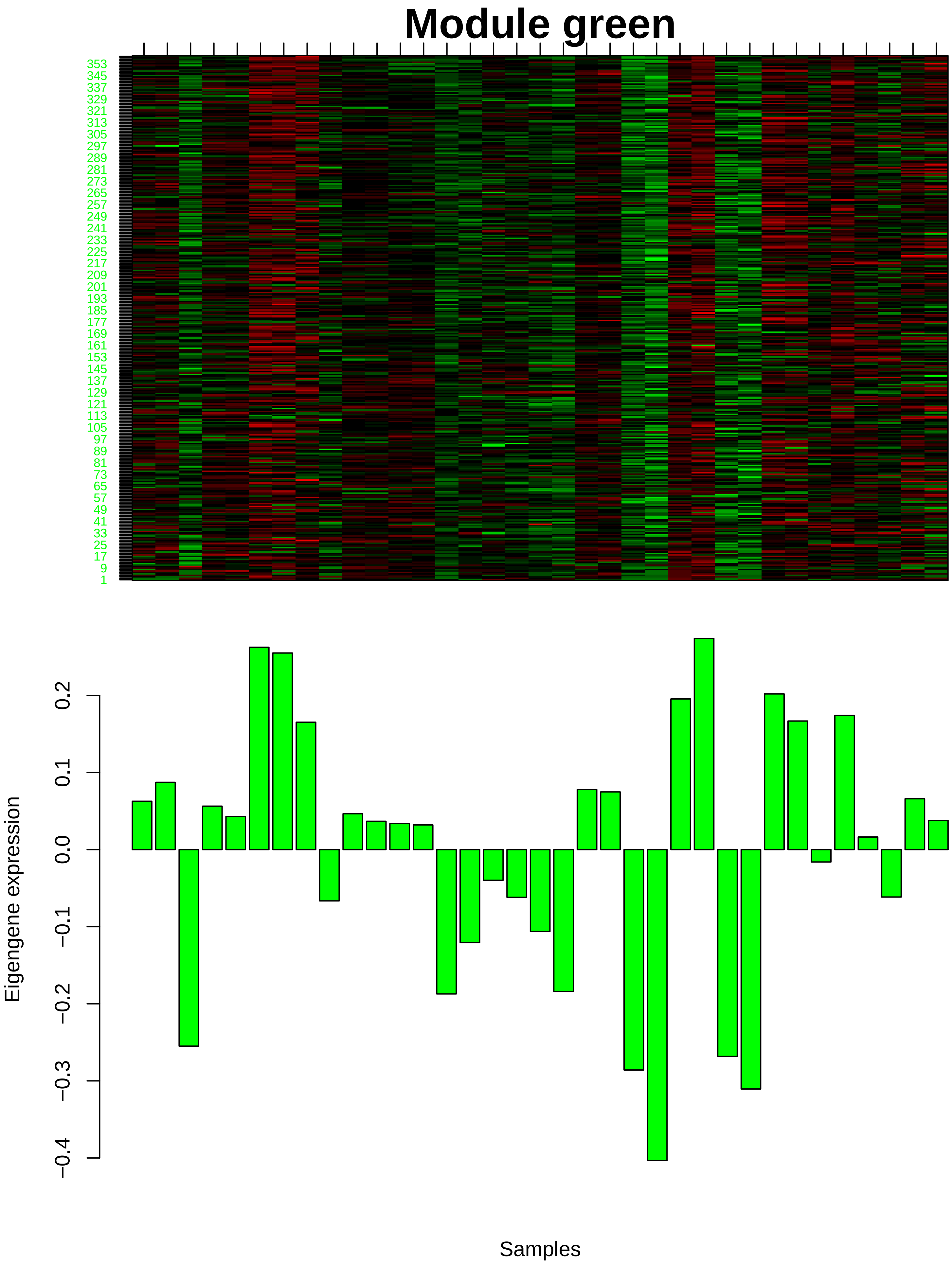
Module green
Eigengene expression
Samples
0.2
0.1
0.0
−0.1
−0.2
−0.3
−0.4

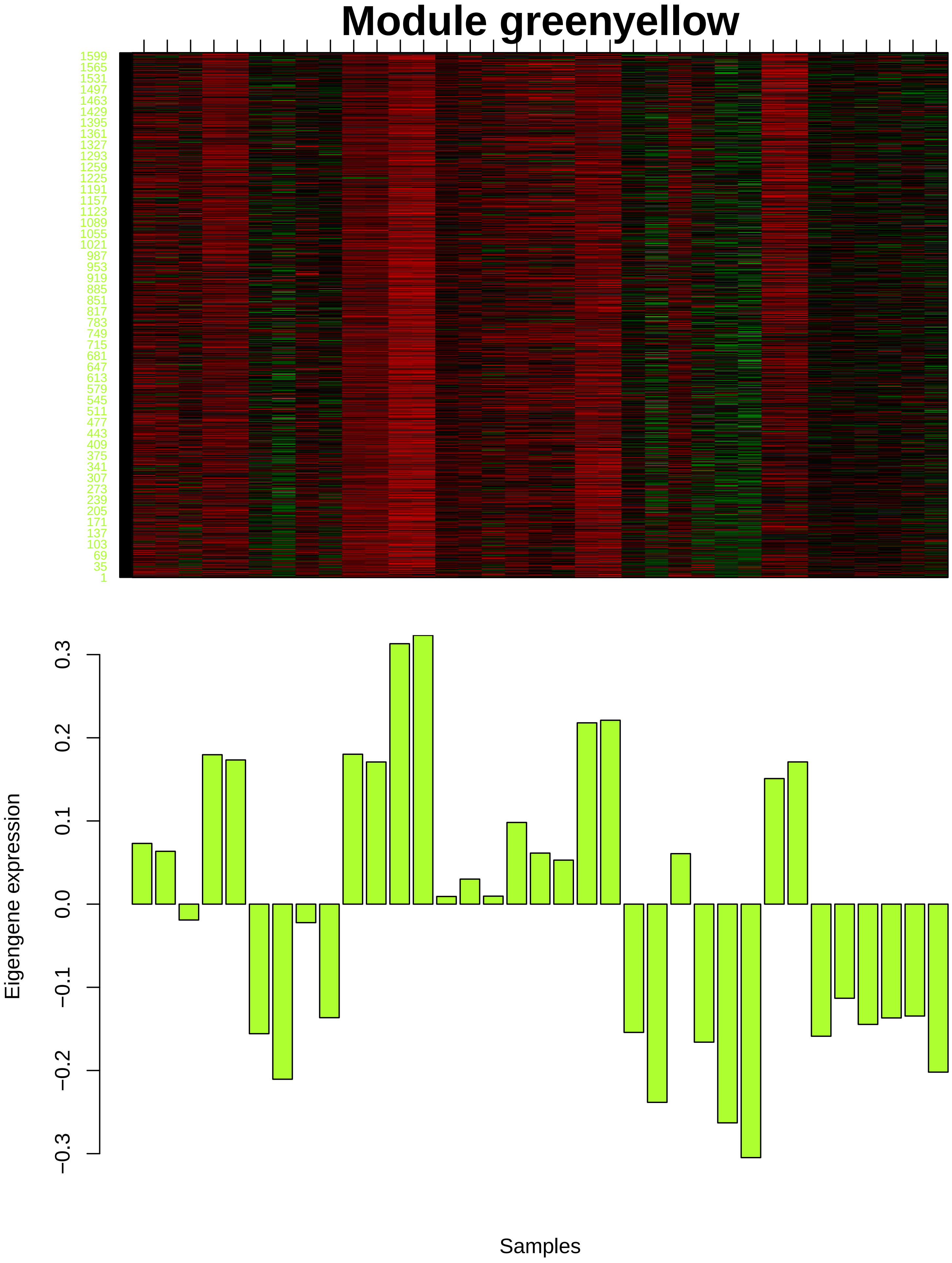

Module greenyellow
1599
1565
1531
1497
1463
1429
1395
1361
1327
1293
1259
1225
1191
1157
1123
1089
1055
1021
987
953
919
885
851
817
783
749
715
681
647
613
579
545
511
477
443
409
375
341
307
273
239
205
171
137
103
69
35
1
Eigengene expression
0.3
0.2
0.1
0.0
−0.1
−0.2
−0.3
Samples

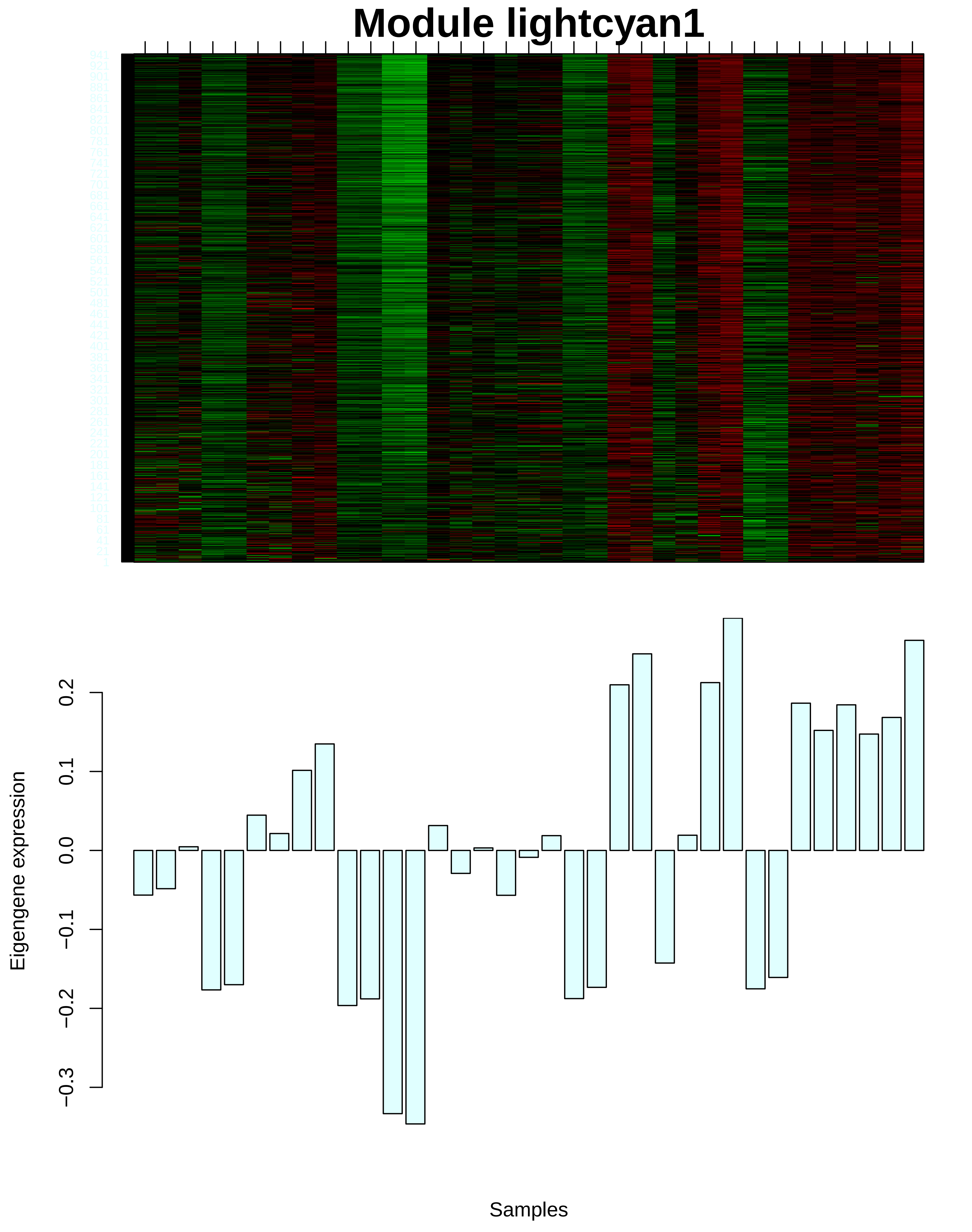
Module lightcyan1
Eigengene expression
0.2
0.1
0.0
−0.1
−0.2
−0.3
Samples

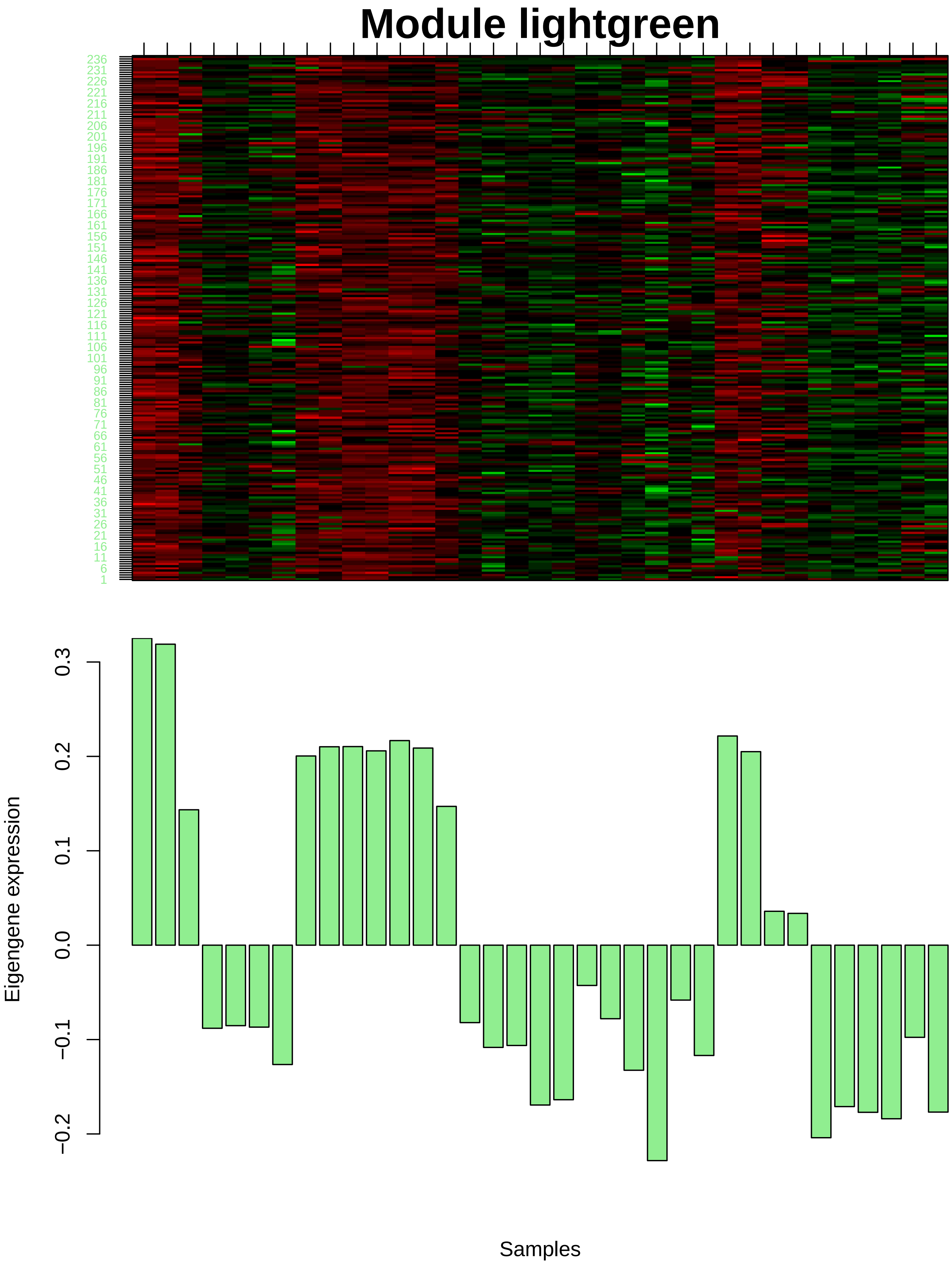

Module lightgreen
Eigengene expression
Samples

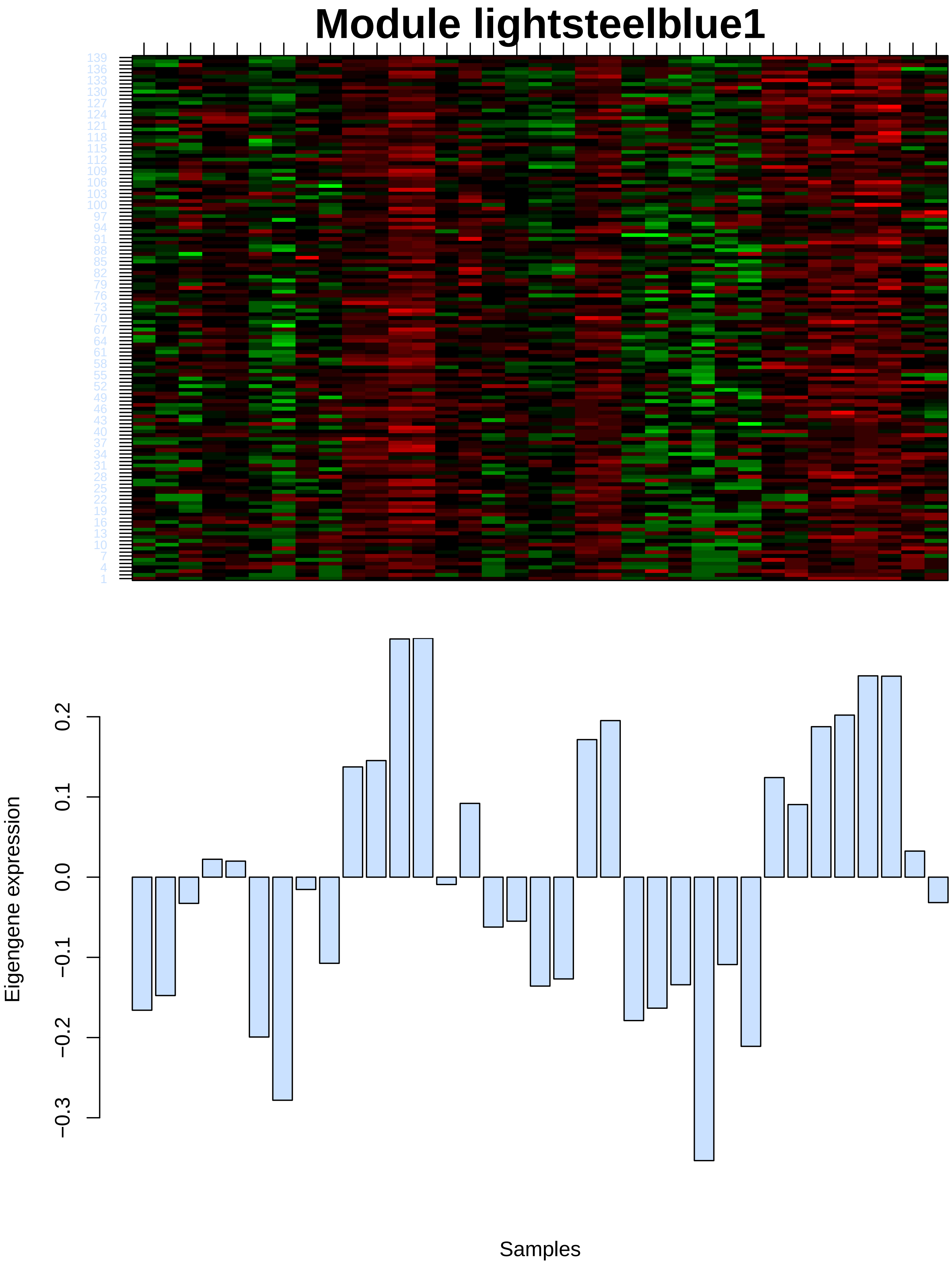

Module lightsteelblue1
Eigengene expression
0.2
0.1
0.0
−0.1
−0.2
−0.3
Samples

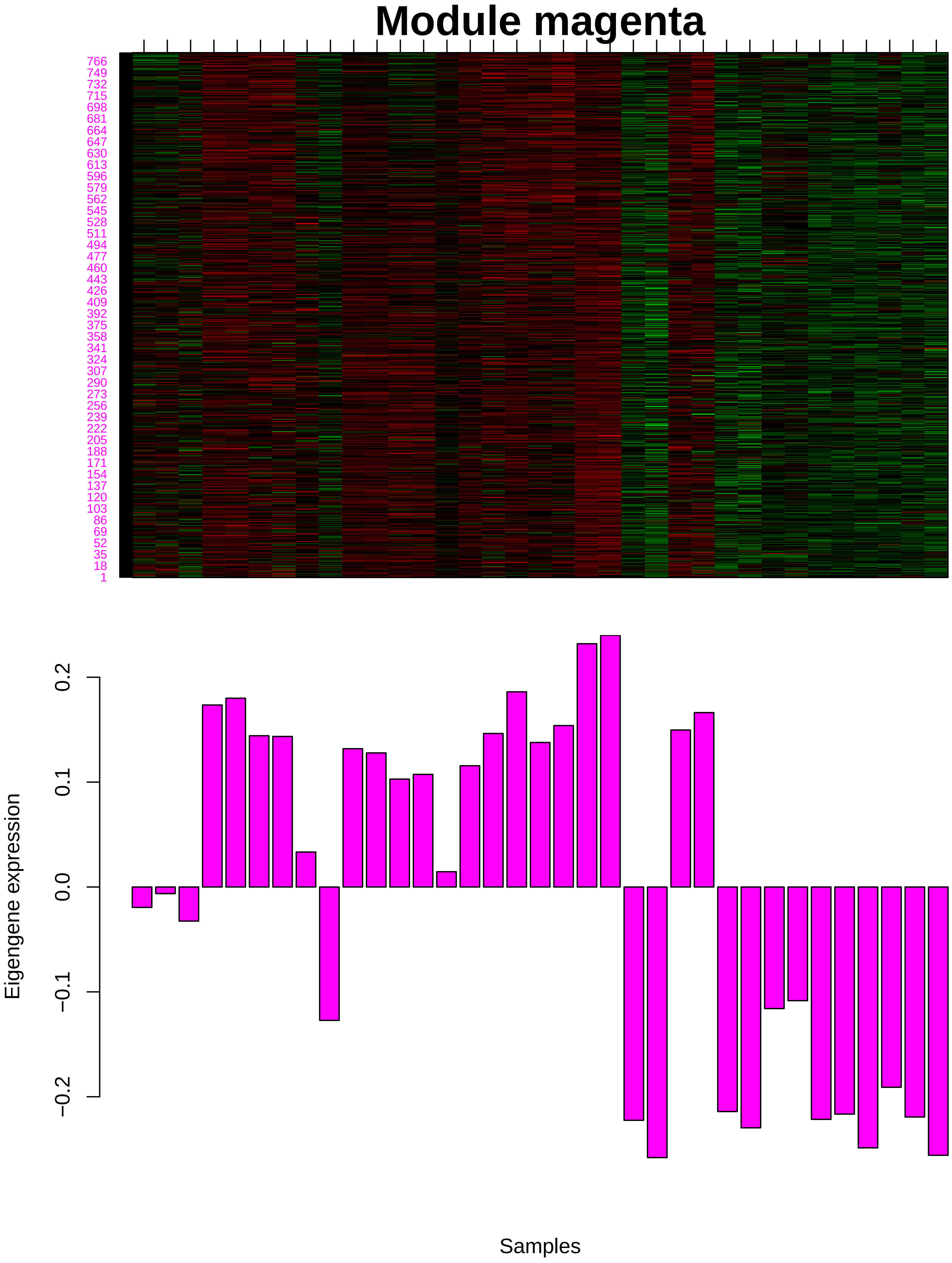

Module magenta
766
749
732
715
698
681
664
647
630
613
596
579
562
545
528
511
494
477
460
443
426
409
392
375
358
341
324
307
290
273
256
239
222
205
188
171
154
137
120
103
86
69
52
35
18
1
Eigengene expression
0.2
0.1
0.0
−0.1
−0.2
Samples

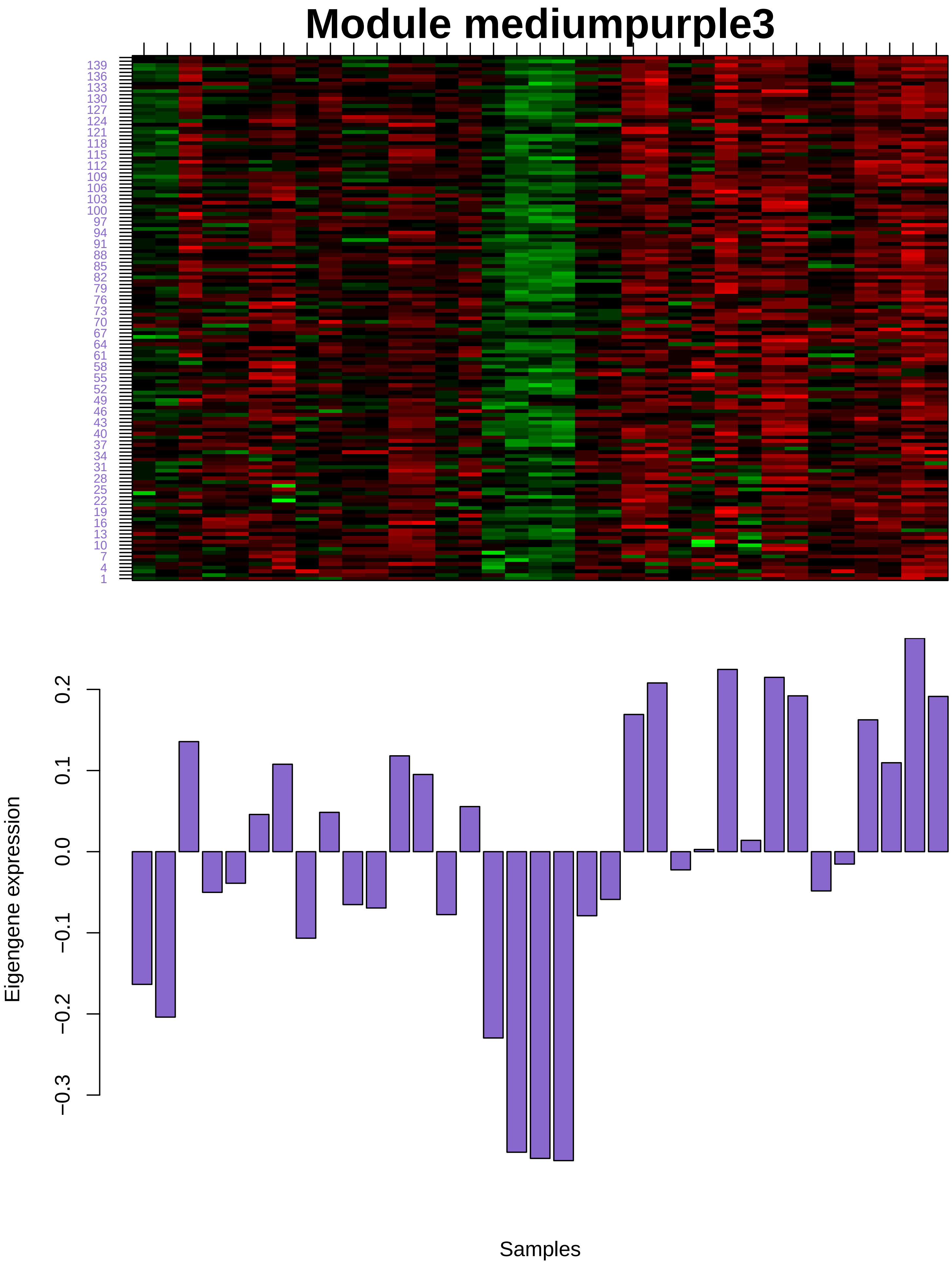

Module mediumpurple3
Eigengene expression
Samples

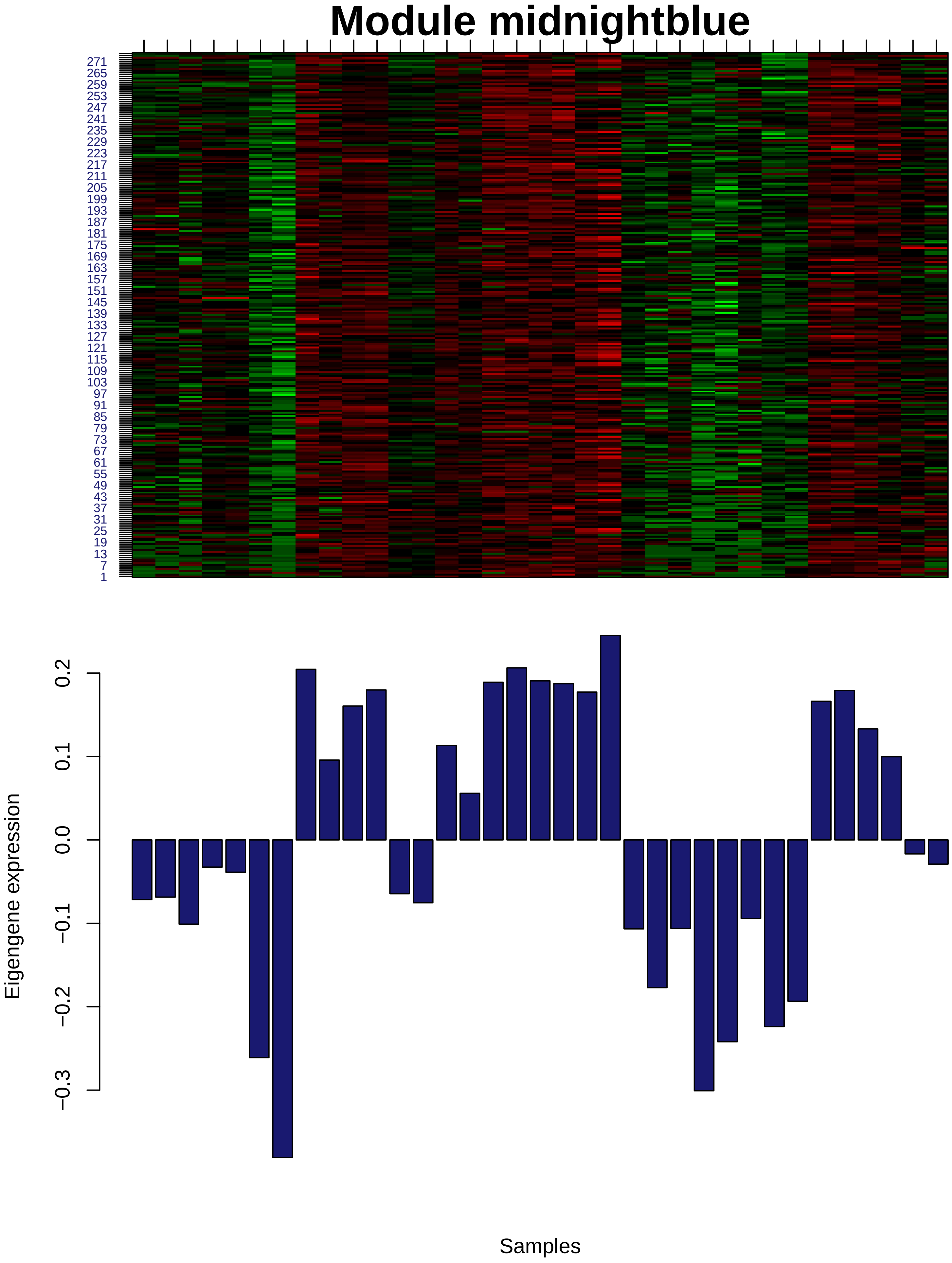

Module midnightblue
271
265
259
253
247
241
235
229
223
217
211
205
199
193
187
181
175
169
163
157
151
145
139
133
127
121
115
109
103
97
91
85
79
73
67
61
55
49
43
37
31
25
19
13
7
1
0.2
0.1
0.0
−0.1
−0.2
−0.3
Eigengene expression
Samples

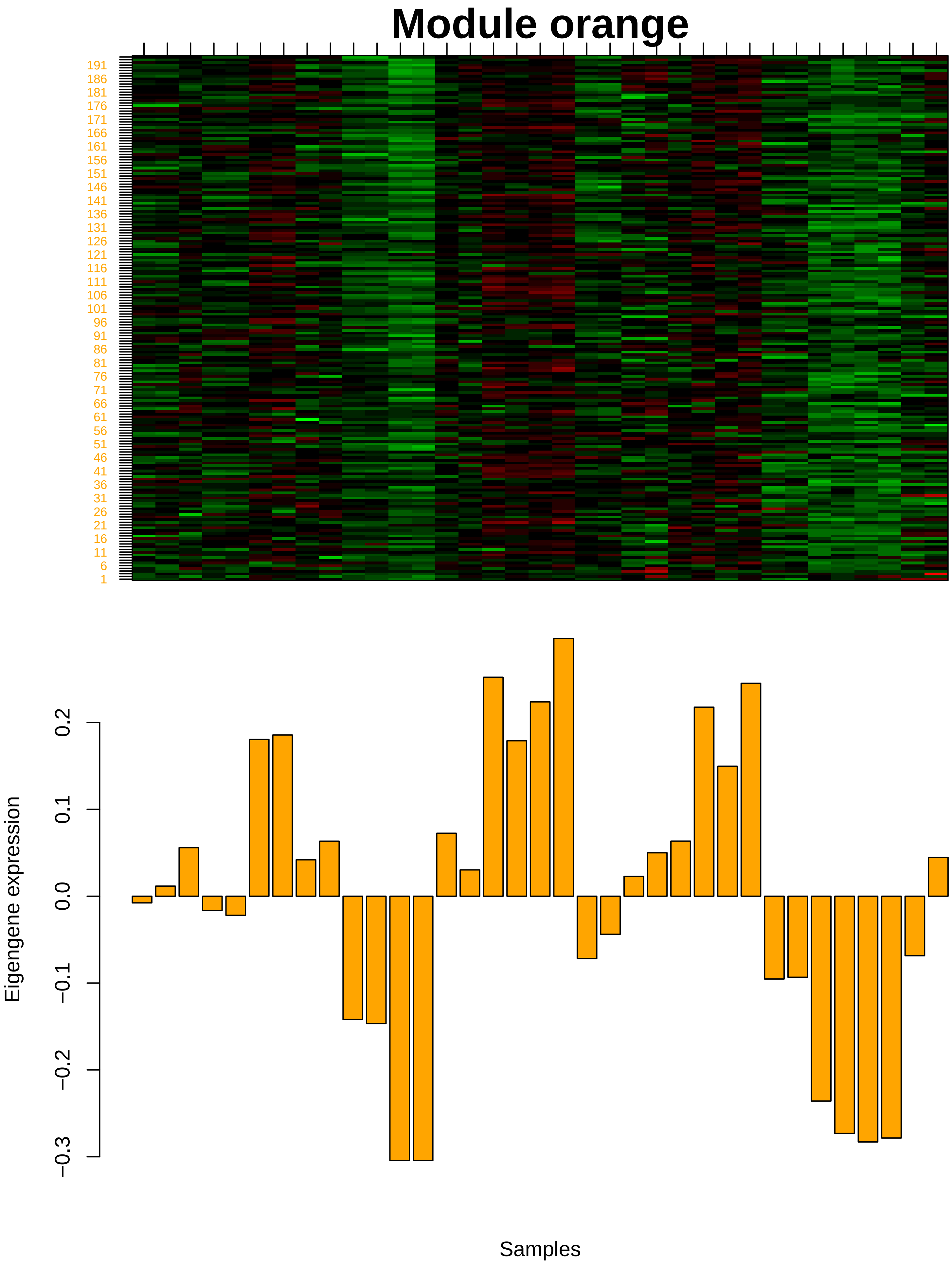

Module orange
191
186
181
176
171
166
161
156
151
146
141
136
131
126
121
116
111
106
101
96
91
86
81
76
71
66
61
56
51
46
41
36
31
26
21
16
11
6
1
Eigengene expression
0.2
0.1
0.0
−0.1
−0.2
−0.3
Samples

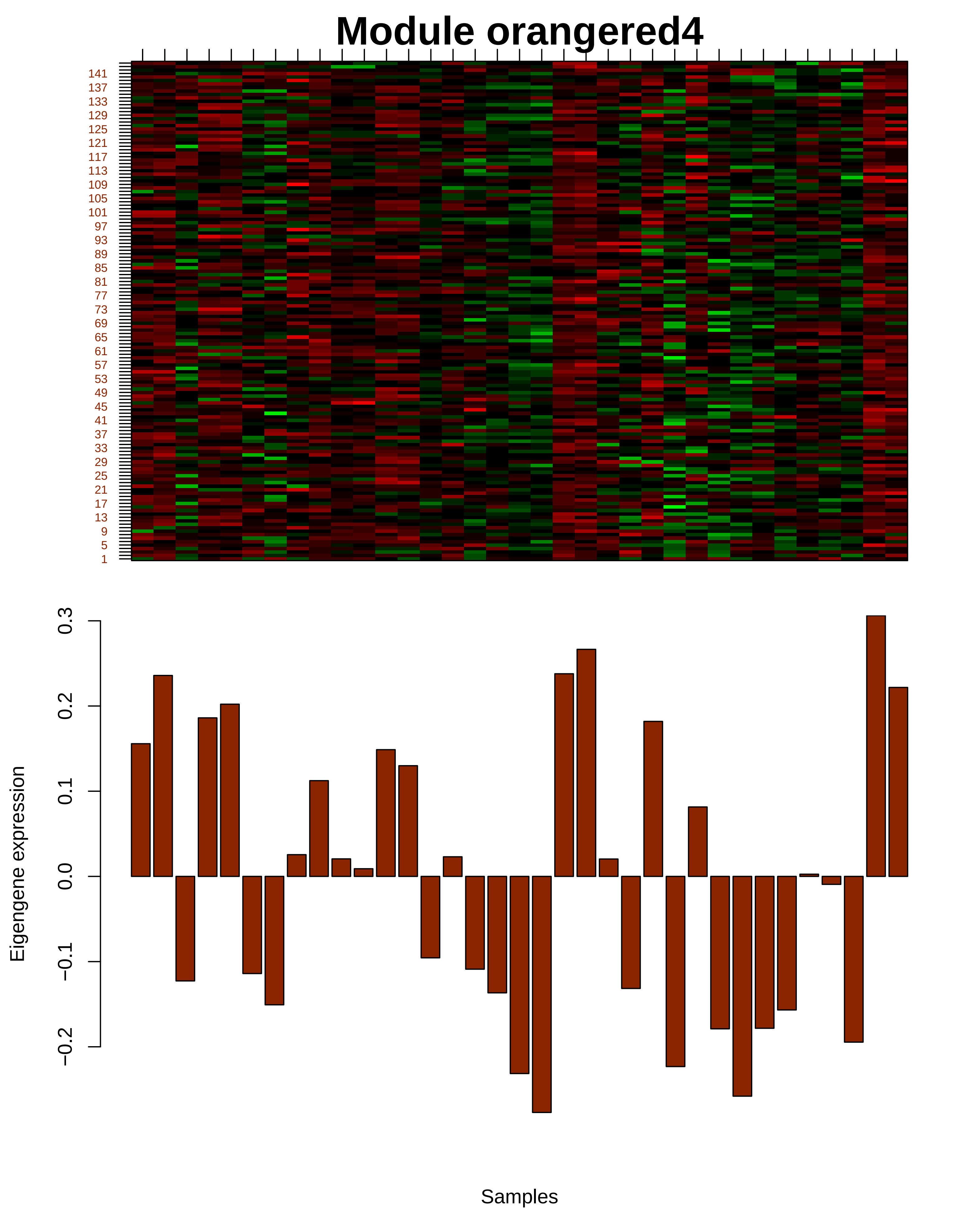

Module orangered4
Eigengene expression
Samples

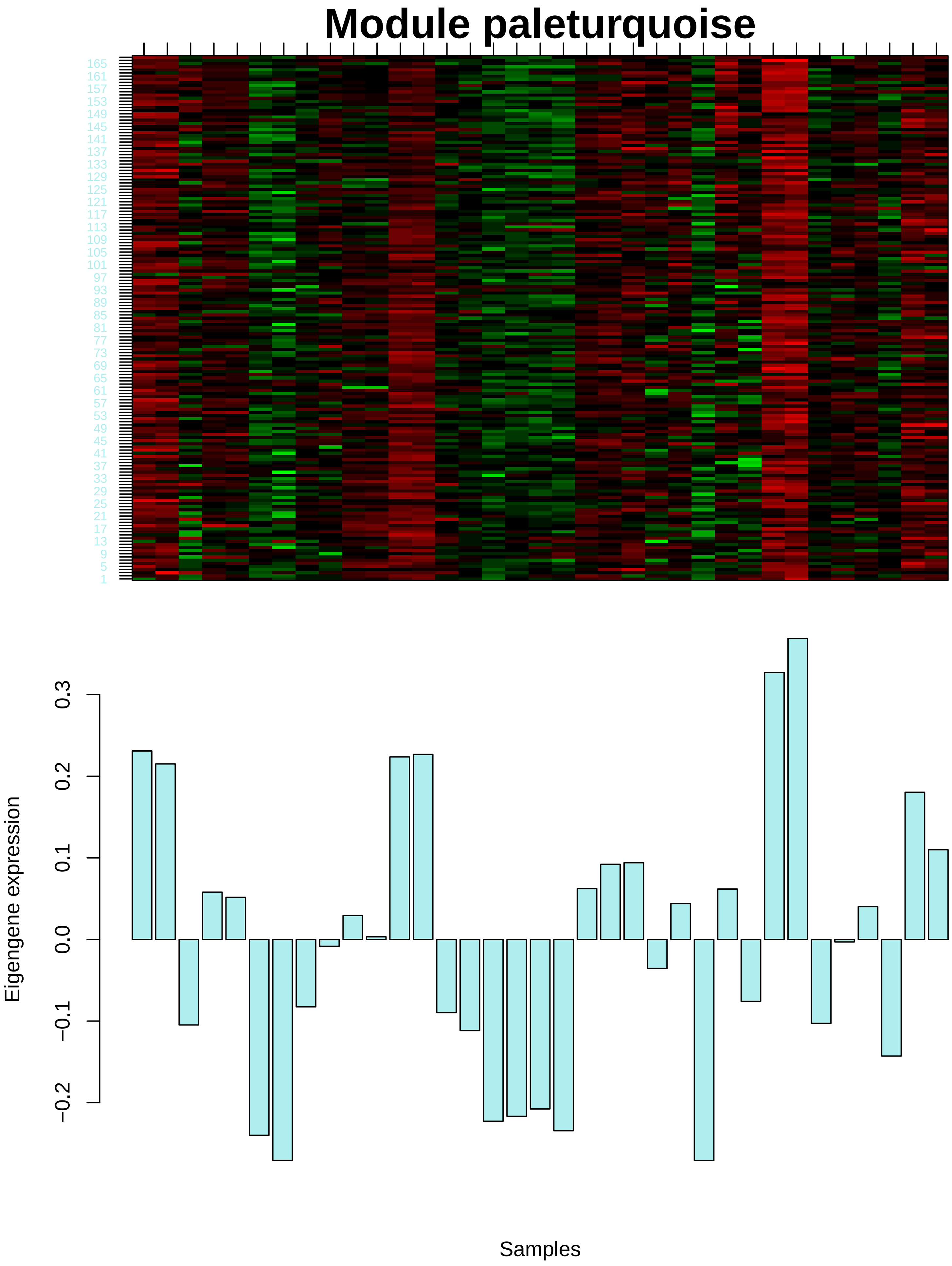

Module paleturquoise
Eigengene expression
Samples
0.3
0.2
0.1
0.0
−0.1
−0.2

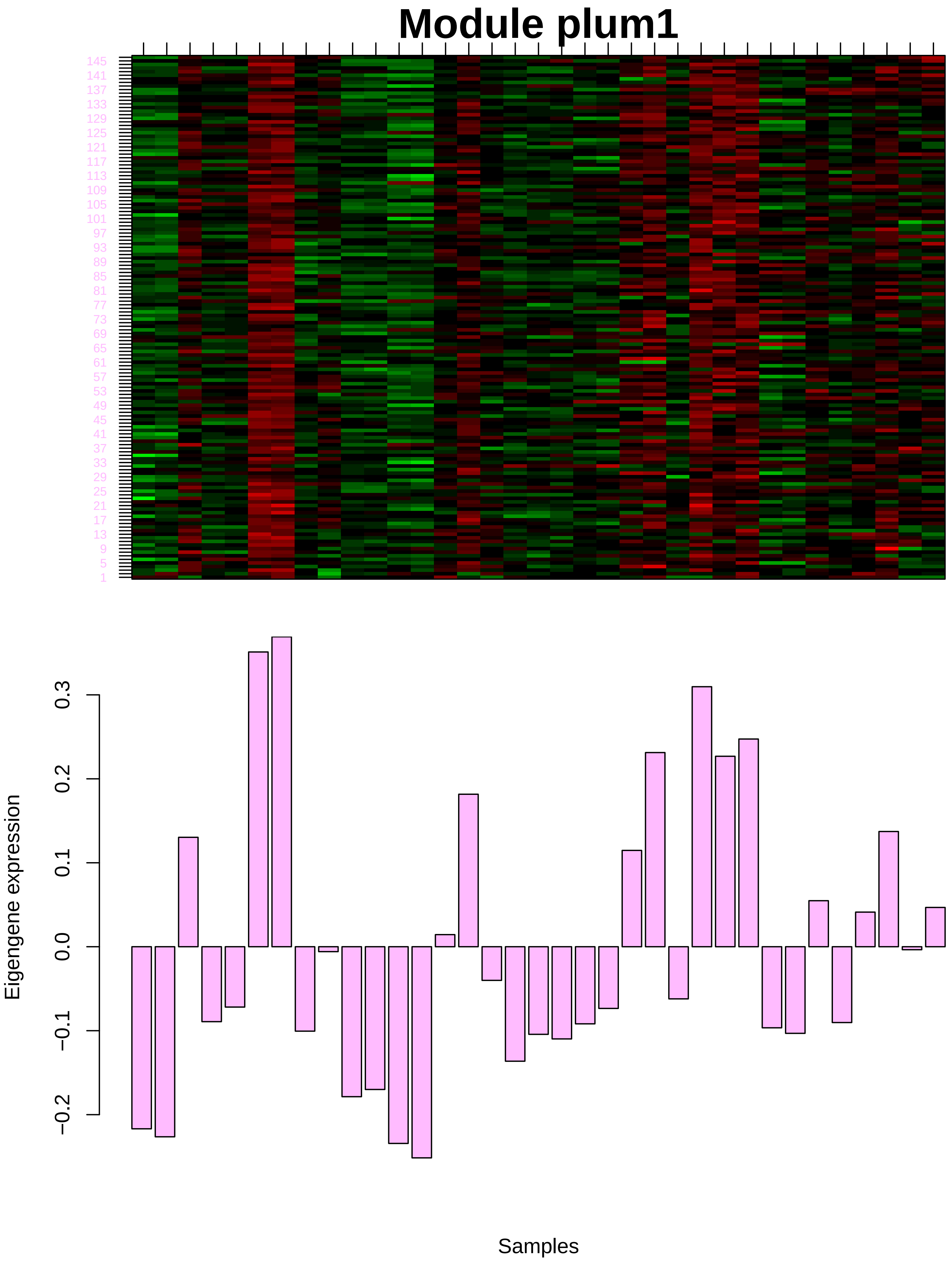

Module plum1
145
141
137
133
129
125
121
117
113
109
105
101
97
93
89
85
81
77
73
69
65
61
57
53
49
45
41
37
33
29
25
21
17
13
9
5
1
Eigengene expression
0.3
0.2
0.1
0.0
−0.1
−0.2
Samples

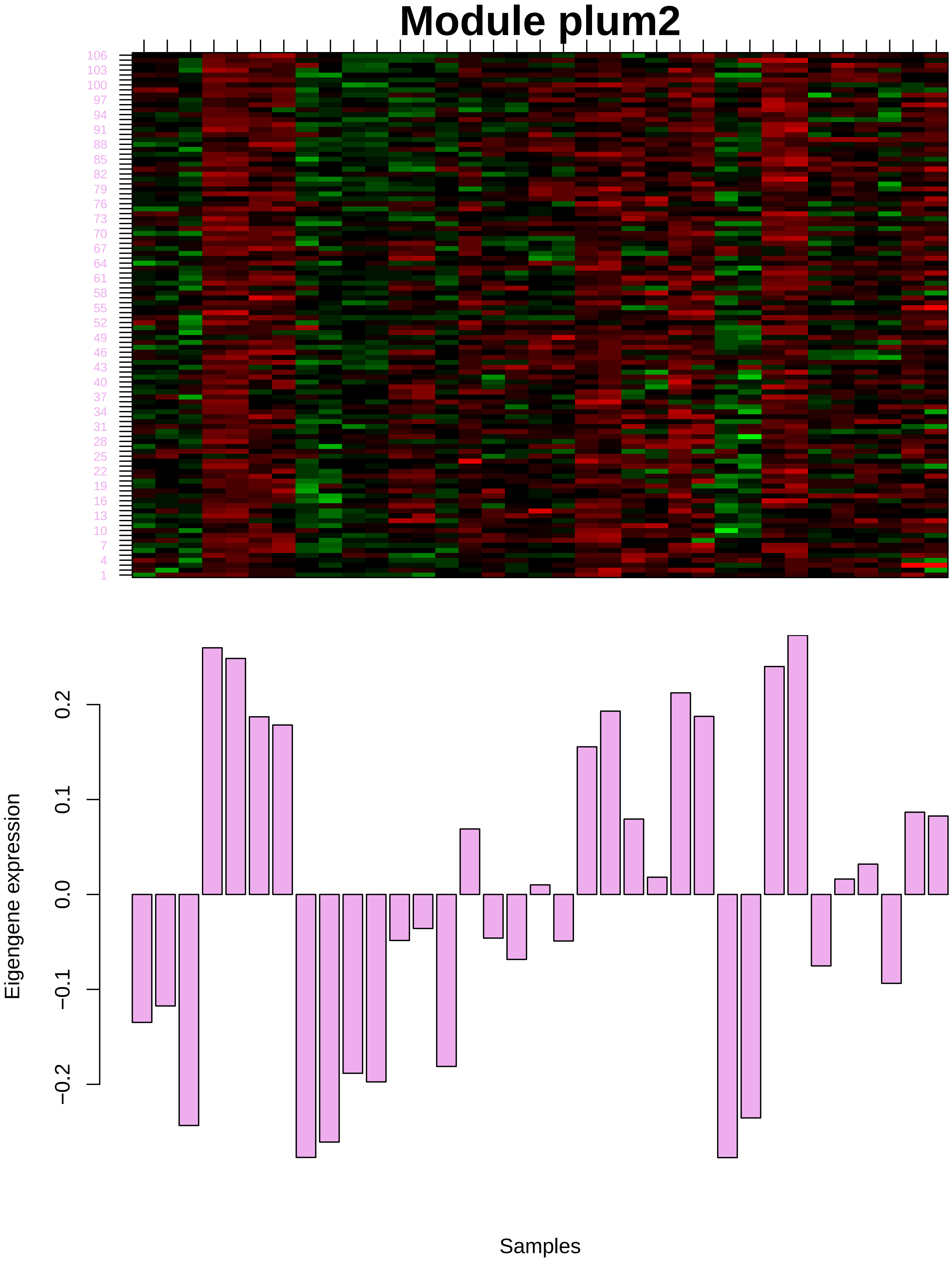

Module plum2
106
103
100
97
94
91
88
85
82
79
76
73
70
67
64
61
58
55
52
49
46
43
40
37
34
31
28
25
22
19
16
13
10
7
4
1
Eigengene expression
0.2
0.1
0.0
−0.1
−0.2
Samples

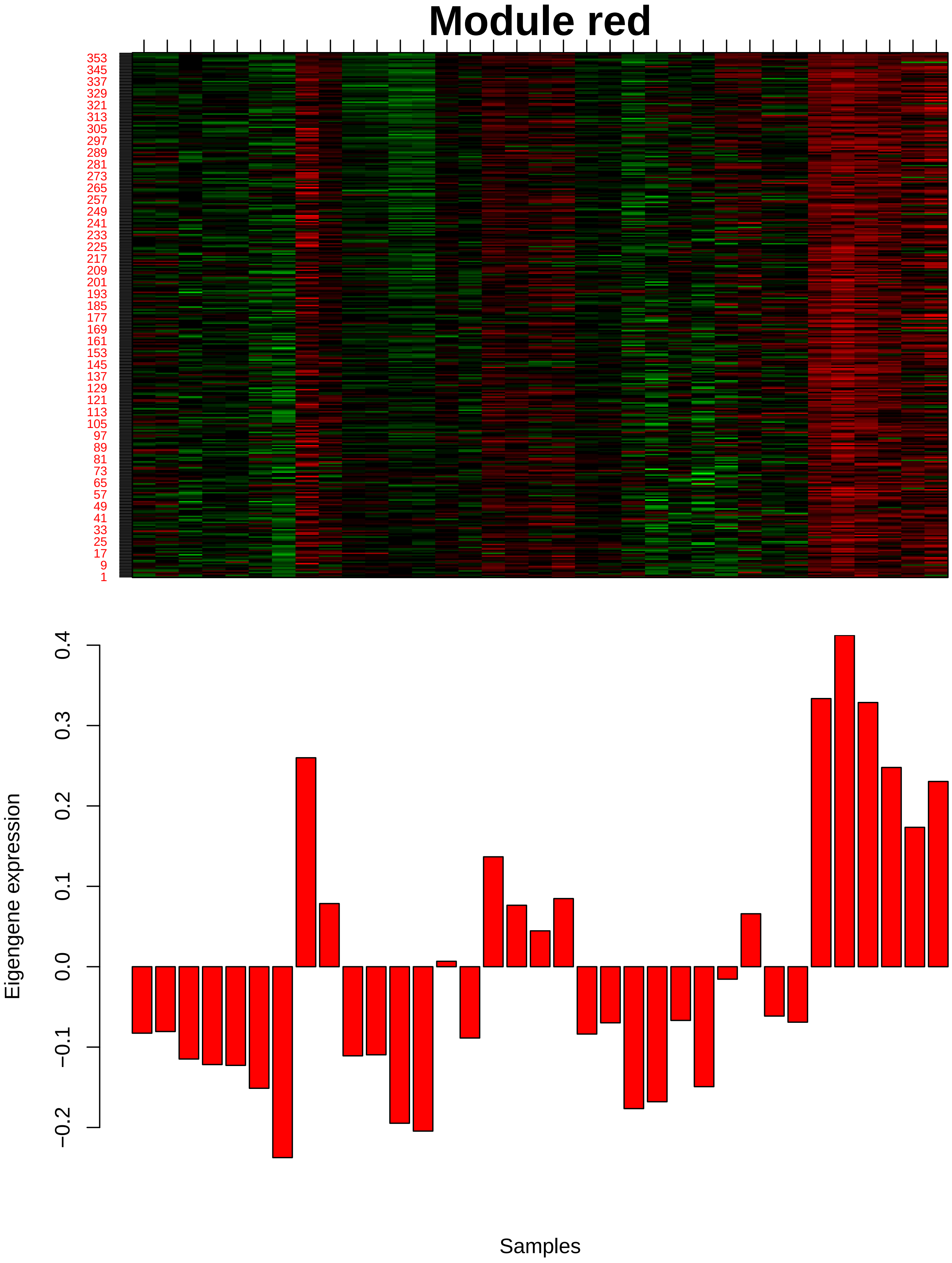
Module red
353
345
337
329
321
313
305
297
289
281
273
265
257
249
241
233
225
217
209
201
193
185
177
169
161
153
145
137
129
121
113
105
97
89
81
73
65
57
49
41
33
25
17
9
1
Eigengene expression
0.4
0.3
0.2
0.1
0.0
−0.1
−0.2
Samples

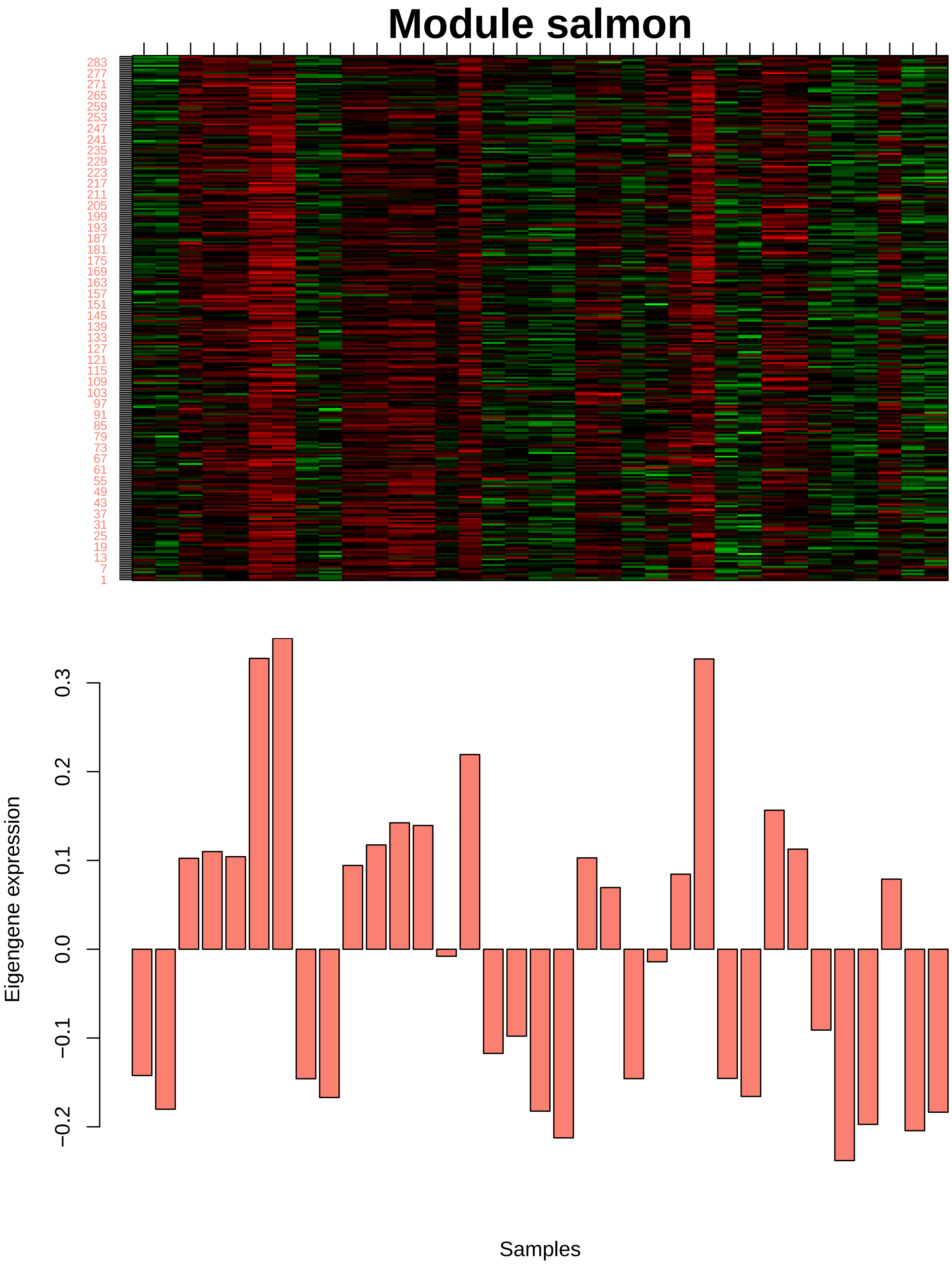

Module salmon
283
277
271
265
259
253
247
241
235
229
223
217
211
205
199
193
187
181
175
169
163
157
151
145
139
133
127
121
115
109
103
97
91
85
79
73
67
61
55
49
43
37
31
25
19
13
7
1
Eigengene expression
0.3
0.2
0.1
0.0
−0.1
−0.2
Samples

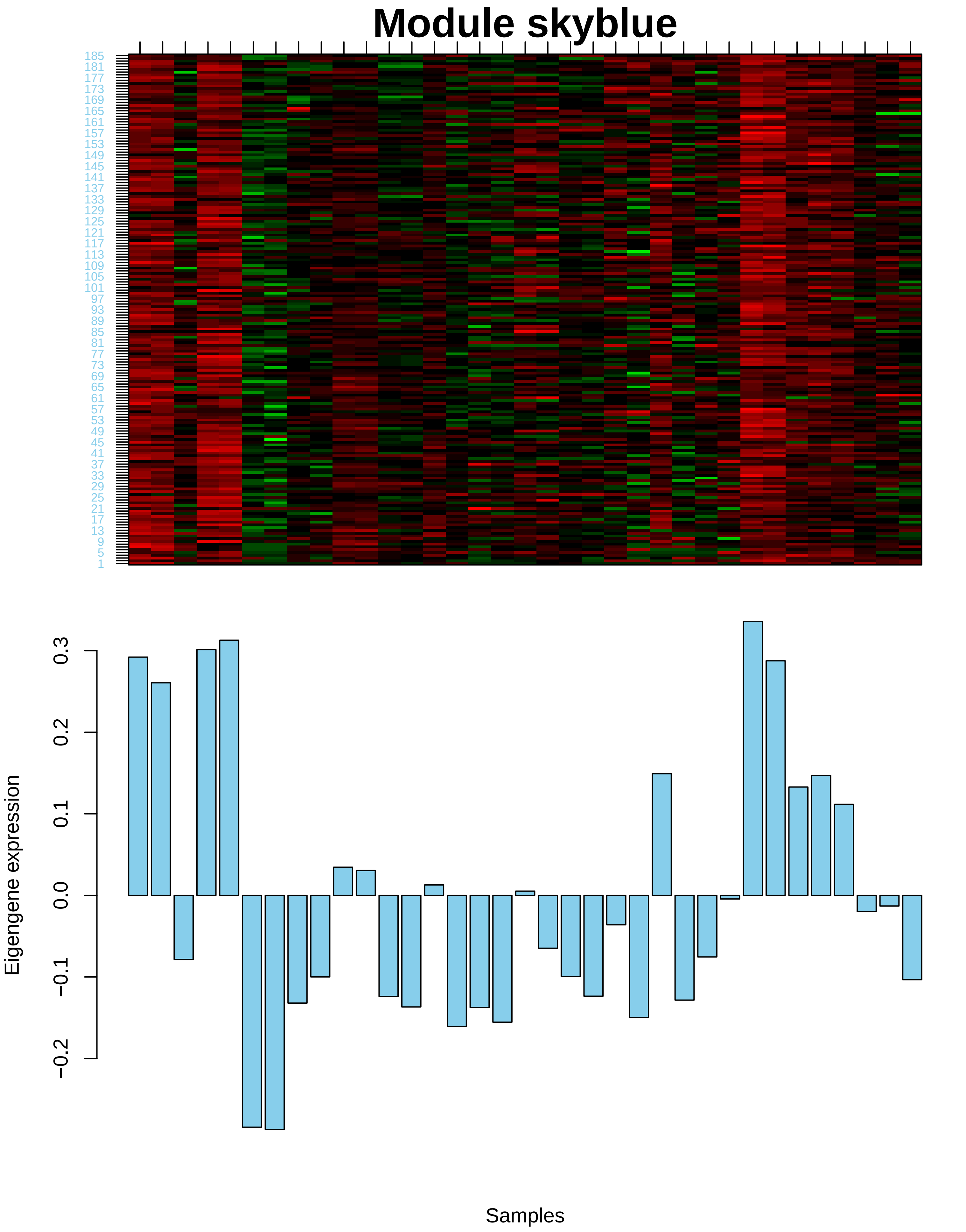
Module skyblue
185
181
177
173
169
165
161
157
153
149
145
141
137
133
129
125
121
117
113
109
105
101
97
93
89
85
81
77
73
69
65
61
57
53
49
45
41
37
33
29
25
21
17
13
9
5
1
Eigengene expression
0.3
0.2
0.1
0.0
−0.1
−0.2
Samples

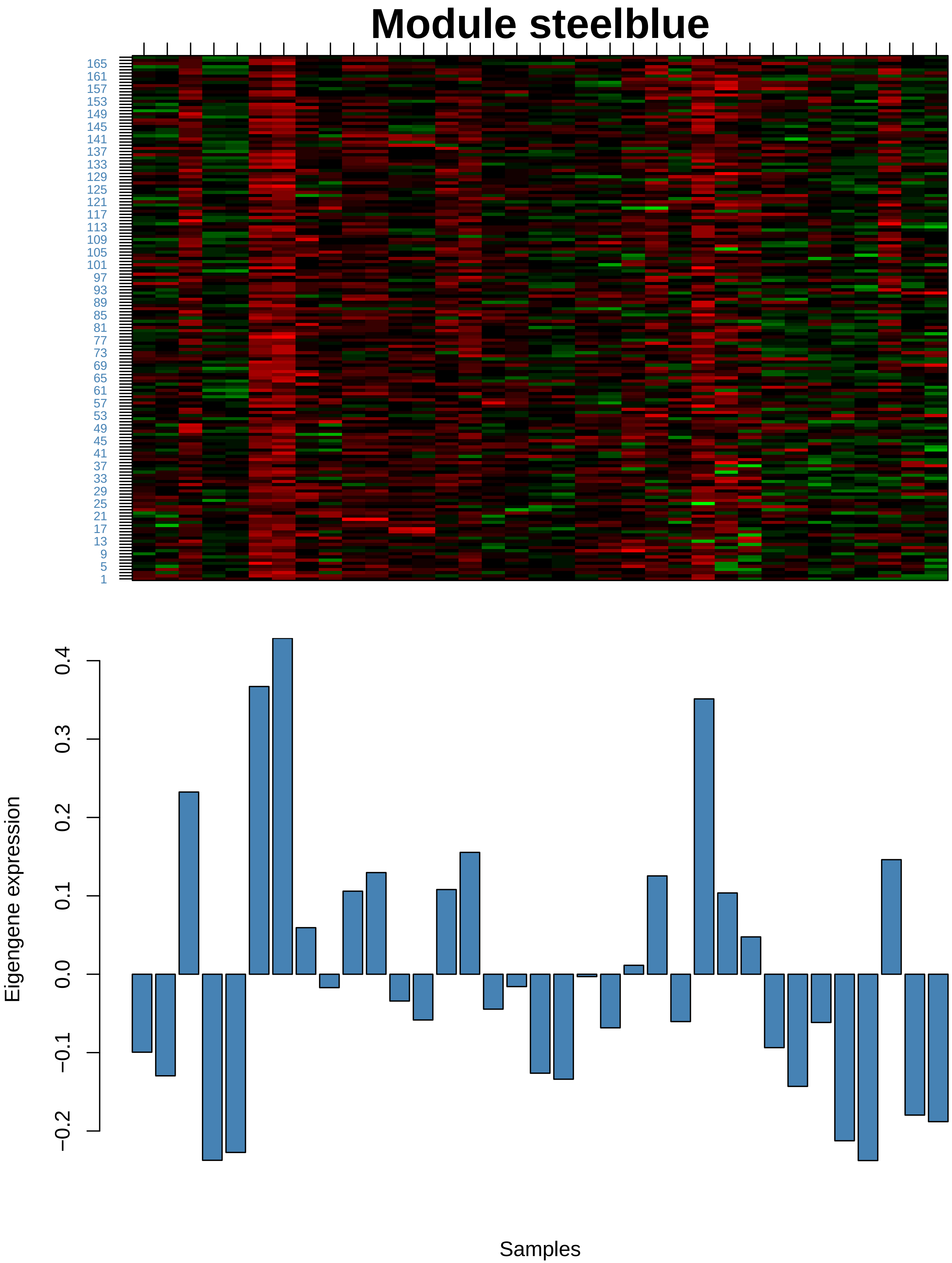
Module steelblue
165
161
157
153
149
145
141
137
133
129
125
121
117
113
109
105
101
97
93
89
85
81
77
73
69
65
61
57
53
49
45
41
37
33
29
25
21
17
13
9
5
1
Eigengene expression
0.4
0.3
0.2
0.1
0.0
−0.1
−0.2
Samples

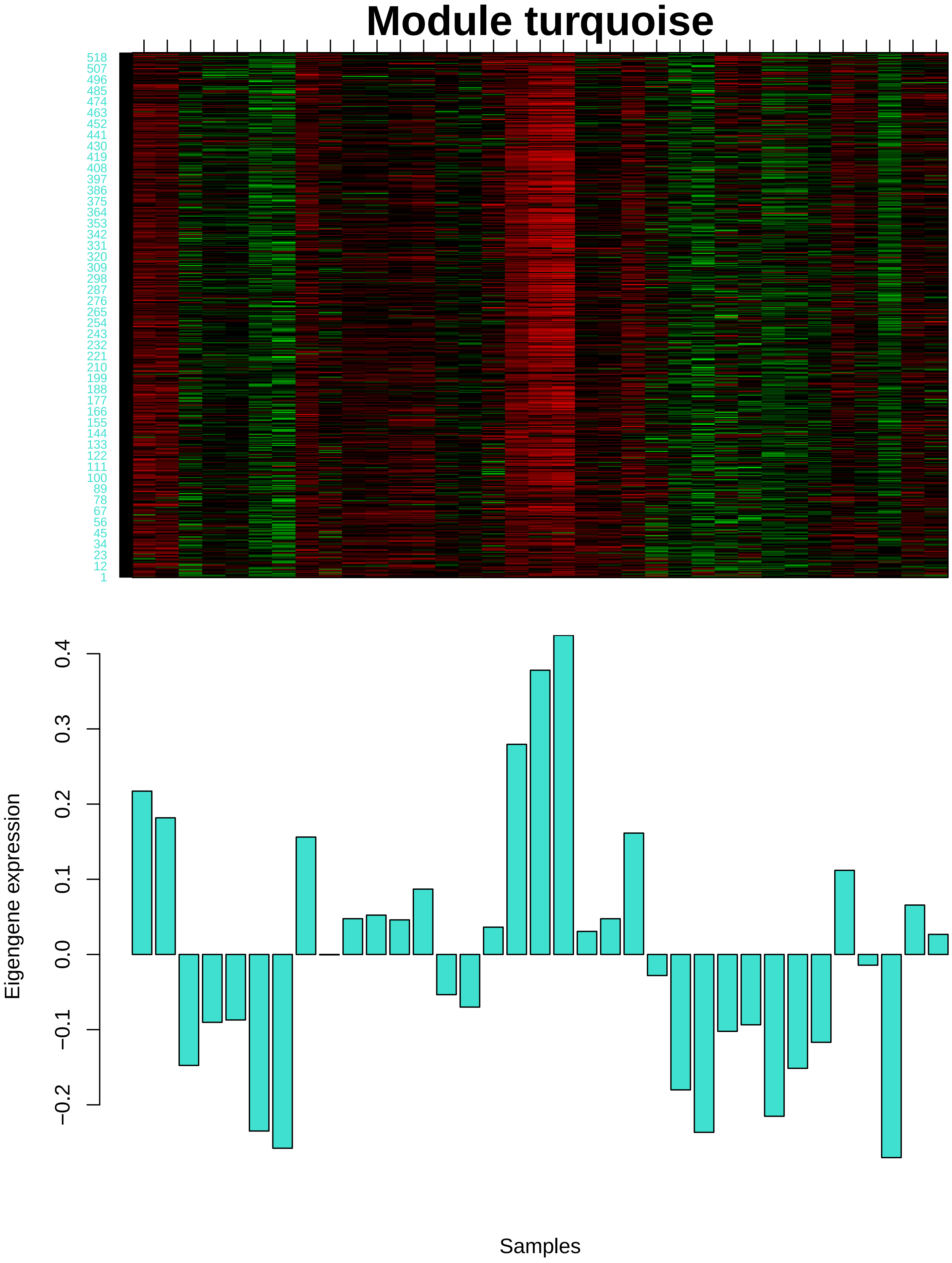

Module turquoise
518
507
496
485
474
463
452
441
430
419
408
397
386
375
364
353
342
331
320
309
298
287
276
265
254
243
232
221
210
199
188
177
166
155
144
133
122
111
100
89
78
67
56
45
34
23
12
1
Eigengene expression
0.4
0.3
0.2
0.1
0.0
−0.1
−0.2
Samples

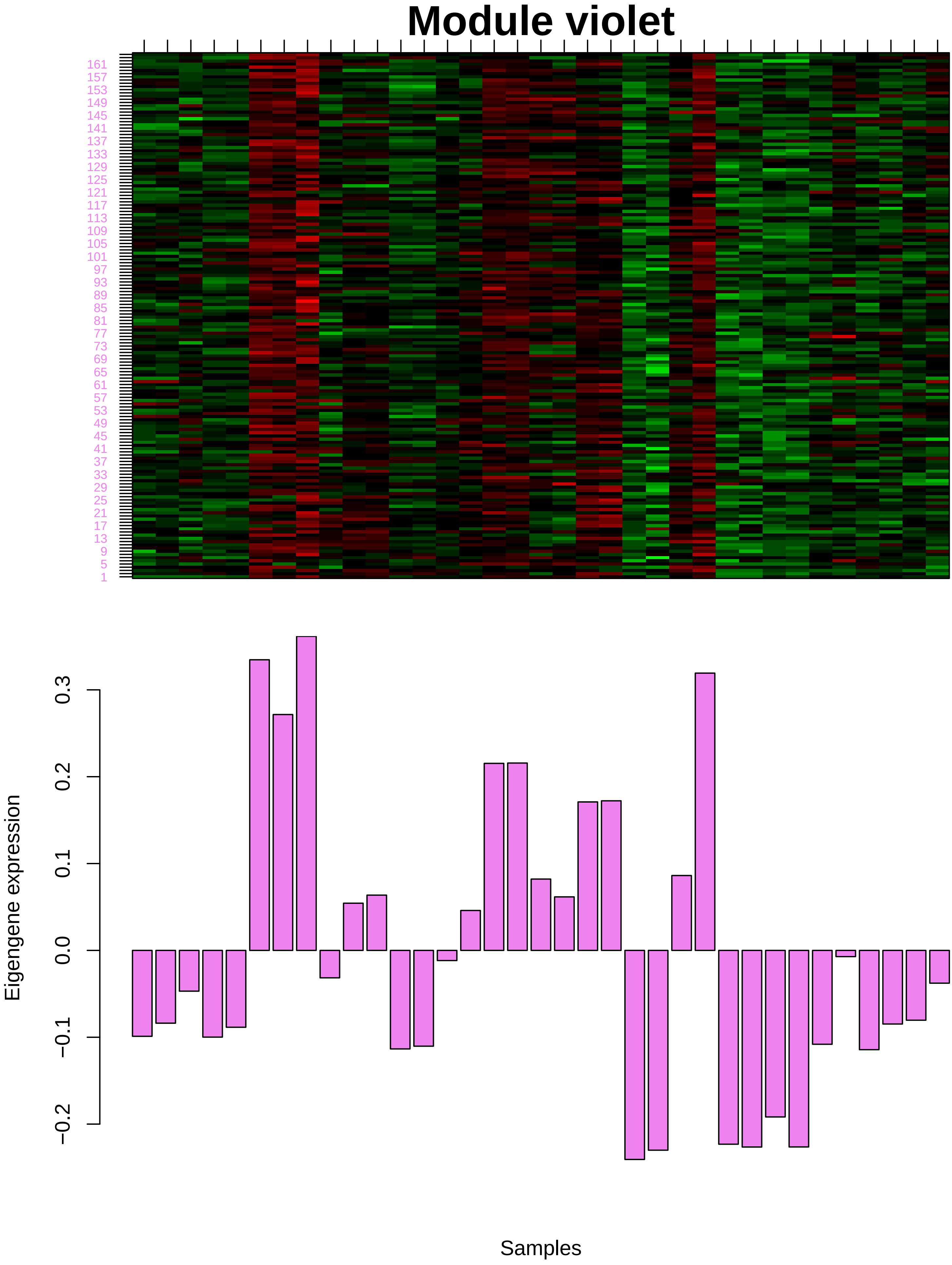

Module violet
161
157
153
149
145
141
137
133
129
125
121
117
113
109
105
101
97
93
89
85
81
77
73
69
65
61
57
53
49
45
41
37
33
29
25
21
17
13
9
5
1
0.3
0.2
0.1
0.0
−0.1
−0.2
Eigengene expression
Samples

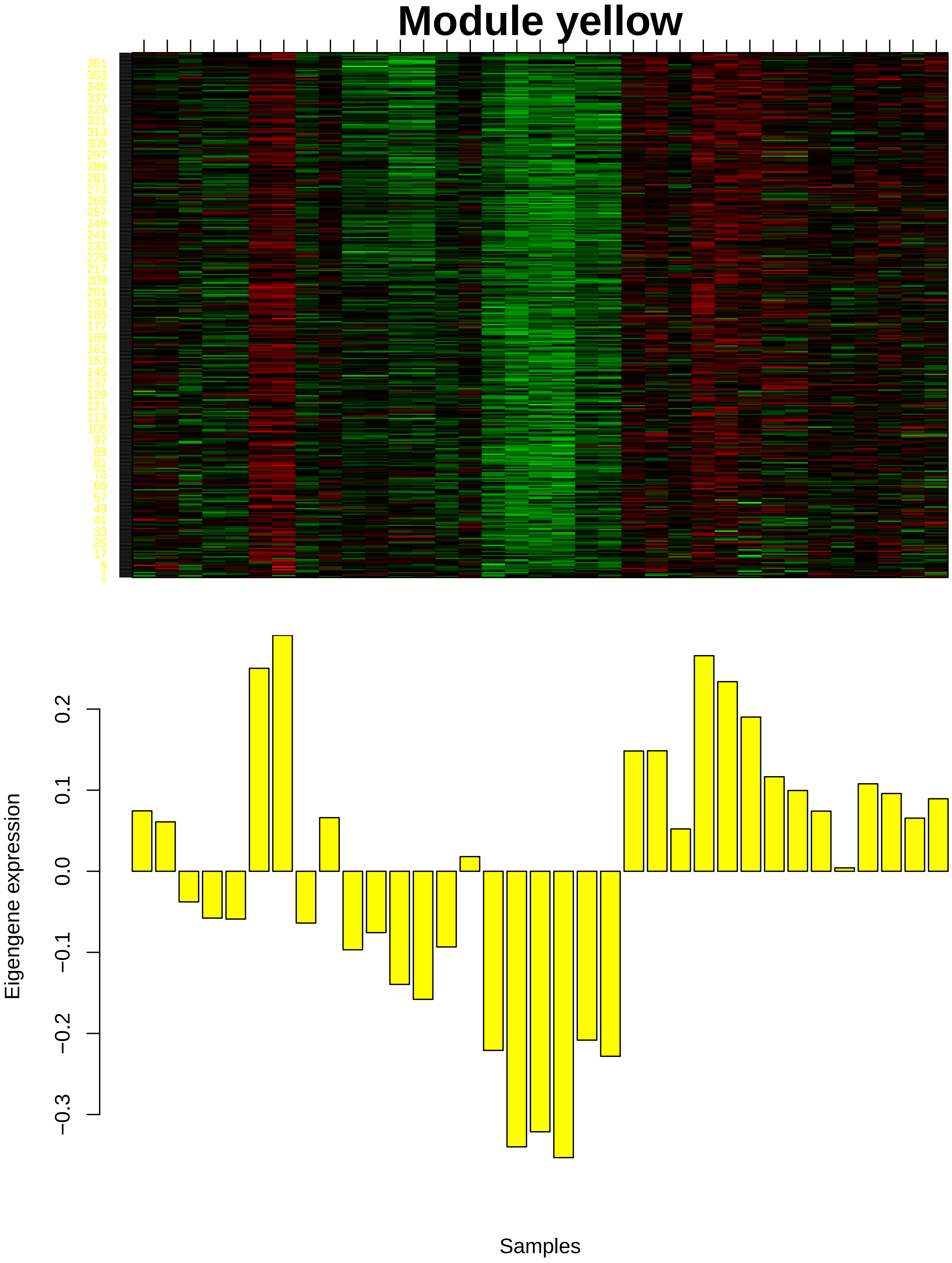

Module yellow
Eigengene expression
0.2
0.1
0.0
−0.1
−0.2
−0.3
Samples

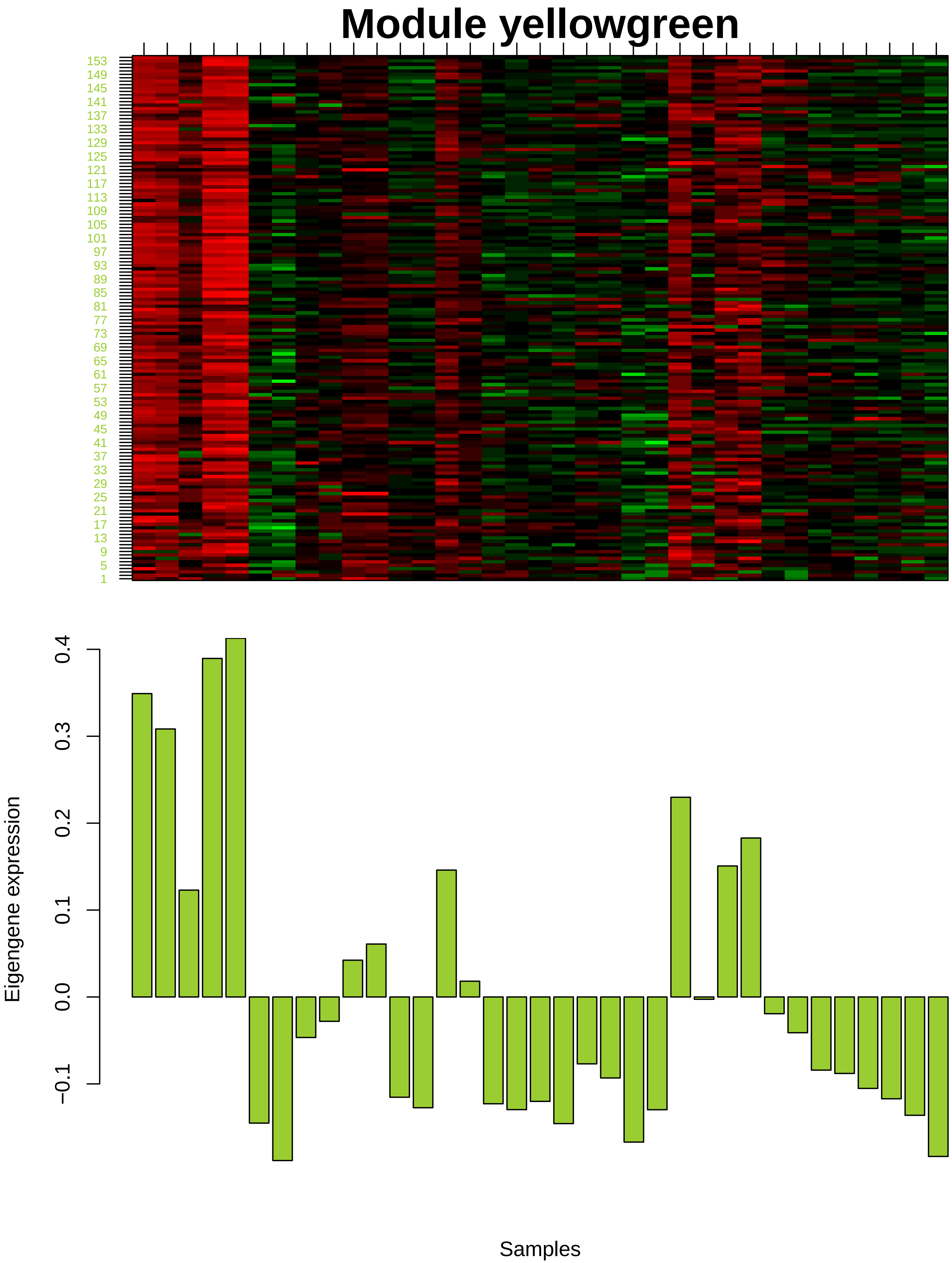
Module yellowgreen
Eigengene expression
Samples
0.4
0.3
0.2
0.1
0.0
−0.1
